# Global trends in childhood urinary tract infections, 1990–2021: results from the GBD study

**DOI:** 10.3389/fpubh.2025.1593206

**Published:** 2025-09-25

**Authors:** Xiepeng Zuo, Cheng Fang, Chuanming Wang, Ziqi Fang, Qingyuan Liang, Zhaodelong Dai, Meng Sun, Liwei Liu, Simeng Wen

**Affiliations:** Department of Urology, Tianjin Institute of Urology, The Second Hospital of Tianjin Medical University, Tianjin, China

**Keywords:** urinary tract infection, Global Burden of Disease study, incidence, mortality, childhood

## Abstract

**Background:**

Urinary tract infections (UTIs) represent a prevalent urological disorder in childhood populations with substantial clinical implications. This study reports global trends from 1990 to 2021 in incident cases, incidence rates, mortality counts, mortality rates, disability-adjusted life years (DALYs), and DALY rates attributable to childhood UTIs.

**Methods:**

Utilizing Global Burden of Disease (GBD) 2021 data, we analyzed incidence, mortality, and DALY rates (per 100,000 population) with 95% uncertainty intervals (UI) for children aged 0–14 years. Data spanned 204 countries and territories, stratified by age, sex, and geographic location. Temporal trends were quantified using: Segmented regression to compute Annual Percentage Change and Average Annual Percentage Change. Log-linear regression models to derive Estimated Annual Percentage Change with 95% confidence intervals (CI). Associations between disease burden indicators and the Socio-demographic Index (SDI) were characterized using generalized additive models (GAMs) to capture potential nonlinear relationships.

**Results:**

From 1990 to 2021, global incident cases of childhood UTIs increased by 10.31% (95% UI: 4.33–13.82), whereas the global incidence rate decreased by 4.65% (95% UI: −9.82 to −1.60). Concurrently, downward trends were observed in deaths, DALYs, mortality rates, and DALY rates. Notably, childhood UTIs burden demonstrated significant associations with regional socioeconomic development and environmental conditions. In low-SDI regions, incident cases surged by 63.43% (95% UI: 47.21–76.20). This starkly contrasts with the declines observed in high-middle and high-SDI regions. These findings underscore the elevated UTIs incidence rates in tropical countries, necessitating targeted resource allocation for prevention and clinical management.

**Conclusion:**

From 1990 to 2021, the global incidence rate of childhood UTIs exhibited a downward trend. However, this trend reversed over the past decade, with a marked increase in incidence rates. Significant disparities in incidence rates were observed across population groups globally, stratified by sex, age, geographical location, and socioeconomic status. The incidence rate of UTIs is higher among children in socioeconomically disadvantaged and tropical regions. A representative example is the sustained increase in UTIs incidence among children in South Asia. Conversely, incidence rates were higher in girls, while mortality and DALYs were significantly elevated in boys. To optimize resource allocation and ensure essential treatment reaches those in need, governments and health organizations must tailor interventions based on regional and population-specific burdens. These findings underscore the necessity of developing effective, tailored prevention and treatment strategies.

## Introduction

Urinary tract infections (UTIs) represent a prevalent global health burden with significant clinical implications. Contrary to the clinical perception of UTIs as relatively uncommon, epidemiological evidence confirms their high prevalence and substantial mortality. The economic impact is considerable, with substantial healthcare expenditures allocated annually to UTIs management and complication mitigation worldwide. Etiologically, bacterial pathogens dominate, with *Escherichia coli* implicated in 80–90% of community-acquired uncomplicated acute pyelonephritis cases (1).

UTIs represent prevalent urological disorders in childhood populations with substantial clinical significance. These conditions are associated not only with acute complications but also with potential long-term sequelae. As one of the most frequent bacterial infections during childhood—particularly among infants and young children—UTIs exhibit markedly higher incidence rates in girls (2). By 7 years of age, UTIs affect approximately 1.7% of boys and 8.4% of girls (3). Nevertheless, a significant data gap remains in global epidemiological data on childhood UTIs, substantially impeding the development and implementation of evidence-based public health policies.

The global burden of childhood UTIs impedes progress toward United Nations Sustainable Development Goals (SDGs) targets. Premature mortality and reduced life expectancy from pediatric UTIs directly hinder SDG 3 (Ensure healthy lives). Moreover, marked disparities in UTI burden across development strata reveal systemic weaknesses. Inadequate strengthening of primary healthcare systems in low- and middle-income countries (LMICs) exacerbates health inequities, thus contravening the core equity principle of SDG 10 (Reduced inequalities). Consequently, reducing childhood UTIs burden necessitates not only clinical interventions but also synergistic advancement of SDGs through cross-sectoral collaboration, including investments in health infrastructure and primary care workforce development.

Despite numerous regional studies on incidence and mortality, a comprehensive epidemiological understanding of childhood UTIs—including its key determinants—remains lacking. This scarcity of robust global and subregional data impedes the design of targeted interventions and challenges the formulation of effective, evidence-based global strategies.

This study provides a comprehensive epidemiological analysis of childhood UTIs from 1990 to 2021 across global, regional, and national levels. It quantifies temporal trends in core burden indicators—incidence, mortality, and disability-adjusted life years (DALYs)—with significant public health implications. Through systematic analysis of Global Burden of Disease (GBD) 2021 data, we identified substantial geographic disparities in childhood UTIs epidemiology. Key determinants elucidated include immunocompromised states, congenital anomalies of the kidney and urinary tract (CAKUT), comorbid chronic conditions, and socioeconomic factors captured by the Sociodemographic Index (SDI). This evidence base advances the understanding of pediatric urological disorders and provides a foundation for evidence-based health policies. The findings have direct applications for improving early detection protocols and designing targeted interventions to mitigate the global childhood UTIs burden.

## Methods

### Overview and methodological details

GBD study constitutes an international research initiative designed to quantify and compare health losses attributable to diseases, injuries, and risk factors worldwide. Leveraging the most current epidemiological data and standardized methodologies, GBD delivers comprehensive assessments of health burdens across 371 countries and territories, encompassing 88 diseases and injuries alongside 204 risk factors (4). By systematically quantifying attributable health losses, GBD generates critical evidence for tracking global health trends and informing evidence-based health policy formulation (5). The GBD database delivers globally comparable, temporally complete, and granularly stratified data, establishing a scientifically validated and internationally benchmarked resource for burden estimation and health disparity assessments (6, 7).

Data for this analysis were sourced from the GBD database.^1^ The GBD study integrates diverse data inputs—including vital registration systems, population-based surveys, hospital discharge records, and disease surveillance reports—through standardized Bayesian modeling frameworks (8). In the GBD analysis, the quantification of disease burden is achieved through three key indicators: incidence, mortality, and DALYs. DALYs are calculated as the sum of years of life lost due to premature death (YLL) and years of life lived with disability (YLD). The specific formulas used are as follows:


$$\begin{array}{l} \mathrm{YLL}=\text{Number of deaths}\times \text{Standard life}\;\\ \;\;\;\;\;\;\;\;\;\;\;\;\;\;\;\;\text{expectancy}\;\mathrm{at}\;\text{the}\;\mathrm{age}\;\text{of death} \end{array}$$

YLD=Prevalence of the condition×Disability weight

Disability weights were assigned through structured expert consensus, with values scaled from 0 (perfect health) to 1 (equivalent to death). This standardized metric enables robust quantification of global disease burden. For this analysis, we extracted and analyzed GBD 2021 data on incident cases, incidence rates, mortality, and DALYs attributable to UTIs in children aged 0–14 years (1990–2021). Stratified analyses encompassed:

Sex: Male/Female.

Age groups: <1 year, 1–4 years, 5–9 years, 10–14 years.

Geographic granularity: National, regional, and global levels.

As an observational analysis of de-identified aggregate data, this study describes spatiotemporal disease patterns without collecting personally identifiable information.

The GBD database employs developmentally stratified age groupings for pediatric data, designed to align with key physiological milestones and infection risk transitions during childhood while ensuring statistical robustness through minimum population thresholds (>50,000 per stratum). This grouping methodology—based on GBD data resolution protocols—optimizes characterization of intrinsic epidemiological patterns in childhood UTIs, thereby generating precise, clinically actionable insights for public health interventions.

### Sociodemographic index

The SDI—a composite metric quantifying national and regional socioeconomic development—integrates three standardized components: lag-distributed income per capita, mean years of educational attainment among individuals aged ≥15 years, and total fertility rates for females under 25 years. SDI values range continuously from 0 (lowest development) to 1 (highest development), enabling quintile-based stratification into low, low-middle, middle, high-middle, and high development tiers (9). This classification system facilitates rigorous analysis of socioeconomic determinants and geospatial disparities in childhood UTIs burden across development gradients.

### Frontier analysis

Frontiers represent optimal health attainment levels achievable at specific sociodemographic development tiers (e.g., Sociodemographic Index quintiles), establishing theoretical benchmarks for maximal health system efficiency. For countries below these frontiers, the efficiency gap—quantified as the difference between observed health outcomes and frontier-predicted optima—reveals actionable improvement potential. This study integrates frontier analysis with the GBD framework to evaluate relative health system efficiency across nations, focusing on SDI-health outcome relationships. Specifically, we measure efficiency shortfalls using the frontier gap metric, where larger values indicate greater inefficiency in converting developmental resources into health gains (10).

### Statistical analysis

Incidence, mortality, and DALY rates (per 100,000 population) with 95% uncertainty intervals (UI) were derived from Global Burden of Disease (GBD) data. Temporal trends were quantified using Average Annual Percentage Change (AAPC), computed via the R segmentedpackage to measure average burden change rates. Annual Percentage Change (APC) and Estimated Annual Percentage Change (EAPC) were calculated through log-linear regression:


ln(y)=β0+β1t+∈

Where APC/EAPC = 100 × (eβ1 − 1)%. AAPC aggregates segment-specific APCs weighted by interval duration. Trend directionality was determined by 95% confidence intervals (CI):

Significant increase: EAPC and CI lower limit >0.

Significant decrease: EAPC and CI upper limit <0.

Generalized additive models (GAM) characterized associations between burden indicators and Sociodemographic Index (SDI). All analyses used R v4.2.2 (*α* = 0.05), with incident cases/incidence rates reported as 95% UI and AAPC/EAPC as 95% CI.

## Results

### Global burden trends

#### Global trends in incidence

**Figure 1:**
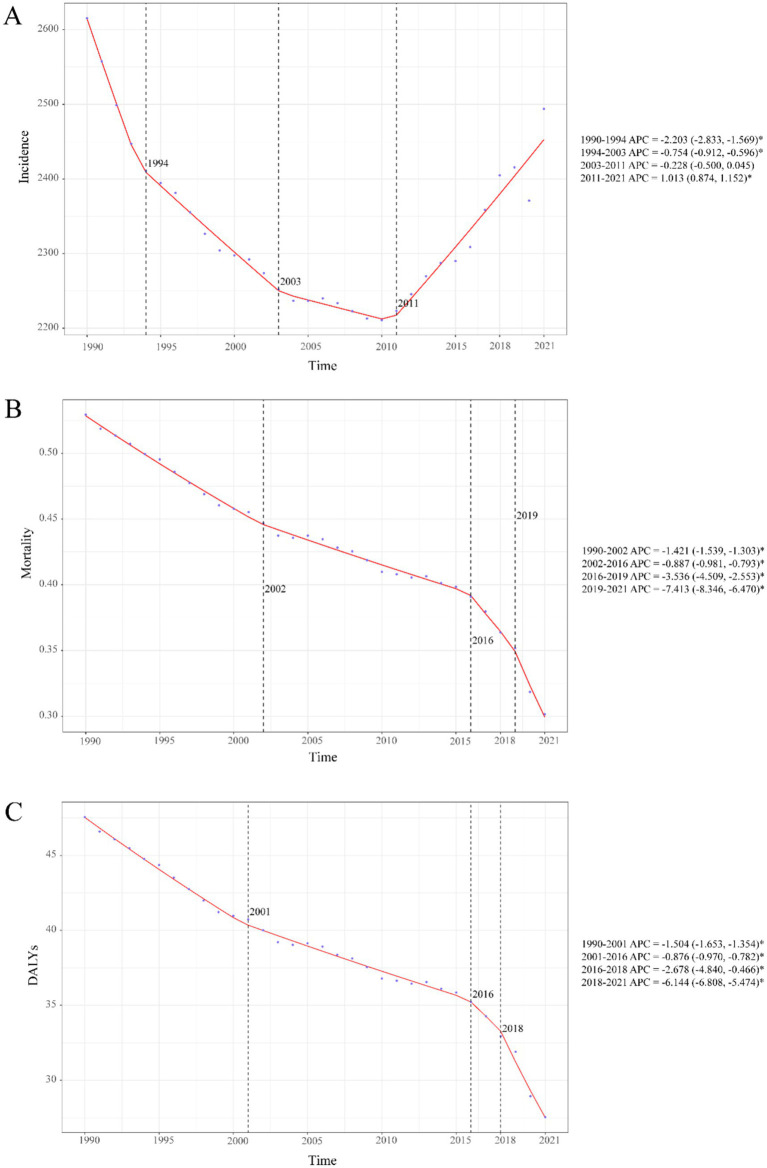
Temporal trends in childhood UTIs disease burden, 1990–2021. **(A)** Incidence rate. **(B)** Mortality rate. **(C)** DALY rate. Statistical significance was defined as *p* < 0.05 and indicated by an asterisk*.

**Figure 2:**
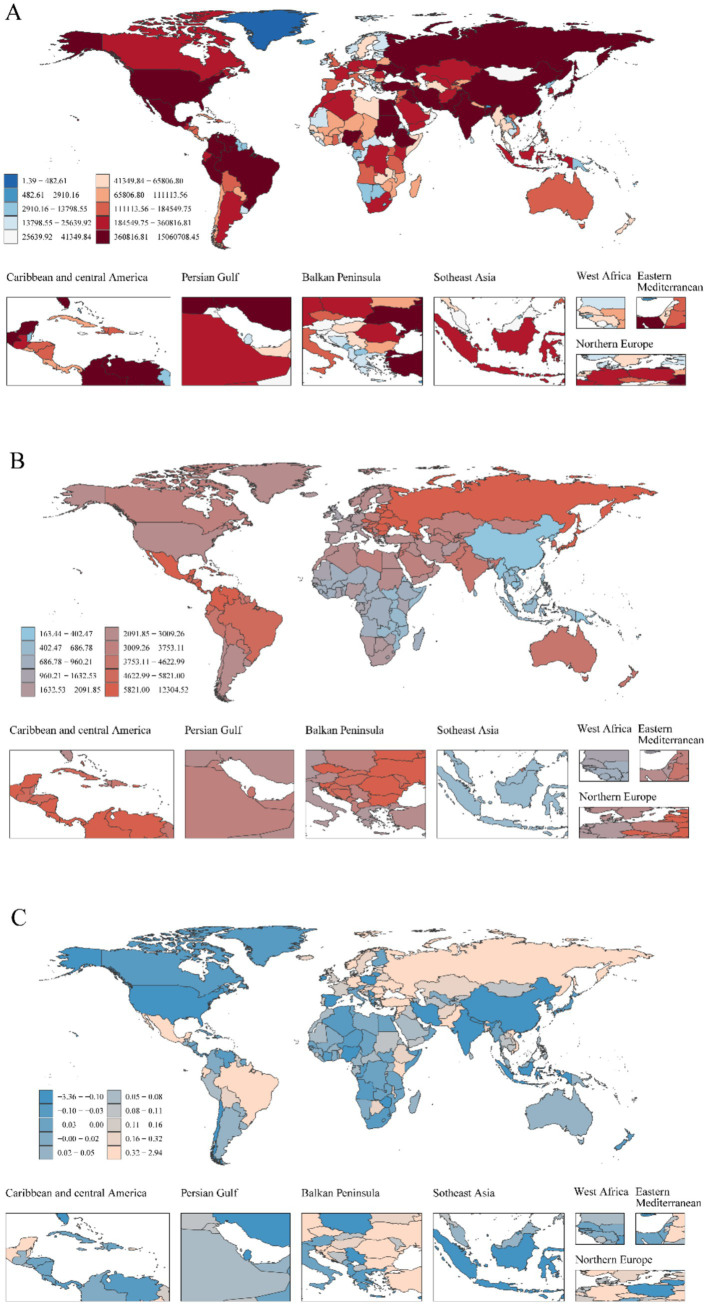
Global heterogeneity of childhood UTIs epidemiology: Incident cases, incidence rates, and EAPC (1990–2021). **(A)** Global distribution of incident case, with color intensity (light to dark red-brown) indicating higher burden. **(B)** Global distribution of incidence rate. **(C)** Global distribution of EAPC in incidence rate.

Childhood UTIs showed complex global trends from 1990 to 2021. While overall incidence rates declined (AAPC: −5.25%), a pivotal transition occurred post-2011, with rates shifting to a significant upward trajectory (APC: +1.01%; Figure 1A). This divergence is critical: although incidence rates decreased globally (EAPC: −0.17, 95% CI: −0.33–0.00; Figure 2C), absolute incident cases rose by 10.31% (Table 1). This paradox highlights persistent burden despite population-level improvements.

**Table 1:** Incidence, mortality and DALYs of UTIs in children of different sexes in all regions of the world between 1990 and 2021.

Indicator		Rate per 100,000 (95% UI)						
		1990		2021		1990–2021		
		Cases	Rate	Cases	Rate	Cases change %	Rate change %	EAPC*
Incidence	Male	13,332,728.63 (11,714,961.85, 15,677,839.10)	1,492.21 (1,311.14, 1,754.67)	14,231,936.01 (12,585,285.96, 15,922,091.77)	1,370.87 (1,212.26, 1,533.68)	6.74 (−3.19,11.00)	−8.13 (−16.68, −4.47)	−0.17 (−0.65, 0.32)
	Female	32,153,103.21 (28,175,042.13, 36,483,221.59)	3,802.13 (3,331.72, 4,314.17)	35,941,719.58 (31,555,455.40, 41,217,330.85)	3,691.27 (3,240.79, 4,233.08)	11.78 (7.41, 15.26)	−2.92 (−6.72, 0.10)	−0.17 (−0.23, −0.10)
	Both	45,485,831.83 (40,328,913.61, 51,549,924.40)	2,615.40 (2,318.88, 2,964.09)	50,173,655.59 (44,639,939.99, 56,681,228.97)	2,493.89 (2,218.84, 2,817.35)	10.31 (4.33, 13.82)	−4.65 (−9.82, −1.60)	−0.17 (−0.33, −0.00)
Mortality	Male	4,842.44 (3,040.83, 6,028.74)	0.54 (0.34, 0.67)	3,080.70 (2,204.76, 3,998.97)	0.30 (0.21, 0.39)	−36.38 (−52.41, 11.28)	−45.25 (−59.05, −4.23)	−1.47 (−1.63, −1.32)
	Female	4,364.11 (2,745.48, 5,723.65)	0.52 (0.32, 0.68)	2,985.16 (2,272.67, 3,662.55)	0.31 (0.23, 0.38)	−31.60 (−48.03, 20.83)	−40.59 (−54.86, 4.94)	−1.24 (−1.39, −1.09)
	Both	9,206.54 (6,746.00, 11,325.31)	0.53 (0.39, 0.65)	6,065.85 (4,661.36, 7,465.91)	0.30 (0.23, 0.37)	−34.11 (−47.75, −6.87)	−43.04 (−54.83, −19.50)	−1.36 (−1.51, −1.21)
DALYs	Male	429,908.40 (270,016.84, 535,129.14)	48.12 (30.22, 59.89)	276,087.90 (197,857.64, 356,034.63)	26.59 (19.06, 34.29)	−35.78 (−51.83, 11.14)	−44.73 (−58.54, −4.35)	−1.45 (−1.60, −1.30)
	Female	397,219.01 (256,970.28, 516,633.62)	46.97 (30.39, 61.09)	278,097.47 (214,989.79, 338,014.72)	28.56 (22.08, 34.71)	−29.99 (−46.23, 19.30)	−39.19 (−53.30, 3.61)	−1.19 (−1.33, −1.05)
	Both	827,127.41 (612,187.67, 1,014,914.76)	47.56 (35.20, 58.36)	554,185.36 (434,172.98, 676,905.68)	27.55 (21.58, 33.65)	−33.00 (−46.64, −6.52)	−42.08 (−53.87, −19.19)	−1.32 (−1.47, −1.18)

*EAPC is expressed as 95% CIs. EAPC, estimated annual percentage change; CI, confidence interval.

**Figure 3:**
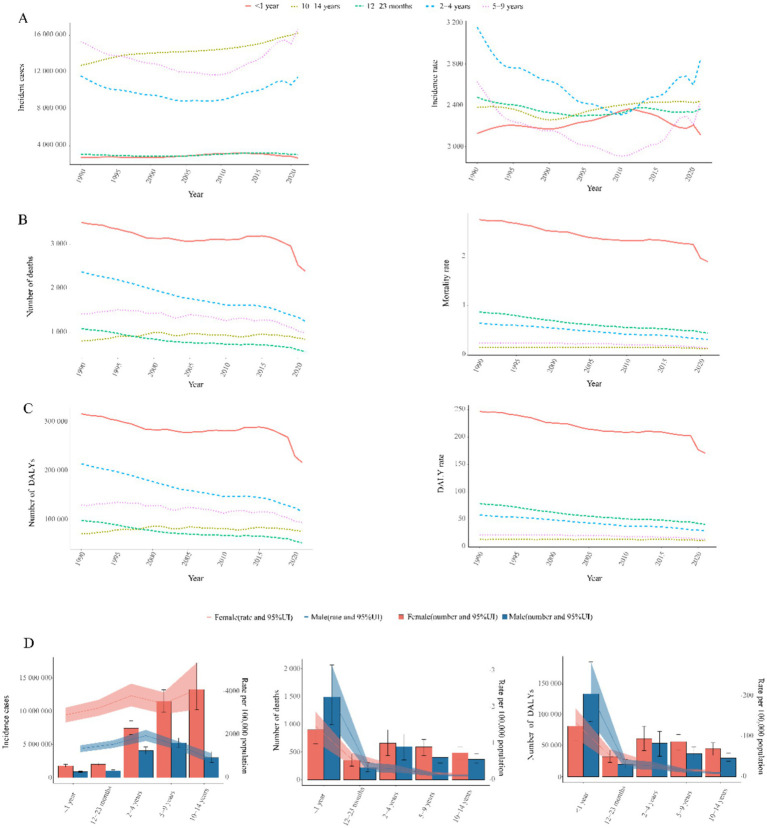
Age-sex stratified trends in childhood UTIs disease burden, 1990–2021. **(A)** Incidence burden: (Left) Temporal trends in incident cases by age group. (Right) Temporal trends in incidence rate by age group. **(B)** Mortality burden: (Left) Temporal trends in mortality cases by age group. (Right) Temporal trends in mortality rate by age group. **(C)** DALYs burden: (Left) Temporal trends in number of DALYs by age group. (Right) Temporal trends in DALY rates by age group. **(D)** Sex disparity: (Left) Incident cases and incidence rates. (Middle) Mortality cases and rates. (Right) Number of DALYs and DALY rates.

Global childhood UTIs incidence rates demonstrated a modest decline between 1990 and 2021, with a more pronounced reduction in boys than girls (Table 1). Despite this overall decrease, girls maintained a 2.7-fold higher incidence rate compared to boys in 2021—a disparity visually confirmed by the progressively increasing column heights from 0–4 to 10–14 years in Figure 3. Concurrently, boys showed peak incidence in early childhood (2–4 year group) before rates declined with age. This pattern aligns with global burden trends where incident cases increased 10.31% overall, despite a 4.65% decline in incidence rates (Table 1).

Notably, incidence rates declined consistently through 2010 for children aged 2–4 years and 5–9 years in Figure 3A, followed by a sustained upward trend thereafter. The 2–4 year cohort consistently exhibited the highest incidence burden across the study period. Conversely, the 5–9 year group demonstrated the lowest rates.

#### Global trends in mortality

Childhood UTI mortality showed consistent global declines (AAPC: −0.007%), accelerating sharply during 2019–2021 with a 7.4% annual drop— potentially linked to pandemic-era healthcare shifts (Figure 1B). By 2021, deaths had fallen 34.1% and mortality rates 43.0% since 1990 (Table 1), translating to about 3,100 fewer annual deaths. This decline was most pronounced in infants, who accounted for 64.8% of total deaths.

**Figure 4:**
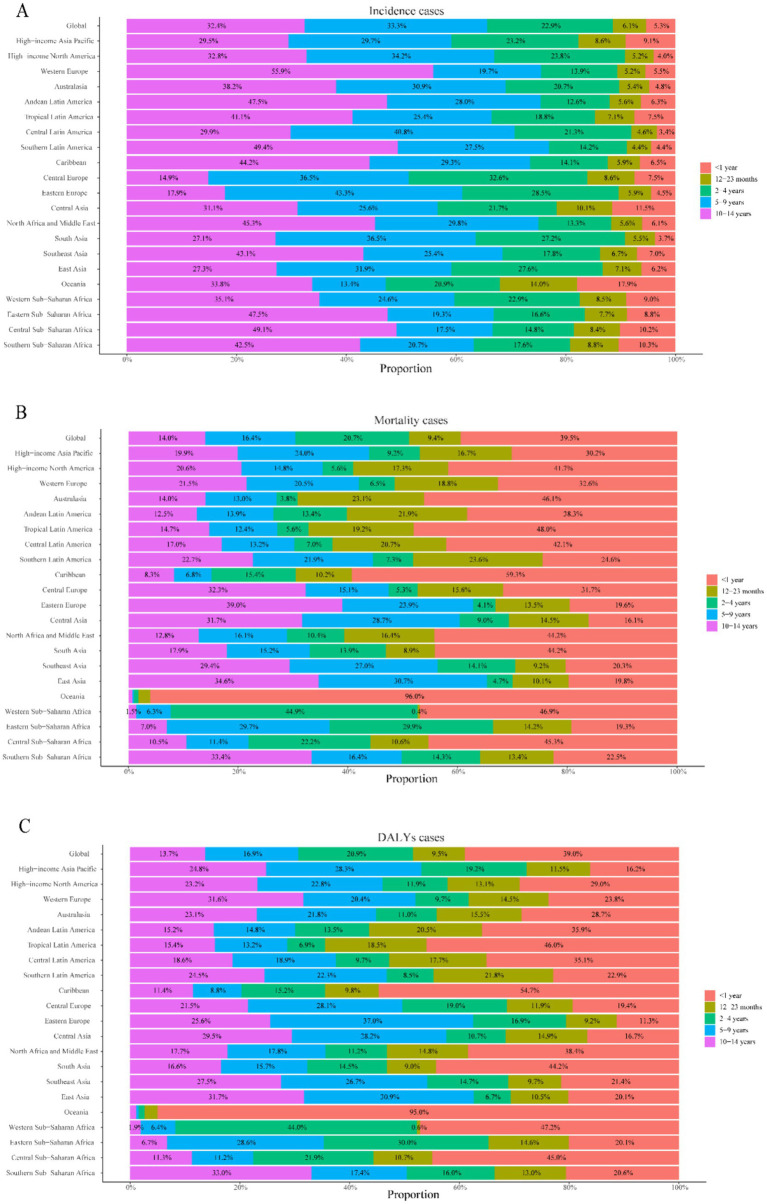
Age distribution of childhood UTIs disease burden by region, 2021. **(A)** Proportional attribution of incident cases. **(B)** Proportional attribution of mortality cases. **(C)** Proportional attribution of number of DALYs.

Notably, mortality reductions occurred across all age groups, with the steepest decline in 2–4 year olds (49.2%) and the smallest in 10–14 year olds (13.3%) (Figure 3B). Infants under 1 year (<1 year) consistently exhibited the highest mortality rates across all years (Figures 3B, 4B), accounting for 64.8% of total deaths in 2021. Boys in this age group showed 1.8-fold higher mortality than girls (*p* < 0.001, Figure 3D). Conversely, the 10–14 year group demonstrated the lowest mortality rates in all periods (Figure 3B), while the 5–9 year cohort had the second lowest rates. Gender disparities were age-dependent: girls aged 12–23 months showed marginally higher mortality than boys (*p* = 0.047), but no significant sex differences existed beyond age 2 years (all *p* > 0.05) (Figure 3D).

#### Global trends in DALYs

Childhood UTI-associated DALYs showed sustained declines (AAPC: −0.645%), with accelerated reduction during 2018–2021 (AAPC: −6.144%) (Figure 1C). Total DALYs decreased 33.0% from 1990 to 2021, while DALY rates fell 42.1% (Table 1), equivalent to about 273,000 fewer DALYs lost annually.

Infants <1 year consistently bore the highest burden, accounting for 64.3% of total DALYs in 2021: fold higher than the 10–14 year group (lowest burden cohort). Universal reductions occurred across age strata, with maximal decline in 2–4 year olds (50.5%) and minimal in 10–14 year olds (13.9%) (Figures 3C, 4C).

Gender disparities: Boys <1 year showed 1.8-fold higher DALYs than girls. No significant sex differences beyond age 2 years (Figure 3D).

#### Childhood UTIs: SDI regional trends

**Figure 5:**
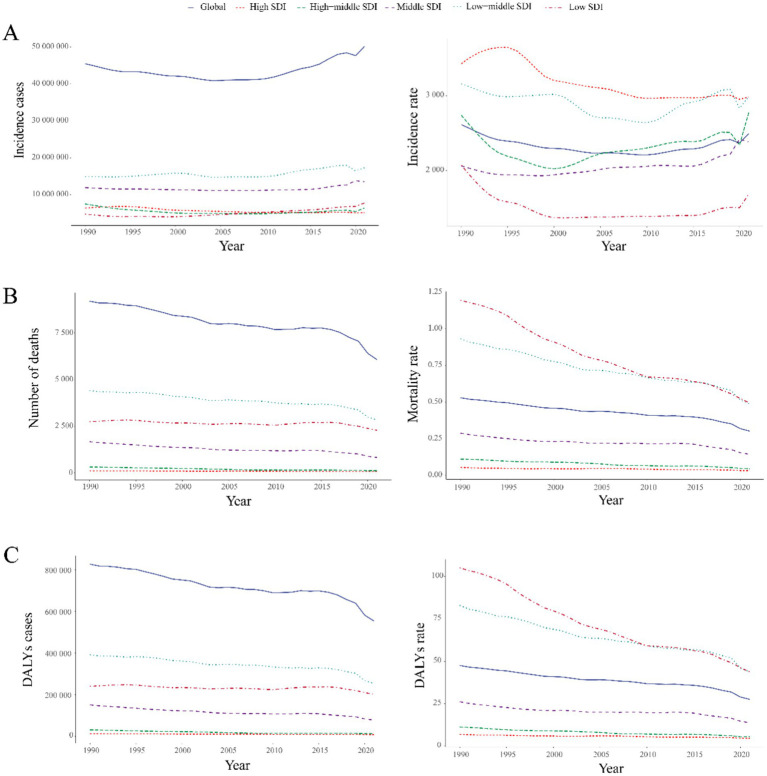
Temporal trends in global UTIs burden by SDI quintile, 1990–2021. **(A)** Incident cases and incidence rate. **(B)** Mortality cases and rate. **(C)** Number of DALYs and DALY rates.

Between 1990 and 2021, low-middle SDI regions exhibited the highest global burden of childhood UTIs, with the most severe incidence, mortality, and DALY rates across all metrics (Supplementary Tables 1–3). Low, low-middle, and middle SDI regions displayed increasing incidence trends, while high-middle and high SDI regions showed significant declines (Figure 5A).

**Figure 6:**
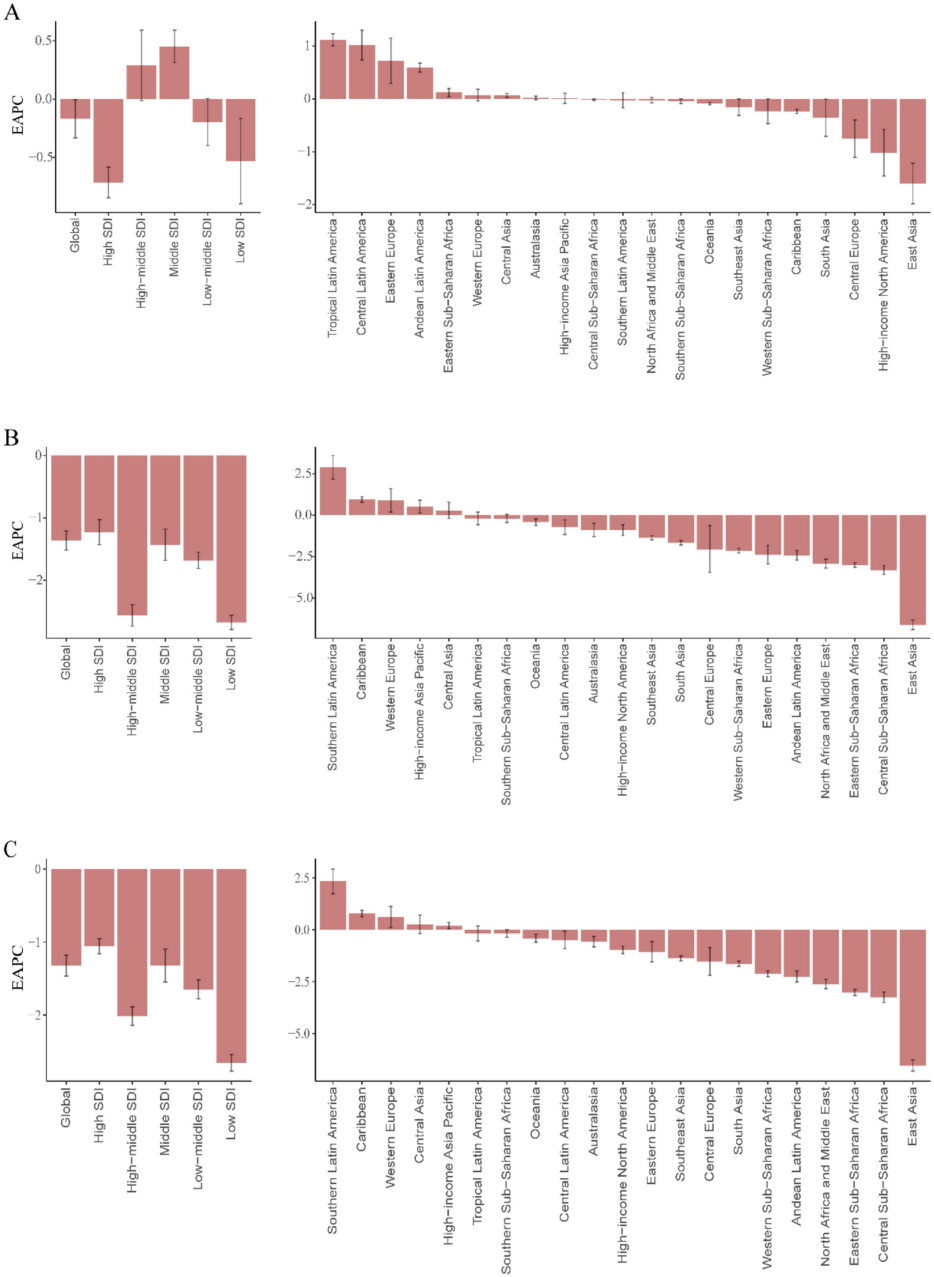
EAPC in childhood UTIs burden metrics by development level and region, 1990–2021. **(A)** EAPC of incidence rate. **(B)** EAPC of mortality rate. **(C)** EAPC of DALY rate.

The most substantial increase occurred in low-SDI regions (63.43%), contrasting markedly with the sharpest decline in high-SDI regions (19.14%). This divergence was particularly evident in country-level comparisons within Supplementary Table 1, where low-SDI nations such as Afghanistan and Nigeria demonstrated considerably lower actual incidence rates than middle-SDI countries like India (Figure 6A).

Global mortality and DALYs decreased substantially during the study period. The most pronounced reductions occurred in high-middle SDI regions (65.84 and 58.19%, respectively). High-SDI regions maintained the lowest absolute values for both indicators (Figures 5B,C, 6B,C). Low-SDI regions exhibited the most rapid declines in mortality and DALY rates (lowest EAPC values), whereas high-SDI regions showed the smallest magnitude of reduction (Figure 6).

**Figure 7:**
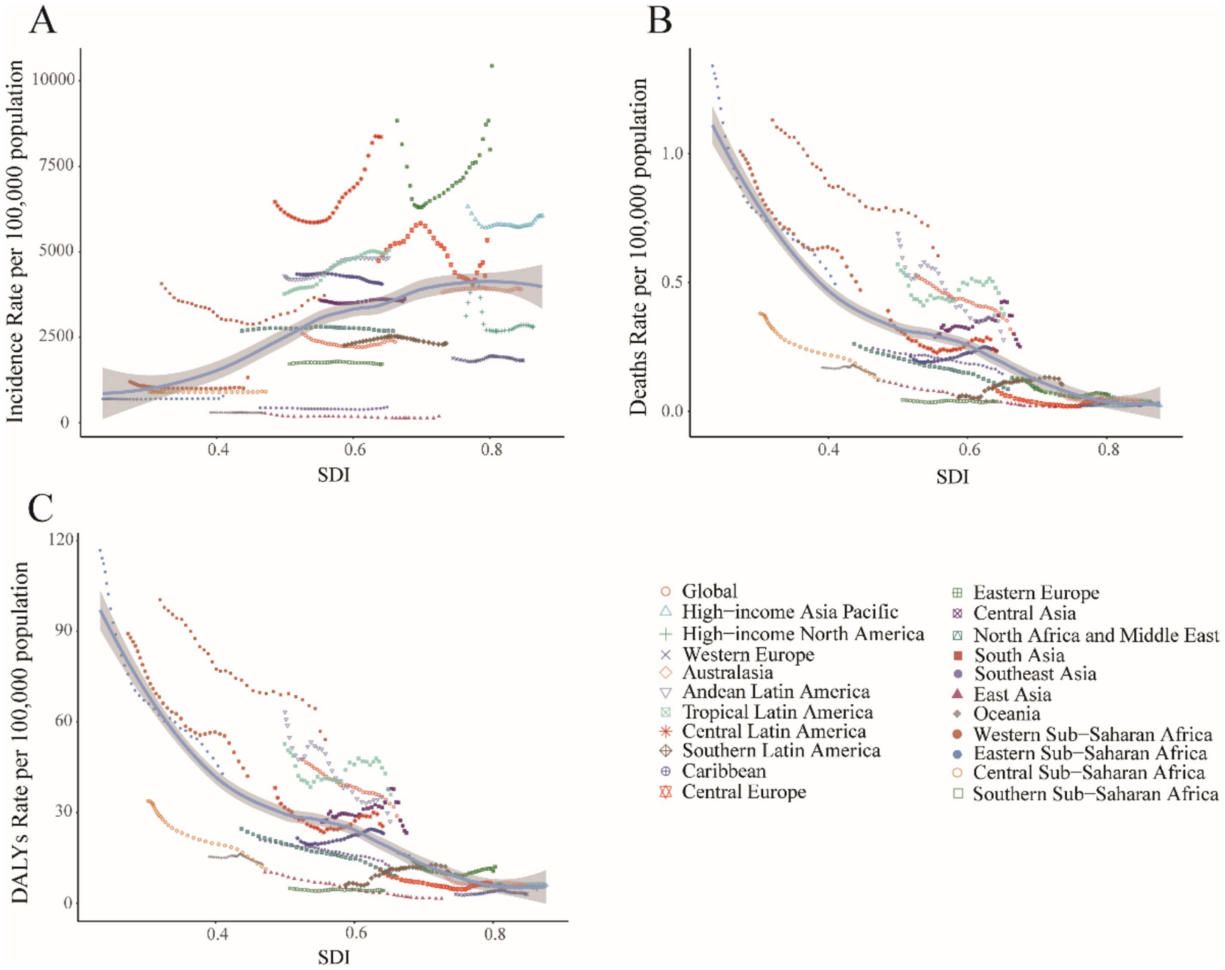
Association between childhood UTIs burden and regional SDI, 1990–2021. **(A)** Incidence rate. **(B)** Mortality rate. **(C)** DALY rate.

In 2021, high-SDI and low-middle-SDI regions reported incidence rates exceeding the global average, while low-SDI regions demonstrated the lowest incidence (Figure 7A). This distribution contrasted sharply with mortality and DALY rate patterns: low and low-middle SDI regions exhibited mortality and DALY rates substantially higher than global means (Figures 7B,C). Further analysis revealed distinct trends by development level: incidence rates increased with rising SDI, peaking in middle-SDI regions (EAPC 0.45, 95% CI: 0.31–0.59%) and reaching a minimum in high-SDI regions (EAPC −0.71, 95% CI: −0.85 to −0.58%). Conversely, mortality and DALY rates decreased with higher SDI levels (Supplementary Table 1).

Middle-SDI countries in Latin America and the Caribbean displayed persistently high incidence rates (Figure 6A), whereas low-income regions such as sub-Saharan Africa showed lower but more volatile incidence patterns (Figure 7A). South Asia consistently reported the highest absolute case numbers (29.7% of global cases in 2021), while Oceania recorded the lowest burden. East Asia maintained the world’s lowest incidence rates (Figure 6A). Tropical Latin America showed the most rapid incidence growth (EAPC 1.12, 95% CI: 1.01–1.23%), contrasting with the steepest decline in High-Income North America (EAPC −1.02, 95% CI: −1.46 to −0.57%) (Supplementary Table 1).

High-income regions demonstrated stable, low mortality and DALY rates (Figures 6B,C). However, certain middle-income areas such as Andean Latin America exhibited increasing trends. South Asia accounted for the highest mortality and DALY counts in 2021 (32.1% of global totals), while Australia reported the lowest burden. Western Europe experienced the greatest mortality increase (EAPC 0.89, 95% CI: 0.18–1.60%), whereas East Asia achieved the most substantial reductions (EAPC −6.62, 95% CI: −6.90 to −6.33%; EAPC −6.54, 95% CI: −6.81 to −6.28%).

### Childhood UTIs: national trends

#### National trends in incidence

National disparities revealed critical contrasts in 2021 (Figures 2A,B, 6A). India carried the highest absolute burden of childhood UTIs cases globally, while Ukraine demonstrated the most severe incidence intensity (Supplementary Table 4). Temporal trends highlighted divergent trajectories: Ecuador showed the sharpest acceleration in incidence (EAPC: 1.79%; 95% CI, 1.47–2.10%), whereas Poland had the steepest decline (EAPC: −3.33%; 95% CI, −4.14–4.51%). GBD analysis indicates that most Latin American regions demonstrated a continuing upward trend in incidence rates, whereas East Asia showed a consistent downward trend with the most substantial annual decline (Figure 6A). In 2021, the global incidence rate of UTIs was 2,493.89 per 100,000 population (95% UI: 2,218.84–2,817.35), which exceeded the rates in 34 countries but remained lower than those in 170 countries.

#### National trends in mortality

National mortality disparities revealed critical extremes in 2021 (Supplementary Figures 1A,B; Figure 6B). India bore the highest absolute death toll from childhood UTIs, while Pakistan exhibited the most severe mortality intensity (Supplementary Table 5). Temporal trends showed highly polarized trajectories. Kuwait experienced the sharpest mortality acceleration (EAPC 5.74%; 95% CI: 3.94–7.57%). This contrasted sharply with China, which achieved the most dramatic decline (EAPC -6.95%; 95% CI: −7.26 to −6.64%). These national patterns mirrored broader regional trends, with concerning rises in South Latin America and unprecedented reductions in East Asia (Figure 6B). In 2021, the global UTIs mortality rate stood at 0.30 per 100,000 population (95% UI: 0.23–0.37). This global average was exceeded by 177 countries, while only 27 countries fell below it.

#### National trends in DALYs

In 2021, India reported the highest absolute burden of childhood UTIs, with DALYs reaching 160,543.21 (95% UI: 98,414.73–243,629.54). Pakistan recorded the world’s highest age-standardized DALY rate, at 107.86 per 100,000 population (95% UI: 80.45–146.28) (Supplementary Table 6; Supplementary Figures 2A,B). From 1990 to 2021, Mauritius experienced the most pronounced increase in DALY rate (EAPC: 4.11%; 95% CI: 3.52–4.70), while China demonstrated the largest decline (EAPC: −6.86%; 95% CI: −7.16 to −6.56) (Supplementary Table 6; Supplementary Figure 2C). The global childhood UTIs DALY rates was 27.55 per 100,000 population (95% UI: 21.58–33.65) in 2021. This rate was higher than that of 164 countries and territories but lower than that of 40 others.

### Frontier analysis

This analysis examines the potential for reducing the incidence, mortality, and DALY rates of UTIs among children relative to a country’s developmental status, as measured by the SDI, based on data from 1990 to 2021.

#### Frontier analysis in incidence

**Figure 8:**
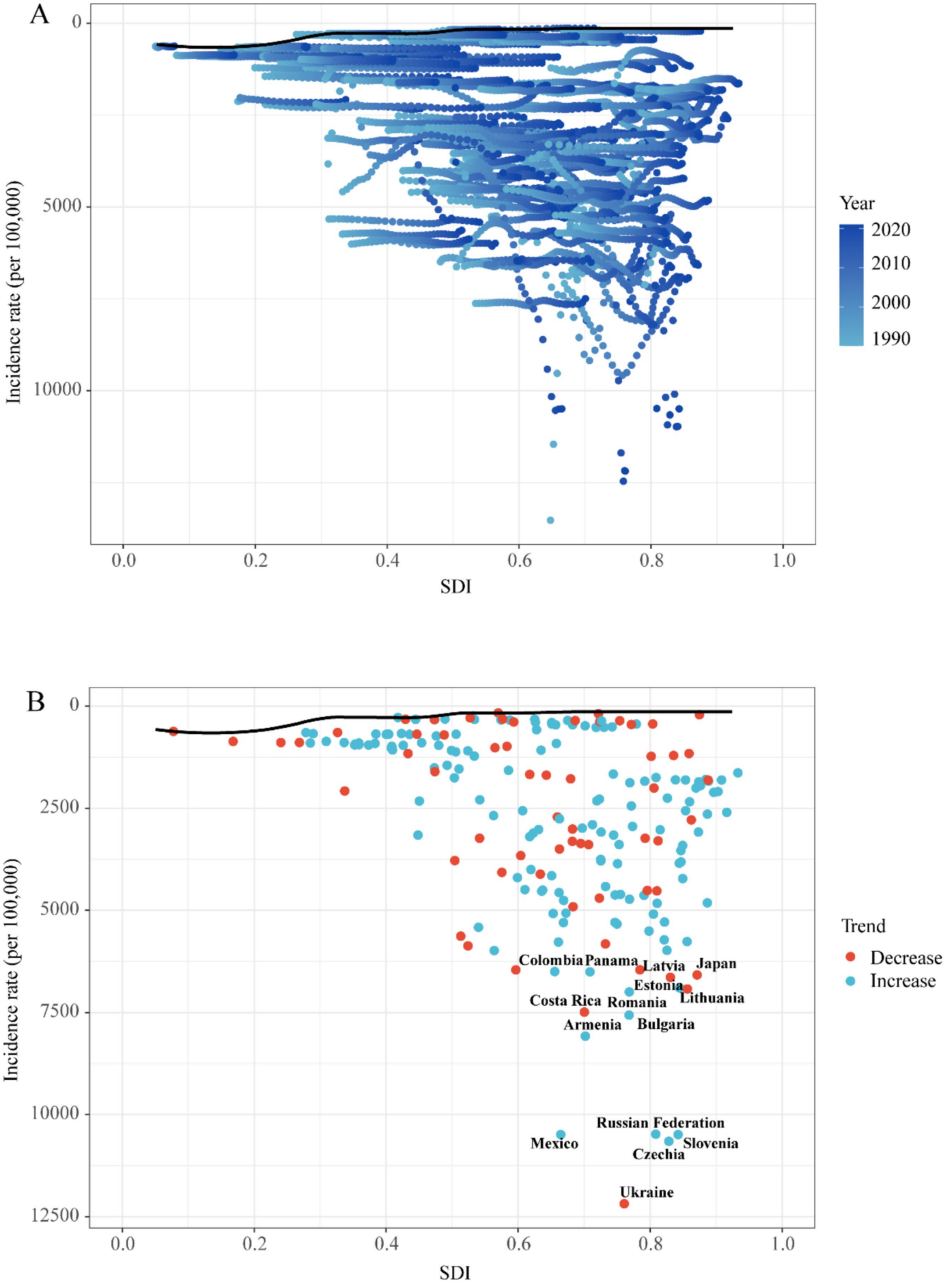
Frontier analysis exploring the relationship between SDI and incidence rate for 5 childhood UTIs in 204 countries and territories. **(A)** Temporal Patterns of SDI and Incidence from 1990 to 2020. **(B)** National Trends and Outliers in Incidence-SDI Association.

High-SDI underperformers (e.g., Ukraine, Czechia, Russia; SDI > 0.8) showed the largest gaps between observed and potential outcomes. This indicates that greater socioeconomic resources do not ensure optimal UTIs management. Conversely, low-SDI exemplars like Ethiopia and Liberia (SDI < 0.5) achieved high efficiency despite resource constraints. Two cohorts demonstrated exceptional performance: Resource-limited optimizers (e.g., North Korea, Somalia) maximized outcomes within developmental constraints; Development-defying underperformers (e.g., Ukraine, Japan) revealed systemic inefficiencies. This bifurcation demonstrates that while national wealth is broadly correlated with a reduced disease burden, health system efficiency is an independent factor. This is exemplified by the contrasting outcomes of low-SDI overachievers and high-SDI underperformers (Figure 8; Supplementary Table 6).

#### Frontier analysis in mortality

Low-SDI countries like Zimbabwe and Rwanda (SDI < 0.5) achieved exceptional mortality management despite resource constraints, while high-SDI nations such as Qatar and Belgium (SDI > 0.8) exhibited significant outcome gaps—Qatar’s mortality gap was 3.2-fold wider than Rwanda’s. Efficiency pioneers (e.g., Taiwan, Germany) attained near-optimal outcomes across development spectra, contrasting with development-defying laggards (e.g., Uganda, India) (Supplementary Figure 4; Supplementary Table 7).

#### Frontier analysis in DALYs

Low-SDI exemplars like Somalia (SDI = 0.27) and Niger (SDI = 0.35) achieved exceptional burden management—outperforming 78% of high-SDI nations—while high-SDI underperformers exemplified by Monaco (SDI = 0.94) and Norway (SDI = 0.93) exhibited significant outcome gaps despite maximal resources. Critical insights emerged: Somalia’s DALY gap was 2.3-fold narrower than Monaco’s; Resource curse phenomenon: 60% of high-SDI countries showed gaps exceeding low-SDI leaders; Global outliers: Vietnam (mid-SDI) and North Korea (low-SDI) joined efficiency pioneers. This demonstrates that optimal health outcomes require system efficiency beyond economic development (Supplementary Figure 5; Supplementary Table 8).

## Discussion

UTIs represent a prevalent bacterial infection among children (11–13). In 2021, global surveillance data identified 50.17 million incident cases of childhood UTIs, corresponding to an age-standardized incidence rate of 2,493.89 per 100,000 person-years (Table 1). Diagnostic challenges—including nonspecific symptoms and limited urine culture access—frequently result in underreporting (14). This surveillance gap aligns with documented patterns in resource-limited settings (5, 6), where compromised diagnostic capacity may obscure true disease prevalence. UTIs are a major global health concern that significantly impacts children’s physical and mental health (15). These infections can lead to serious complications, including sepsis, kidney abscesses, and acute kidney injury (16). Additionally, UTIs can cause chronic renal damage characterized by dysplasia, tubular dysfunctions, and permanent scarring (17–20). Approximately 8% of children experience at least one UTI between 1 month and 11 years of age (12, 13). Up to 30% may have a recurrence within 6–12 months of their first infection (21, 22). UTIs incur substantial medical and societal costs (23–26). In the U.S., they account for approximately 2.9 million emergency visits and 3.5 million outpatient visits annually (27). Hospitalization costs average 13,000 in the U.S. and €5,700 in Europe (28), with total U.S. expenditures exceeding 6 billion per year (29). Our findings on rising incident cases in low-SDI regions parallel child pneumonia trends in sub-Saharan Africa (30), reflecting system-level constraints in primary care infrastructure—a key theme in health systems literature on transitions in infectious disease epidemiology (31).

Previous epidemiological studies have primarily focused on specific countries or regions. In contrast, He et al. (32) analyzed global trends in UTIs burden from 1990 to 2021 using GBD data and projected future trajectories. However, comprehensive epidemiological statistics on pediatric UTIs remain limited worldwide. To address this gap, our study is the first to utilize GBD data (1990–2021) to systematically evaluate the incidence, mortality, and DALYs of childhood UTIs, stratified by region, country, SDI, sex, and age group. These findings provide an evidence base to inform targeted prevention strategies and public health policy development.

Our analysis revealed significant sex-based disparities in pediatric UTIs burden. Consistent with existing literature (2), females exhibited higher incidence rates than males. As shown in Table 1 and Figure 3, the 2021 incidence rate was 1,370.87 per 100,000 in males compared to 3,691.27 per 100,000 in females—exceeding the global average. This divergence is primarily attributable to anatomical and physiological factors (2). Females have shorter urethras with closer proximity to the anus, facilitating bacterial entry; suboptimal hygiene practices (e.g., improper wiping) increase risk, evidenced by meta-analyses showing 15–20% infection’s diseases reduction through Water Supply, Sanitation and Hygiene (WASH) interventions in LMICs (33). In males, UTIs frequently associate with congenital anomalies like vesicoureteral reflux (VUR), though these are comparatively rare (1, 34). Age-stratified patterns further elucidate this disparity. Infants show comparable infection risk across sexes, but female incidence increases progressively with age (2). Male incidence peaks in 0–4 year-olds before declining, widening the sex gap. Uncircumcised status elevates bacterial colonization risk (28), while rising circumcision rates with age reduce UTIs (35). Contributing biological factors include: higher diabetes incidence in females aged 5–14 years (GBD database); Footnote 1 (36, 37); puberty-related physiological changes (e.g., Menstrual cycle-associated risk elevation and estrogen-mediated urethral/bladder function alterations) (38, 39), female-focused hygiene education remains essential for primary prevention; male infants warrant heightened diagnostic vigilance due to atypical presentations, early identification of anatomical abnormalities in males reduces complication risks. The observed diagnostic challenges in low-SDI settings further emphasize the need for context-specific management protocols aligned with healthcare infrastructure capabilities (34, 38).

The highest incidence of UTIs occurs during preschool years, with females exhibiting significantly higher rates than males, as demonstrated in Figures 3D, 4A. This sex-based disparity is attributable to physiological differences and behavioral factors (40–42). Globally, while the overall incidence of pediatric UTIs has declined over time, the absolute number of cases increased from 1990 to 2021 (Table 1; Figure 1A). This pattern aligns with general UTIs epidemiology but contrasts with global population incidence trends (5, 32), potentially reflecting population growth and aging dynamics (43, 44). The historical decline may be associated with healthcare advancements and improved disease awareness, yet incidence rates have risen over the past decade (Figure 1A). Age-stratified analysis reveals this increase is particularly pronounced among children aged 2–9 years (Figure 3A). Contributing factors include the emergence of multidrug-resistant pathogens, notably extended-spectrum *β*-lactamase (ESBL)-producing *Escherichia coli* and Klebsiella species in pediatric UTIs (45, 46). Antimicrobial resistance (AMR) represents a key driver of this resurgence, with studies indicating a 30–50% increase in AMR prevalence among pediatric UTIs in middle-SDI regions since 2010, correlating with antibiotic misuse patterns in countries including Saudi Arabia and Brazil (45, 47). For instance, ESBL-producing *E. coli* now accounts for >40% of pediatric UTI isolates in the Middle East (47), reducing therapeutic efficacy and elevating complication risks (27, 45). The burden of AMR disproportionately affects low- and middle-SDI regions, primarily due to weaker antimicrobial stewardship. This is starkly illustrated in South Asia, where multidrug-resistant infections contribute to approximately 20% higher DALY rates (Supplementary Table 6) (46, 48). Addressing AMR requires Sustainable Development Goals (SDG)-aligned strategies such as the WHO Global Action Plan on AMR to mitigate treatment failures and resource inefficiencies (28, 45).

This trend has contributed to the increased number of cases among children. In specific Middle Eastern nations including Saudi Arabia, Iran, and Egypt, antibiotic misuse and overuse represent documented public health threats that exacerbate antimicrobial resistance (45, 47–51). Concurrently, rising childhood obesity and metabolic syndrome may elevate UTIs susceptibility through metabolic dysfunctions that compromise immune responses (52). Epidemiologically, South Asia bears the highest overall burden of childhood UTIs, leading in incident cases, mortality, and DALYs. Conversely, Latin America exhibits the world’s most rapidly accelerating incidence growth rate. This regional pattern supports existing evidence that pediatric UTIs demonstrate enhanced transmissibility in tropical climates (53, 54). Consequently, high and upper-middle SDI regions should enforce evidence-based antibiotic stewardship programs to curb resistance development. Tropical countries require urgent implementation of interventions including: enhanced infectious disease surveillance, public health education campaigns, expanded screening with early treatment protocols, and WASH infrastructure improvements to mitigate pediatric UTIs burden.

These regional patterns are directly linked to SDG progress. Empirical evidence reveals clear SDG-linked burden dynamics. For instance, India’s sanitation deficits (SDG 6) correlate with its disproportionately high share (38.7%) of global childhood UTI-related DALYs, with urban slums being particularly affected due to population density amplifying transmission risk (53). Frontier efficiency analysis quantifies these systemic disparities. Somalia, a low-SDI nation, outperformed 78% of high-SDI countries. This demonstrates that health system optimization is paramount for achieving SDG 3—even in settings with severe resource constraints (Figure 8).

Disparities in diagnostic capacity critically influence global burden distributions. This is evidenced by elevated DALY rates in low-SDI regions (Figure 7) and pronounced health system efficiency gaps in under-resourced settings (Figure 8). Significant geographic variations in pediatric UTIs rates were observed. East Asia demonstrated lower incidence rates, while North America, South America, Russia, Eastern Europe, and parts of Asia and the Middle East exhibited higher rates. These patterns correlate with global population density distributions (Supplementary Table 1; Figure 6). Notably, China and Southeast Asian nations report lower incidence despite China’s demographic magnitude (Figure 2A; Supplementary Table 4), reflecting documented advancements in UTIs diagnosis, treatment, and prevention. In contrast, Russia, Eastern Europe, and selected Latin American and Middle Eastern countries exhibit elevated incident cases and incidence rates (Figure 2B; Supplementary Table 4). This pattern is potentially attributable to disparities in healthcare system development, antibiotic prescribing practices, and socioeconomic factors. Region-specific drivers include: catheter-associated UTIs with antibiotic resistance in Russia/Eastern Europe, and culturally mediated epidemiological patterns in Mexico/South America (55). Collectively, these findings align with social determinants of health theory (56), warranting comprehensive investigation of multidrug resistance as a strategic approach to reducing global UTI burden. Anomalously, South Latin America demonstrated a declining EAPC for incidence but substantially increasing EAPC for mortality and DALYs (Figure 6). This divergent pattern, potentially influenced by data limitations, merits targeted investigation. Policy priorities include disseminating health information, enhancing public awareness, and implementing early interventions to reduce mortality and complications.

Our analysis indicates higher reported UTIs incidence rates in high-SDI regions, which likely reflects enhanced detection capabilities within advanced healthcare systems rather than true epidemiological differences (57, 58). Conversely, substantial underreporting in low-SDI regions is attributed to constrained resources and surveillance limitations. Despite these data limitations, these regions exhibited the most pronounced declines in pediatric UTIs burden—a trend potentially attributable to successful public health initiatives. Middle-SDI regions exhibited the steepest increase in incidence among all SDI categories. This pattern may indicate a developmental mismatch, where rapid economic growth has outpaced the development of healthcare infrastructure. Furthermore, rapid urbanization in these regions may strain sanitation systems and amplify transmission risks in densely populated areas. Consequently, middle- and low-SDI countries merit prioritization of coordinated health-economic investments, directing increased fiscal allocations toward public health systems to mitigate UTIs burden.

Childhood UTI-associated mortality and DALYs remained relatively low overall (Table 1). However, the burden was predominantly concentrated in children under 1 year of age, with higher rates observed in males than in females. This pattern is potentially attributable to immunological immaturity during infancy (28, 39). Additionally, male infants demonstrate increased susceptibility to severe complications such as pyelonephritis following UTIs onset (59, 60).

This study is the first to apply frontier efficiency analysis to evaluate improvement potential for childhood UTIs across 204 countries and territories. Comprehensive data from this analysis are provided in the Supplementary Table 9. Notably, certain low-SDI nations (e.g., Somalia, Niger) demonstrate effective disease control relative to resource constraints. Conversely, several high-SDI regions exhibit suboptimal burden management despite lower absolute DALYs and morbidity than low-SDI counterparts, performing substantially below efficiency frontiers. These findings indicate that enhanced resource allocation strategies and evidence-based interventions remain imperative for optimizing outcomes in high-resource settings.

This study underscores the necessity of region-specific public health interventions for childhood UTIs. Low-SDI settings require prioritized strengthening of healthcare systems and improvements in medical resource accessibility, integrated with targeted prevention initiatives to reduce the burden. Concurrently, high-SDI regions require resource optimization and health disparity mitigation to advance burden reduction. Comprehensive standardized surveillance systems are particularly critical in resource-limited countries to elucidate epidemiological patterns and facilitate evidence-based control strategies.

Several limitations warrant consideration when interpreting these findings. The aggregation of urinary tract infections with interstitial nephritis in GBD data may introduce analytical inaccuracies. Additionally, heterogeneous data collection methodologies across healthcare systems could propagate measurement bias. This issue is particularly pronounced in under-resourced settings, where underreporting and documentation gaps persist, potentially compromising data validity for middle- and low-SDI regions. These operational constraints restrict investigations of risk factors and preclude stratification of UTI subtypes within the GBD framework. Consequently, they limit granular analyses of therapeutic outcomes and intervention strategies.

Future directions include enhanced global antimicrobial resistance surveillance with specific emphasis on pediatric antibiotic stewardship. Dissemination of evidence-based guidelines is needed to elevate clinical and public prioritization of childhood UTIs. Governments should implement targeted parental education programs on evidence-based infant care practices. Concurrently, educational institutions must incorporate hygiene education and nutritional support initiatives, particularly in resource-limited settings. Healthcare systems worldwide must dedicate efforts to: (1) advancing pediatric-specific diagnostic tools, (2) establishing longitudinal cohorts for long-term outcome assessment, and (3) integrating UTIs management into child health priority frameworks.

## Conclusion

This study documents an overall decline in global childhood UTIs incidence from 1990 to 2021, which contrasts with a significant resurgence observed over the past decade. The persistently high annual incident cases count represents an ongoing public health challenge. Substantial epidemiological heterogeneity exists across demographic strata—notably the disproportionately elevated burden in South Asia and consistently higher incidence among females versus males, while infant mortality and DALYs demonstrate male predominance. These patterns necessitate evidence-based, population-specific intervention strategies. Methodologically, inherent limitations in GBD data aggregation may obscure true epidemiological profiles. Enhanced primary data collection in future iterations will be crucial to mitigate these constraints.

## Data Availability

The datasets presented in this study can be found in online repositories. The names of the repository/repositories and accession number(s) can be found at: https://vizhub.healthdata.org/gbd-results/.

## Footnotes

^1^ https://vizhub.healthdata.org/gbd-results/
