# Supplementary material for: Global trends in childhood urinary tract infections, 1990–2021: results from the GBD study
Source: Front Public Health. 2025 Sep 25;13:1593206. doi: 10.3389/fpubh.2025.1593206 (PMC12507804; doi:10.3389/fpubh.2025.1593206)
Supplement: Supplementary file 1 [file Supplementary_file_1.docx]

**Supplementary Fig. 1:** Global Heterogeneity of childhood UTIs Epidemiology: Mortality cases, Mortality rates, and EAPC (1990–2021). A, Global distribution of mortality cases, with color intensity (light to dark red-brown) indicating higher burden. B, Global distribution of mortality rate. C, Global distribution of EAPC in mortality rate.

**Supplementary Fig. 2:** Global Heterogeneity of childhood UTIs Epidemiology: Number of DALYs, DALYs rates, and EAPC (1990–2021). A, Global distribution of number of DALYs, with color intensity (light to dark red-brown) indicating higher burden. B, Global distribution of DALYs rate. C, Global distribution of EAPC in DALYs rate.

**Supplementary Fig. 3:** Age Distribution of childhood UTIs Disease Burden by Region, 2021. A, Proportional attribution of incidence rate. B, Proportional attribution of mortality rate. C, Proportional attribution of DALYs rate.

**Supplementary Fig.4:**Frontier analysis exploring the relationship between SDI and mortality rate for childhood UTIs in 204 countries and territories.

**Supplementary Fig.5:**Frontier analysis exploring the relationship between SDI and DALYs rate for childhood UTIs in 204 countries and territories.


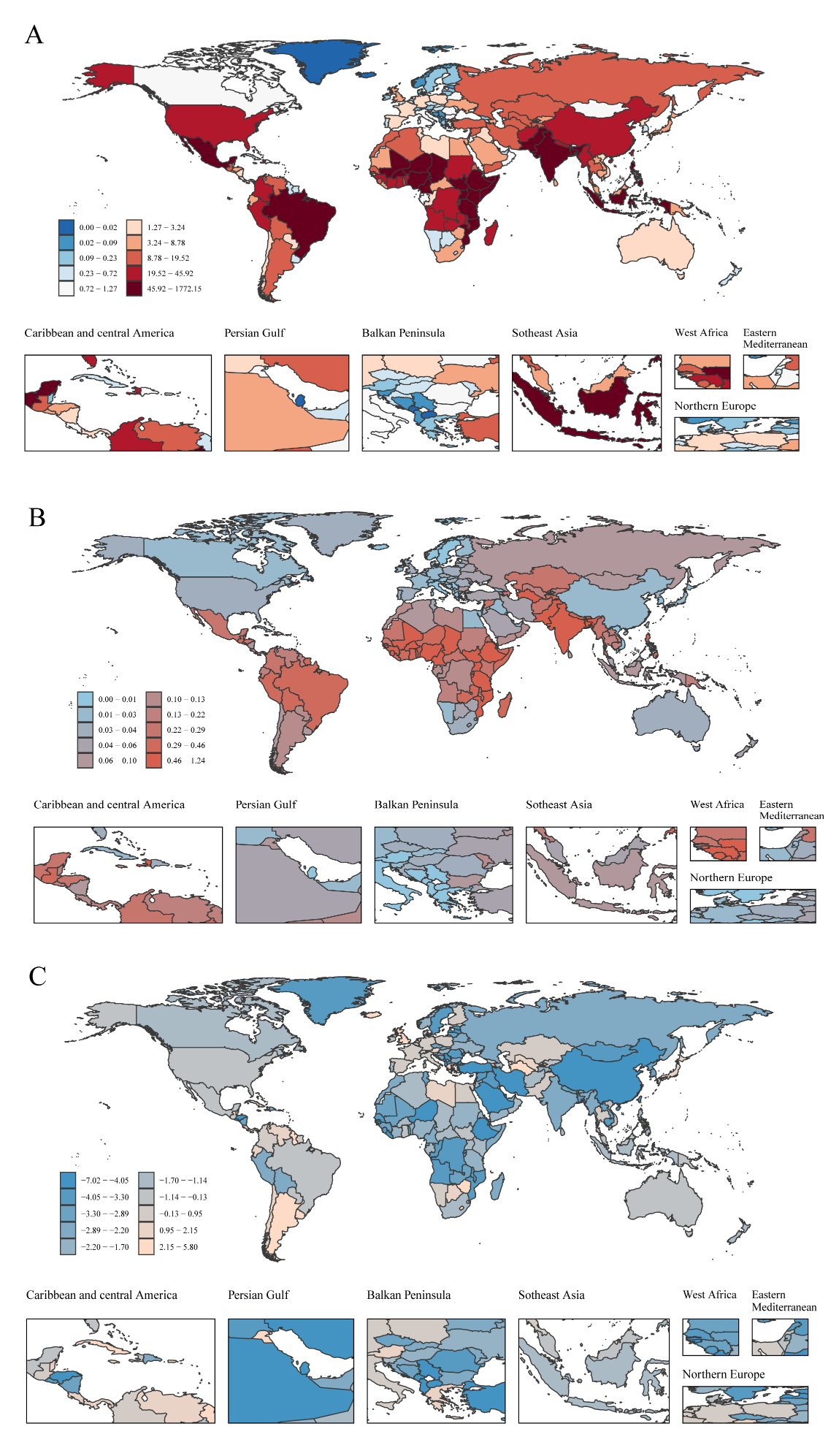


**Supplementary Fig. 1:** Global Heterogeneity of childhood UTIs Epidemiology: Mortality cases, Mortality rates, and EAPC (1990–2021). A, Global distribution of mortality cases, with color intensity (light to dark red-brown) indicating higher burden. B, Global distribution of mortality rate. C, Global distribution of EAPC in mortality rate.


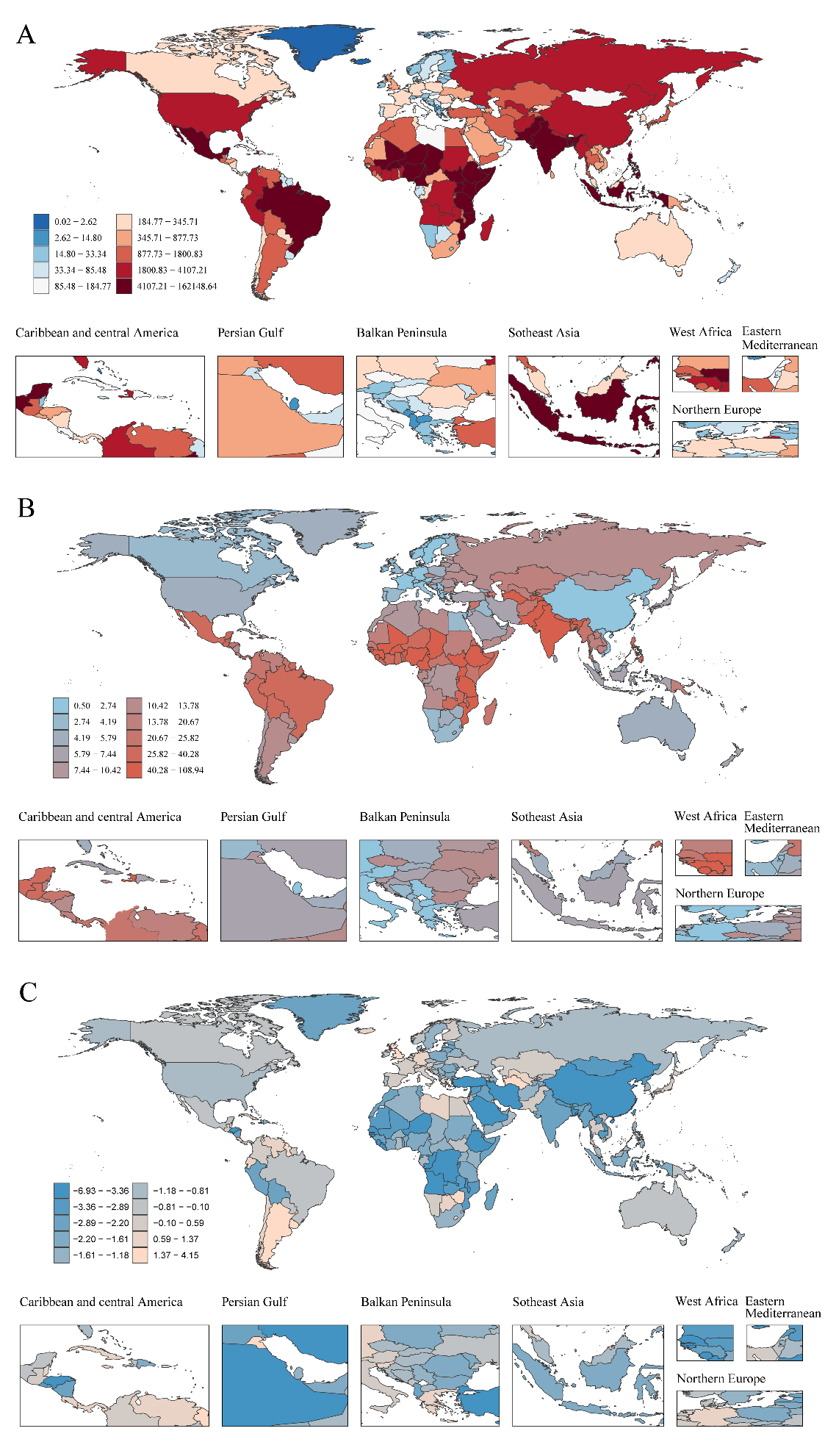


**Supplementary Fig. 2:** Global Heterogeneity of childhood UTIs Epidemiology: Number of DALYs, DALYs rates, and EAPC (1990–2021). A, Global distribution of number of DALYs, with color intensity (light to dark red-brown) indicating higher burden. B, Global distribution of DALYs rate. C, Global distribution of EAPC in DALYs rate.


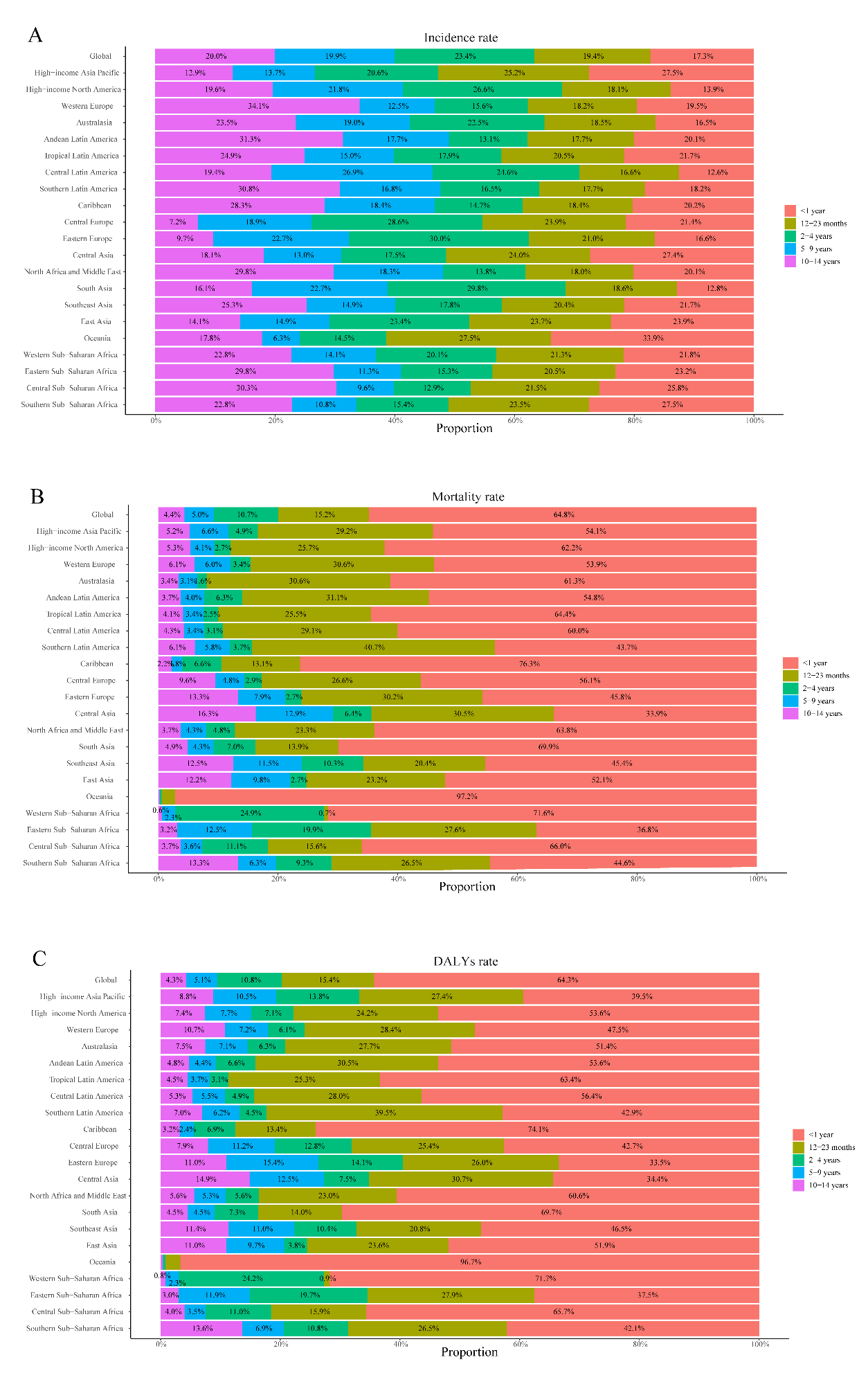


**Supplementary Fig. 3:** Age Distribution of childhood UTIs Disease Burden by Region, 2021. A, Proportional attribution of incidence rate. B, Proportional attribution of mortality rate. C, Proportional attribution of DALYs rate.


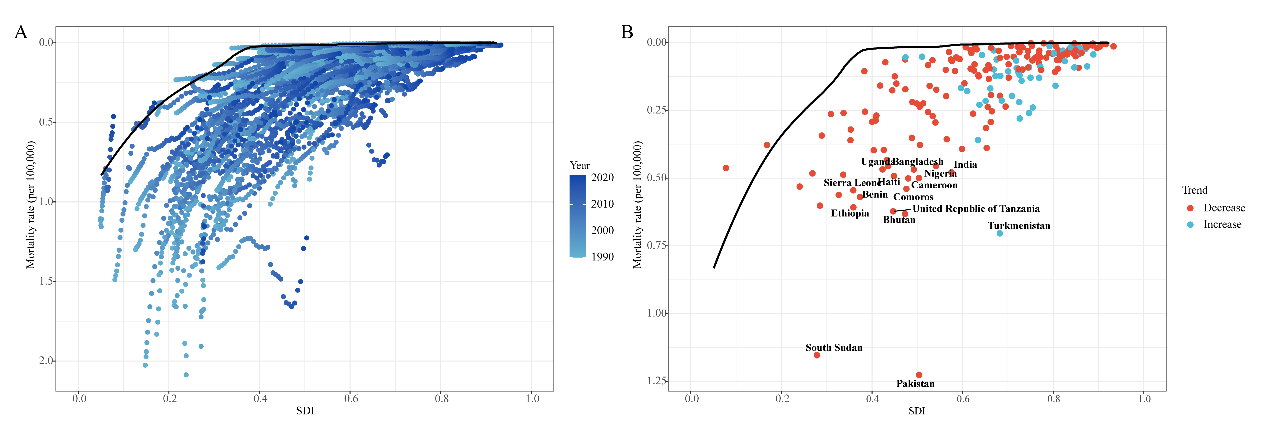


**Supplementary Fig.4:** Frontier analysis exploring the relationship between SDI and mortality rate for childhood UTIs in 204 countries and territories.


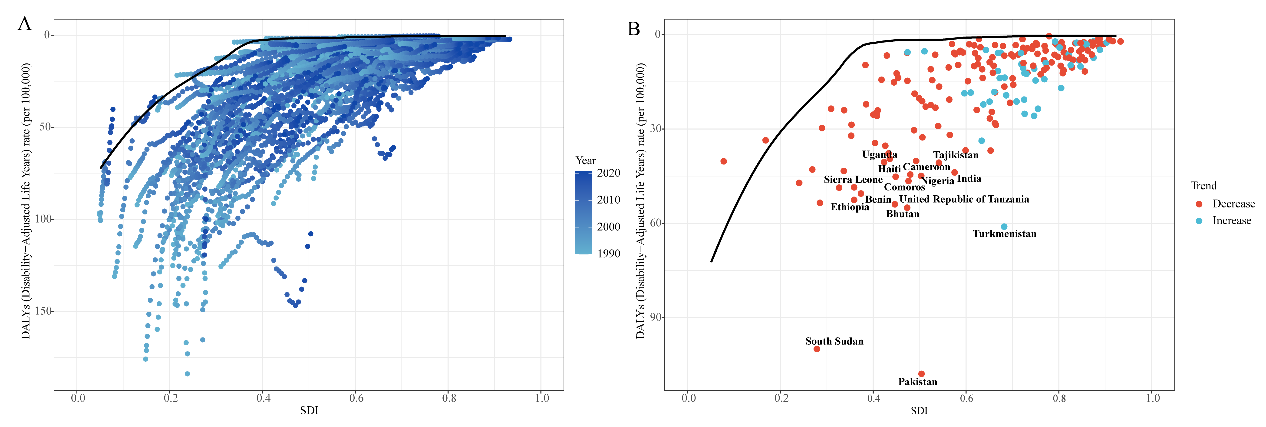


**Supplementary Fig.5:** Frontier analysis exploring the relationship between SDI and DALYs rate for childhood UTIs in 204 countries and territories.

**Supplementary Table 1:** Incidence of UTIs and in children between 1990 and 2021 at the regional level.

| Location | Rate per 100 000 (95% UI) | | | | | | |
| --- | --- | --- | --- | --- | --- | --- | --- |
|  | 1990 | | 2021 | | 1990-2021 | | |
|  | Cases | Rate | Cases | Rate | Cases change % | Rate change % | EAPC* |
| SDI |  |  |  |  |  |  |  |
| Low SDI | 4,742,658.91 (4,102,302.50, 5,653,506.69) | 2,071.81 (1,792.08, 2,469.71) | 7,750,855.48 (6,696,072.42, 8,790,013.03) | 1,684.14 (1,454.95, 1,909.94) | 63.43 (47.21, 76.20) | -18.71 (-26.78, -12.36) | -0.53(-0.90,-0.17) |
| Low-middle SDI | 14,917,132.17 (12,952,426.56, 17,549,846.17) | 3,159.66 (2,743.51, 3,717.30) | 17,309,562.33 (15,163,347.38, 19,737,405.40) | 2,985.22 (2,615.09, 3,403.93) | 16.04 (5.48, 21.53) | -5.52 (-14.12, -1.05) | -0.20(-0.40,0.01) |
| Middle SDI | 11,916,961.27 (10,426,793.00, 13,549,131.64) | 2,064.57 (1,806.40, 2,347.33) | 13,518,144.33 (11,845,739.87, 15,281,145.18) | 2,384.74 (2,089.71, 2,695.75) | 13.44 (8.47, 16.68) | 15.51 (10.45, 18.81) | 0.45(0.31,0.59) |
| High-middle SDI | 7,497,134.08 (6,648,345.90, 8,397,305.41) | 2,739.95 (2,429.75, 3,068.93) | 6,408,094.18 (5,701,694.93, 7,198,173.96) | 2,775.38 (2,469.44, 3,117.57) | -14.53 (-17.98, -11.64) | 1.29 (-2.80, 4.71) | 0.29(-0.01,0.59) |
| High SDI | 6,367,488.10 (5,633,217.53, 7,238,628.09) | 3,426.92 (3,031.74, 3,895.76) | 5,148,699.28 (4,526,943.50, 5,885,618.85) | 2,984.12 (2,623.76, 3,411.23) | -19.14 (-21.45, -17.06) | -12.92 (-15.41, -10.68) | -0.71(-0.85,-0.58) |
| Regions |  |  |  |  |  |  |  |
| Andean Latin America | 637,733.28 (538,412.09, 758,398.36) | 4,293.91 (3,625.17, 5,106.36) | 874,682.89 (731,445.36, 1,077,794.11) | 4,833.89 (4,042.30, 5,956.38) | 37.15 (22.80, 59.34) | 12.58 (0.79, 30.79) | 0.59(0.51,0.68) |
| Australasia | 174,826.10 (148,844.13, 206,182.32) | 3,812.20 (3,245.65, 4,495.95) | 224,050.13 (191,309.76, 264,668.83) | 3,909.34 (3,338.07, 4,618.08) | 28.16 (14.02, 43.42) | 2.55 (-8.76, 14.76) | 0.02(-0.01,0.05) |
| Caribbean | 496,200.38 (424,121.59, 578,152.67) | 4,347.90 (3,716.31, 5,065.99) | 466,808.11 (394,287.48, 558,120.81) | 4,057.40 (3,427.07, 4,851.07) | -5.92 (-12.23, 1.22) | -6.68 (-12.94, 0.40) | -0.23(-0.28,-0.19) |
| Central Asia | 893,714.74 (781,358.34, 1,022,909.39) | 3,576.12 (3,126.53, 4,093.08) | 1,000,661.22 (880,705.94, 1,146,626.26) | 3,615.65 (3,182.22, 4,143.07) | 11.97 (7.44, 17.16) | 1.11 (-2.98, 5.79) | 0.07(0.03,0.11) |
| Central Europe | 1,397,651.71 (1,245,921.56, 1,565,831.84) | 4,740.43 (4,225.81, 5,310.85) | 945,128.46 (833,927.10, 1,051,923.39) | 5,339.34 (4,711.13, 5,942.66) | -32.38 (-35.43, -28.93) | 12.63 (7.55, 18.37) | -0.75(-1.10,-0.40) |
| Central Latin America | 4,158,818.13 (3,577,915.75, 4,811,611.66) | 6,459.68 (5,557.39, 7,473.63) | 5,308,918.94 (4,597,359.70, 6,157,562.51) | 8,362.48 (7,241.65, 9,699.25) | 27.65 (22.65, 33.64) | 29.46 (24.38, 35.53) | 1.02(0.73,1.30) |
| Central Sub-Saharan Africa | 228,912.65 (194,682.98, 272,159.40) | 904.84 (769.54, 1,075.79) | 534,166.64 (455,986.89, 646,763.07) | 910.28 (777.05, 1,102.16) | 133.35 (118.44, 150.56) | 0.60 (-5.83, 8.02) | -0.02(-0.03,-0.00) |
| East Asia | 904,571.49 (788,071.67, 1,078,318.90) | 274.25 (238.93, 326.93) | 498,184.04 (429,582.28, 570,270.91) | 186.34 (160.68, 213.30) | -44.93 (-50.58, -41.47) | -32.05 (-39.03, -27.80) | -1.60(-1.98,-1.22) |
| Eastern Europe | 4,547,772.55 (4,029,543.01, 5,075,722.43) | 8,837.15 (7,830.14, 9,863.05) | 3,701,228.67 (3,299,980.97, 4,144,617.35) | 10,442.46 (9,310.40, 11,693.41) | -18.61 (-22.93, -14.43) | 18.17 (11.90, 24.24) | 0.72(0.29,1.15) |
| Eastern Sub-Saharan Africa | 627,562.97 (543,612.52, 730,760.57) | 692.90 (600.21, 806.84) | 1,397,306.97 (1,185,121.16, 1,652,309.64) | 783.11 (664.19, 926.02) | 122.66 (115.21, 133.05) | 13.02 (9.24, 18.29) | 0.12(0.05,0.20) |
| High-income Asia Pacific | 2,226,429.05 (1,967,097.04, 2,525,417.02) | 6,325.17 (5,588.42, 7,174.57) | 1,359,836.49 (1,202,615.39, 1,553,545.59) | 6,063.78 (5,362.70, 6,927.57) | -38.92 (-41.73, -36.39) | -4.13 (-8.54, -0.16) | 0.01(-0.08,0.11) |
| High-income North America | 1,924,581.09 (1,679,769.12, 2,225,204.39) | 3,120.39 (2,723.46, 3,607.80) | 1,845,683.68 (1,616,438.57, 2,113,576.90) | 2,812.73 (2,463.37, 3,220.99) | -4.10 (-7.33, -0.51) | -9.86 (-12.90, -6.49) | -1.02(-1.46,-0.57) |
| North Africa and Middle East | 3,775,706.39 (3,227,330.14, 4,414,644.14) | 2,687.61 (2,297.27, 3,142.42) | 4,925,490.52 (4,154,540.30, 5,856,810.74) | 2,686.80 (2,266.25, 3,194.82) | 30.45 (24.53, 36.17) | -0.03 (-4.57, 4.35) | -0.03(-0.08,0.02) |
| Oceania | 8,081.19 (6,947.83, 9,411.62) | 301.55 (259.26, 351.20) | 14,740.14 (12,209.41, 17,541.37) | 290.11 (240.30, 345.24) | 82.40 (66.65, 96.62) | -3.79 (-12.10, 3.71) | -0.09(-0.11,-0.07) |
| South Asia | 17,625,702.35 (14,974,064.45, 21,381,931.46) | 4,067.20 (3,455.33, 4,933.97) | 18,914,089.20 (16,490,026.96, 21,753,525.96) | 3,730.43 (3,252.33, 4,290.45) | 7.31 (-5.19, 13.09) | -8.28 (-18.96, -3.34) | -0.36(-0.71,-0.01) |
| Southeast Asia | 744,809.42 (635,343.24, 879,903.52) | 436.21 (372.10, 515.32) | 788,382.34 (669,954.17, 930,141.71) | 456.63 (388.03, 538.73) | 5.85 (2.19, 10.55) | 4.68 (1.06, 9.33) | -0.16(-0.31,-0.00) |
| Southern Latin America | 337,261.17 (286,235.17, 395,717.39) | 2,259.49 (1,917.64, 2,651.12) | 336,381.89 (282,128.16, 409,260.52) | 2,320.59 (1,946.31, 2,823.36) | -0.26 (-9.57, 10.47) | 2.70 (-6.88, 13.75) | -0.02(-0.17,0.12) |
| Southern Sub-Saharan Africa | 357,131.87 (309,487.33, 415,747.58) | 1,726.16 (1,495.88, 2,009.48) | 413,251.29 (355,759.11, 487,894.62) | 1,717.17 (1,478.27, 2,027.33) | 15.71 (11.25, 20.83) | -0.52 (-4.36, 3.88) | -0.04(-0.09,0.00) |
| Tropical Latin America | 2,021,894.26 (1,759,763.87, 2,346,940.70) | 3,771.21 (3,282.29, 4,377.48) | 2,537,306.73 (2,215,505.15, 2,933,404.01) | 5,055.10 (4,413.97, 5,844.24) | 25.49 (20.20, 31.37) | 34.04 (28.39, 40.33) | 1.12(1.01,1.23) |
| Western Europe | 1,341,260.33 (1,144,212.69, 1,581,272.88) | 1,888.61 (1,611.15, 2,226.57) | 1,241,827.76 (1,064,526.54, 1,453,932.86) | 1,823.04 (1,562.76, 2,134.42) | -7.41 (-11.47, -2.86) | -3.47 (-7.70, 1.28) | 0.07(-0.04,0.18) |
| Western Sub-Saharan Africa | 1,055,210.71 (912,279.56, 1,200,578.03) | 1,200.74 (1,038.10, 1,366.16) | 2,845,529.46 (2,488,332.01, 3,232,349.38) | 1,324.96 (1,158.64, 1,505.07) | 169.66 (155.63, 180.94) | 10.34 (4.60, 14.96) | -0.23(-0.47,0.00) |

SDI, Sociodemographic Index; EAPC, estimated annual percentage change; CI, confidence interval; EAPC* is expressed as 95% CIs.

**Supplementary Table 2.** Mortality of UTIs in children between 1990 and 2021 at the regional level.

| Location | Rate per 100 000 (95% UI) | | | | | | |
| --- | --- | --- | --- | --- | --- | --- | --- |
|  | 1990 | | 2021 | | 1990-2021 | | |
|  | Cases | Rate | Cases | Rate | Case change % | Rate change % | EAPC* |
| SDI |  |  |  |  |  |  |  |
| Low SDI | 2,735.37 (1,724.63, 3,618.91) | 1.19(0.75,1.58) | 2,271.83 (1,702.22, 2,834.26) | 0.49(0.37,0.62) | -16.95(-36.46,26.55) | -58.69(-68.40,-37.06) | -2.68(-2.79,-2.56) |
| Low-middle SDI | 4,403.10 (3,135.59, 5,507.50) | 0.93(0.66,1.17) | 2,819.86 (2,081.67, 3,648.18) | 0.49(0.36,0.63) | -35.96(-52.36,-1.51) | -47.86(-61.21,-19.81) | -1.68(-1.81,-1.55) |
| Middle SDI | 1,661.55 (1,391.06, 1,893.44) | 0.29(0.24,0.33) | 813.43 (690.33, 939.25) | 0.14(0.12,0.17) | -51.04(-58.97,-39.42) | -50.15(-58.22,-38.31) | -1.43(-1.68,-1.18) |
| High-middle SDI | 303.45 (255.65, 340.56) | 0.11(0.09,0.12) | 103.56 (92.35, 116.37) | 0.04(0.04,0.05) | -65.87(-70.84,-58.09) | -59.56(-65.44,-50.33) | -2.56(-2.73,-2.39) |
| High SDI | 99.80 (92.07, 108.86) | 0.05(0.05,0.06) | 54.54 (49.77, 59.82) | 0.03(0.03,0.03) | -45.35(-51.06,-38.14) | -41.15(-47.29,-33.39) | -1.23(-1.43,-1.03) |
| Regions |  |  |  |  |  |  |  |
| Andean Latin America | 102.73 (85.65, 123.47) | 0.69(0.58,0.83) | 50.17 (35.04, 66.50) | 0.28(0.19,0.37) | -51.16(-67.29,-30.64) | -59.91(-73.15,-43.07) | -2.43(-2.71,-2.14) |
| Australasia | 3.46 (3.22, 3.68) | 0.08(0.07,0.08) | 2.07 (1.78, 2.44) | 0.04(0.03,0.04) | -40.19(-49.31,-27.44) | -52.14(-59.44,-41.94) | -0.90(-1.29,-0.50) |
| Caribbean | 24.34 (13.71, 32.25) | 0.21(0.12,0.28) | 26.97 (14.72, 41.04) | 0.23(0.13,0.36) | 10.81(-30.39,65.81) | 9.92(-30.95,64.47) | 0.94(0.76,1.11) |
| Central Asia | 72.29 (64.40, 81.96) | 0.29(0.26,0.33) | 69.41 (58.90, 85.81) | 0.25(0.21,0.31) | -3.99(-20.59,18.25) | -13.30(-28.29,6.78) | 0.29(-0.20,0.78) |
| Central Europe | 27.50 (26.06, 29.09) | 0.09(0.09,0.10) | 6.46 (5.55, 7.54) | 0.04(0.03,0.04) | -76.51(-80.23,-72.19) | -60.88(-67.07,-53.68) | -2.05(-3.45,-0.63) |
| Central Latin America | 250.78 (231.20, 273.94) | 0.39(0.36,0.43) | 148.06 (115.01, 191.32) | 0.23(0.18,0.30) | -40.96(-54.49,-23.42) | -40.13(-53.85,-22.33) | -0.73(-1.18,-0.29) |
| Central Sub-Saharan Africa | 96.01 (43.74, 140.28) | 0.38(0.17,0.55) | 72.54 (49.67, 101.89) | 0.12(0.08,0.17) | -24.45(-46.28,47.80) | -67.43(-76.84,-36.28) | -3.31(-3.58,-3.05) |
| East Asia | 388.28 (239.45, 484.19) | 0.12(0.07,0.15) | 44.79 (36.30, 59.45) | 0.02(0.01,0.02) | -88.47(-91.44,-77.78) | -85.77(-89.44,-72.58) | -6.62(-6.90,-6.33) |
| Eastern Europe | 66.57 (63.73, 70.42) | 0.13(0.12,0.14) | 21.88 (20.39, 23.96) | 0.06(0.06,0.07) | -67.14(-69.29,-64.83) | -52.29(-55.41,-48.93) | -2.38(-2.95,-1.81) |
| Eastern Sub-Saharan Africa | 1,214.61 (695.37, 1,748.64) | 1.34(0.77,1.93) | 881.98 (625.21, 1,179.79) | 0.49(0.35,0.66) | -27.39(-47.45,45.29) | -63.14(-73.33,-26.25) | -3.01(-3.15,-2.87) |
| High-income Asia Pacific | 8.95 (8.03, 10.59) | 0.03(0.02,0.03) | 4.98 (4.48, 5.69) | 0.02(0.02,0.03) | -44.38(-55.37,-31.78) | -12.69(-29.94,7.08) | 0.51(0.12,0.91) |
| High-income North America | 34.11 (33.17, 35.05) | 0.06(0.05,0.06) | 23.69 (21.76, 25.83) | 0.04(0.03,0.04) | -30.54(-36.25,-24.26) | -34.71(-40.08,-28.81) | -0.91(-1.24,-0.58) |
| North Africa and Middle East | 367.93 (258.17, 457.50) | 0.26(0.18,0.33) | 157.23 (112.60, 193.45) | 0.09(0.06,0.11) | -57.27(-66.70,-41.35) | -67.25(-74.48,-55.05) | -2.93(-3.19,-2.66) |
| Oceania | 4.55 (2.46, 7.02) | 0.17(0.09,0.26) | 7.20 (4.33, 10.75) | 0.14(0.09,0.21) | 58.35(7.12,140.59) | -16.48(-43.50,26.89) | -0.42(-0.62,-0.22) |
| South Asia | 4,900.50 (3,506.94, 6,214.76) | 1.13(0.81,1.43) | 3,057.90 (2,214.02, 4,133.06) | 0.60(0.44,0.82) | -37.60(-54.47,-3.19) | -46.67(-61.09,-17.26) | -1.67(-1.80,-1.54) |
| Southeast Asia | 419.66 (268.89, 523.73) | 0.25(0.16,0.31) | 250.96 (198.01, 297.52) | 0.15(0.11,0.17) | -40.20(-52.58,-10.47) | -40.86(-53.10,-11.46) | -1.37(-1.49,-1.24) |
| Southern Latin America | 7.87 (7.36, 8.44) | 0.05(0.05,0.06) | 14.98 (12.84, 17.48) | 0.10(0.09,0.12) | 90.37(60.71,127.50) | 96.03(65.49,134.27) | 2.88(2.17,3.59) |
| Southern Sub-Saharan Africa | 9.19 (5.53, 12.59) | 0.04(0.03,0.06) | 9.07 (6.58, 11.74) | 0.04(0.03,0.05) | -1.27(-32.06,41.65) | -15.12(-41.59,21.78) | -0.21(-0.45,0.04) |
| Tropical Latin America | 305.96 (270.44, 339.17) | 0.57(0.50,0.63) | 189.75 (150.96, 228.75) | 0.38(0.30,0.46) | -37.98(-51.41,-21.55) | -33.75(-48.10,-16.21) | -0.20(-0.59,0.18) |
| Western Europe | 15.01 (14.51, 15.44) | 0.02(0.02,0.02) | 15.35 (13.88, 17.04) | 0.02(0.02,0.03) | 2.24(-8.92,14.34) | 6.60(-5.04,19.21) | 0.89(0.18,1.60) |
| Western Sub-Saharan Africa | 886.24 (465.04, 1,171.14) | 1.01(0.53,1.33) | 1,010.41 (630.48, 1,345.76) | 0.47(0.29,0.63) | 14.01(-10.71,55.05) | -53.35(-63.46,-36.55) | -2.14(-2.28,-2.01) |

SDI, Sociodemographic Index; UI, uncertainty interva; EAPC, estimated annual percentage change; CI, confidence interval; EAPC* is expressed as 95% CIs.

**Supplementary Table 3**. DALYs of UTIs in children between 1990 and 2021 at the regional level.

| Location | Rate per 100 000 (95% UI) | | | | | | |
| --- | --- | --- | --- | --- | --- | --- | --- |
|  | 1990 | | 2021 | | 1990-2021 | | |
|  | Case​ | Rate | Cases | Rate | Cases change % | Rate change % | EAPC* |
| SDI |  |  |  |  |  |  |  |
| Low SDI | 240,724.28 (153,092.49, 318,171.15) | 105.16(66.88,138.99) | 201,419.14 (151,059.72, 250,194.60) | 43.77(32.82,54.36) | -16.33(-35.71,26.79) | -58.38(-68.02,-36.94) | -2.65(-2.77,-2.54) |
| Low-middle SDI | 391,554.53 (281,528.90, 489,103.65) | 82.94(59.63,103.60) | 253,737.04 (188,554.69, 326,994.42) | 43.76(32.52,56.39) | -35.20(-51.71,-1.10) | -47.24(-60.68,-19.48) | -1.65(-1.78,-1.52) |
| Middle SDI | 150,966.62 (128,206.55, 172,191.89) | 26.15(22.21,29.83) | 77,918.57 (66,527.28, 89,765.60) | 13.75(11.74,15.84) | -48.39(-56.27,-36.77) | -47.44(-55.47,-35.62) | -1.32(-1.55,-1.10) |
| High-middle SDI | 30,848.57 (26,233.19, 35,043.39) | 11.27(9.59,12.81) | 12,896.75 (10,929.26, 15,153.60) | 5.59(4.73,6.56) | -58.19(-63.89,-50.34) | -50.46(-57.21,-41.15) | -2.01(-2.14,-1.89) |
| High SDI | 12,720.27 (11,015.33, 14,912.79) | 6.85(5.93,8.03) | 7,962.22 (6,628.05, 9,774.05) | 4.61(3.84,5.66) | -37.41(-42.61,-31.69) | -32.59(-38.20,-26.43) | -1.06(-1.16,-0.96) |
| Regions |  |  |  |  |  |  |  |
| Andean Latin America | 9,403.64 (7,951.81, 11,317.58) | 63.32(53.54,76.20) | 4,895.40 (3,605.86, 6,312.23) | 27.05(19.93,34.88) | -47.94(-64.31,-27.56) | -57.27(-70.71,-40.54) | -2.25(-2.52,-1.99) |
| Australasia | 414.50 (366.73, 482.65) | 9.04(8.00,10.52) | 322.39 (256.84, 406.30) | 5.63(4.48,7.09) | -22.22(-35.02,-7.84) | -37.76(-48.00,-26.25) | -0.58(-0.83,-0.32) |
| Caribbean | 2,456.25 (1,511.24, 3,201.88) | 21.52(13.24,28.06) | 2,658.82 (1,586.71, 3,890.27) | 23.11(13.79,33.81) | 8.25(-28.14,53.43) | 7.38(-28.72,52.19) | 0.78(0.63,0.93) |
| Central Asia | 6,710.00 (5,996.21, 7,619.93) | 26.85(23.99,30.49) | 6,447.21 (5,486.91, 7,848.50) | 23.30(19.83,28.36) | -3.92(-19.23,17.42) | -13.24(-27.07,6.03) | 0.26(-0.19,0.71) |
| Central Europe | 3,291.92 (2,913.37, 3,815.77) | 11.17(9.88,12.94) | 1,175.07 (920.99, 1,533.18) | 6.64(5.20,8.66) | -64.30(-69.22,-59.32) | -40.54(-48.72,-32.25) | -1.53(-2.20,-0.85) |
| Central Latin America | 24,564.68 (22,442.70, 27,349.14) | 38.16(34.86,42.48) | 16,211.62 (12,904.90, 20,223.48) | 25.54(20.33,31.86) | -34.00(-46.71,-18.27) | -33.07(-45.96,-17.12) | -0.49(-0.91,-0.07) |
| Central Sub-Saharan Africa | 8,573.42 (3,988.76, 12,482.51) | 33.89(15.77,49.34) | 6,628.97 (4,626.85, 9,134.22) | 11.30(7.88,15.57) | -22.68(-44.24,52.77) | -66.67(-75.96,-34.14) | -3.26(-3.51,-3.00) |
| East Asia | 33,878.19 (20,935.88, 42,107.58) | 10.27(6.35,12.77) | 4,046.30 (3,320.24, 5,284.10) | 1.51(1.24,1.98) | -88.06(-91.10,-77.41) | -85.27(-89.01,-72.12) | -6.54(-6.81,-6.28) |
| Eastern Europe | 8,593.14 (7,391.47, 10,165.46) | 16.70(14.36,19.75) | 4,273.17 (3,304.38, 5,524.90) | 12.06(9.32,15.59) | -50.27(-55.67,-44.89) | -27.80(-35.63,-19.98) | -1.07(-1.55,-0.57) |
| Eastern Sub-Saharan Africa | 105,794.14 (60,678.31, 152,527.38) | 116.81(67.00,168.41) | 76,461.71 (54,335.84, 102,250.65) | 42.85(30.45,57.31) | -27.73(-47.68,44.31) | -63.31(-73.44,-26.75) | -3.02(-3.16,-2.88) |
| High-income Asia Pacific | 2,238.62 (1,635.21, 3,000.96) | 6.36(4.65,8.53) | 1,328.48 (967.62, 1,801.20) | 5.92(4.31,8.03) | -40.66(-46.50,-35.59) | -6.85(-16.03,1.10) | 0.20(0.05,0.35) |
| High-income North America | 4,177.40 (3,704.98, 4,802.73) | 6.77(6.01,7.79) | 3,209.68 (2,720.85, 3,846.23) | 4.89(4.15,5.86) | -23.17(-28.75,-17.79) | -27.78(-33.03,-22.73) | -0.97(-1.15,-0.80) |
| North Africa and Middle East | 34,564.44 (24,603.80, 42,789.18) | 24.60(17.51,30.46) | 16,723.19 (12,747.02, 20,449.54) | 9.12(6.95,11.15) | -51.62(-61.82,-35.11) | -62.92(-70.74,-50.27) | -2.62(-2.84,-2.39) |
| Oceania | 412.70 (224.36, 635.87) | 15.40(8.37,23.73) | 654.53 (396.71, 971.97) | 12.88(7.81,19.13) | 58.60(7.76,139.68) | -16.35(-43.16,26.42) | -0.42(-0.61,-0.22) |
| South Asia | 435,394.48 (315,104.28, 551,302.23) | 100.47(72.71,127.22) | 274,673.43 (200,654.43, 370,094.69) | 54.17(39.58,72.99) | -36.91(-53.82,-3.06) | -46.08(-60.53,-17.14) | -1.64(-1.77,-1.51) |
| Southeast Asia | 35,962.00 (22,955.62, 44,743.13) | 21.06(13.44,26.20) | 21,506.30 (17,018.82, 25,496.80) | 12.46(9.86,14.77) | -40.20(-52.68,-10.11) | -40.86(-53.20,-11.11) | -1.38(-1.50,-1.25) |
| Southern Latin America | 893.70 (793.97, 1,033.32) | 5.99(5.32,6.92) | 1,481.49 (1,270.80, 1,727.18) | 10.22(8.77,11.92) | 65.77(39.25,92.98) | 70.70(43.39,98.72) | 2.33(1.74,2.93) |
| Southern Sub-Saharan Africa | 1,016.75 (677.46, 1,352.83) | 4.91(3.27,6.54) | 1,024.40 (783.81, 1,287.18) | 4.26(3.26,5.35) | 0.75(-25.66,33.09) | -13.38(-36.09,14.42) | -0.18(-0.37,-0.00) |
| Tropical Latin America | 28,281.39 (25,208.79, 31,354.90) | 52.75(47.02,58.48) | 18,041.94 (14,797.60, 21,518.10) | 35.95(29.48,42.87) | -36.21(-49.40,-20.37) | -31.86(-45.95,-14.94) | -0.18(-0.54,0.19) |
| Western Europe | 2,104.73 (1,796.28, 2,572.78) | 2.96(2.53,3.62) | 2,069.54 (1,742.13, 2,526.46) | 3.04(2.56,3.71) | -1.67(-9.71,7.84) | 2.51(-5.87,12.43) | 0.61(0.10,1.13) |
| Western Sub-Saharan Africa | 78,401.33 (41,671.62, 103,329.14) | 89.21(47.42,117.58) | 90,351.71 (57,399.13, 119,842.03) | 42.07(26.73,55.80) | 15.24(-9.27,57.08) | -52.84(-62.88,-35.72) | -2.12(-2.26,-1.99) |

SDI, Sociodemographic Index; UI, uncertainty interva; EAPC, estimated annual percentage change; CI, confidence interval; EAPC* is expressed as 95% CIs.

**Supplementary Table 4**. Incidence of UTIs in children, by countries and territories, 1990-2021

| Location | Rate per 100 000 (95% UI) | | | | |
| --- | --- | --- | --- | --- | --- |
|  | 1990 | | 2021 | | 1990-2021 |
|  | Cases | Rate | Cases | Rate | EAPC* |
| Venezuela (Bolivarian Republic of) | 468,787.74 (391,521.19, 557,604.32) | 6,608.28(5,519.09, 7,860.29) | 427,751.53(359,168.77, 519,008.18) | 6,457.67(5,422.29, 7,835.35) | -0.08(-0.10,-0.06) |
| Russian Federation | 2,658,851.28 (2,369,116.61, 2,981,510.02) | 7,662.72(6,827.71, 8,592.61) | 2,733,043.09(2,423,142.42, 3,044,455.47) | 10,480.52(9,292.13, 11,674.70) | 0.85(0.51,1.20) |
| Yemen | 156,821.51 (128,736.56, 190,725.70) | 2,210.51(1,814.63, 2,688.41) | 320,381.81(260,313.24, 392,618.23) | 2,323.54(1,887.90, 2,847.42) | 0.11(0.08,0.15) |
| Central African Republic | 10,294.68 (8,684.43, 12,154.86) | 841.98(710.28, 994.12) | 19,697.74(16,376.57, 24,435.26) | 862.49(717.07, 1,069.93) | 0.06(0.04,0.08) |
| Italy | 199,203.58 (169,531.25, 240,260.65) | 2,158.27(1,836.79, 2,603.10) | 152,343.61(133,254.03, 174,995.82) | 2,004.55(1,753.37, 2,302.61) | -0.01(-0.38,0.35) |
| Samoa | 276.36 (231.45, 325.82) | 387.83(324.81, 457.25) | 309.62(261.07, 366.95) | 387.31(326.57, 459.02) | 0.05(0.02,0.07) |
| Tajikistan | 75,804.05 (64,773.37, 88,386.21) | 3,264.54(2,789.50, 3,806.40) | 115,918.49(98,391.11, 139,930.48) | 3,234.00(2,745.01, 3,903.91) | 0.03(0.00,0.06) |
| Andorra | 180.74 (149.55, 217.76) | 1,902.36(1,574.03, 2,292.01) | 203.74(166.20, 249.48) | 2,004.15(1,634.82, 2,454.04) | 0.14(0.03,0.24) |
| Madagascar | 36,595.78 (31,436.97, 42,993.29) | 670.75(576.19, 788.01) | 81,082.43(65,914.26, 101,074.82) | 691.03(561.76, 861.42) | 0.04(0.01,0.07) |
| Poland | 571,045.03 (510,499.64, 634,124.18) | 5,962.79(5,330.58, 6,621.45) | 194,096.83(172,145.51, 217,961.07) | 3,297.76(2,924.80, 3,703.22) | -3.33(-4.14,-2.51) |
| Puerto Rico | 56,801.28 (47,465.39, 67,434.92) | 5,704.25(4,766.70, 6,772.13) | 26,561.12(21,578.28, 32,550.83) | 5,976.98(4,855.71, 7,324.83) | 0.17(0.15,0.18) |
| Gambia | 4,849.93 (4,127.70, 5,609.72) | 1,051.43(894.85, 1,216.14) | 10,629.95(8,880.32, 12,641.34) | 1,070.00(893.89, 1,272.47) | -0.00(-0.04,0.04) |
| Montenegro | 7,309.01 (6,369.36, 8,403.15) | 4,522.85(3,941.38, 5,199.90) | 5,027.67(4,408.17, 5,844.87) | 4,512.56(3,956.53, 5,246.04) | 0.01(-0.03,0.06) |
| Austria | 34,184.89 (26,453.45, 46,128.77) | 2,535.31(1,961.91, 3,421.12) | 33,209.80(25,792.88, 45,547.93) | 2,560.46(1,988.62, 3,511.73) | -0.02(-0.38,0.35) |
| Ukraine | 1,538,055.88 (1,309,168.03, 1,820,494.36) | 13,521.49(11,509.27, 16,004.49) | 773,016.56(668,487.98, 909,195.35) | 12,182.70(10,535.33, 14,328.87) | 0.63(-0.33,1.60) |
| Kuwait | 19,271.17 (15,982.39, 22,654.65) | 3,476.39(2,883.11, 4,086.74) | 29,894.33(24,769.05, 36,126.60) | 3,535.75(2,929.56, 4,272.87) | 0.09(0.05,0.13) |
| Lesotho | 10,476.97 (8,890.43, 12,167.75) | 1,534.88(1,302.45, 1,782.58) | 9,694.82(8,165.73, 11,778.36) | 1,537.67(1,295.14, 1,868.13) | -0.00(-0.02,0.01) |
| Togo | 17,196.51 (14,553.74, 20,293.44) | 975.67(825.73, 1,151.38) | 32,501.13(27,584.09, 38,614.07) | 982.19(833.60, 1,166.93) | 0.01(-0.03,0.04) |
| Philippines | 106,420.80 (91,324.25, 126,384.26) | 422.06(362.19, 501.24) | 149,020.28(127,797.99, 177,905.91) | 438.31(375.89, 523.28) | 0.07(-0.01,0.15) |
| Bermuda | 661.99 (561.99, 789.75) | 5,552.86(4,714.00, 6,624.54) | 482.61(391.38, 585.92) | 5,720.31(4,638.94, 6,944.80) | 0.08(0.06,0.11) |
| Ecuador | 161,619.16 (137,249.20, 192,163.22) | 4,181.30(3,550.81, 4,971.51) | 292,929.72(236,566.12, 356,104.25) | 5,776.70(4,665.19, 7,022.53) | 1.79(1.47,2.10) |
| Sierra Leone | 16,175.00 (13,471.98, 19,740.95) | 892.35(743.23, 1,089.08) | 32,268.27(26,881.91, 38,134.75) | 902.30(751.69, 1,066.35) | 0.11(0.07,0.14) |
| Chile | 93,643.17 (77,947.41, 110,373.16) | 2,357.63(1,962.47, 2,778.84) | 89,175.81(74,336.24, 107,876.77) | 2,441.94(2,035.58, 2,954.03) | -0.10(-0.56,0.37) |
| Lebanon | 31,992.69 (27,284.70, 38,196.48) | 3,058.93(2,608.79, 3,652.10) | 40,340.24(33,566.39, 49,991.85) | 3,156.37(2,626.36, 3,911.55) | 0.09(0.05,0.12) |
| Côte d'Ivoire | 54,707.36 (46,388.94, 64,148.67) | 959.22(813.37, 1,124.76) | 111,113.56(92,966.12, 132,570.54) | 960.21(803.39, 1,145.64) | -0.04(-0.07,-0.00) |
| Dominican Republic | 107,335.29 (89,666.50, 130,058.85) | 3,982.08(3,326.58, 4,825.11) | 117,554.06(98,072.42, 143,722.51) | 4,000.55(3,337.56, 4,891.10) | -0.00(-0.06,0.05) |
| Peru | 368,348.55 (300,917.34, 455,363.76) | 4,437.45(3,625.12, 5,485.71) | 435,358.44(358,163.61, 540,972.46) | 4,564.75(3,755.36, 5,672.12) | 0.08(0.06,0.11) |
| Brazil | 1,946,705.32 (1,694,124.96, 2,261,888.10) | 3,747.67(3,261.42, 4,354.44) | 2,446,354.39(2,135,672.62, 2,825,830.08) | 5,076.97(4,432.21, 5,864.50) | 1.16(1.04,1.28) |
| American Samoa | 78.81 (66.81, 92.14) | 414.25(351.18, 484.35) | 53.33(44.35, 63.22) | 376.03(312.71, 445.80) | -0.34(-0.36,-0.32) |
| Democratic Republic of the Congo | 160,378.52 (135,942.76, 192,291.04) | 905.90(767.87, 1,086.16) | 346,671.90(292,670.48, 417,250.57) | 912.32(770.21, 1,098.06) | -0.00(-0.02,0.01) |
| Libya | 54,645.39 (45,606.11, 66,306.28) | 3,017.52(2,518.37, 3,661.44) | 45,989.81(37,681.90, 56,837.04) | 3,083.21(2,526.24, 3,810.42) | 0.02(-0.01,0.05) |
| Dominica | 1,087.98 (918.98, 1,299.88) | 4,384.54(3,703.49, 5,238.52) | 632.56(514.46, 786.86) | 4,622.99(3,759.89, 5,750.63) | 0.11(0.07,0.15) |
| Saint Kitts and Nevis | 637.49 (538.46, 770.87) | 4,514.38(3,813.10, 5,458.87) | 453.80(373.08, 556.77) | 4,608.63(3,788.88, 5,654.39) | 0.09(0.05,0.14) |
| Malawi | 29,825.10 (25,245.09, 35,945.48) | 655.55(554.88, 790.08) | 55,405.85(45,220.35, 68,769.34) | 682.01(556.64, 846.51) | 0.13(0.09,0.17) |
| Myanmar | 47,091.56 (39,153.36, 56,586.97) | 318.70(264.97, 382.96) | 50,724.07(41,551.05, 62,160.75) | 324.85(266.10, 398.09) | -0.00(-0.03,0.03) |
| Afghanistan | 91,438.09 (76,060.63, 109,412.39) | 2,122.48(1,765.54, 2,539.71) | 294,932.63(245,565.48, 361,355.72) | 2,076.90(1,729.26, 2,544.65) | 0.17(0.10,0.24) |
| Congo | 10,366.49 (8,705.72, 12,318.10) | 984.50(826.78, 1,169.84) | 18,977.28(15,695.53, 22,396.42) | 983.65(813.55, 1,160.87) | -0.08(-0.10,-0.05) |
| Luxembourg | 1,165.82 (984.98, 1,388.89) | 1,764.47(1,490.77, 2,102.08) | 1,815.88(1,492.94, 2,204.59) | 1,793.82(1,474.81, 2,177.81) | 0.06(-0.11,0.22) |
| Mauritius | 1,662.33 (1,374.75, 2,018.04) | 503.65(416.52, 611.43) | 1,081.52(882.87, 1,312.75) | 521.46(425.67, 632.94) | 0.04(0.01,0.08) |
| Guinea | 24,494.83 (20,811.91, 28,891.20) | 890.13(756.29, 1,049.89) | 53,887.16(44,533.66, 64,681.70) | 891.32(736.61, 1,069.87) | 0.02(-0.04,0.07) |
| Paraguay | 75,188.94 (63,717.40, 88,912.42) | 4,503.46(3,816.37, 5,325.43) | 90,952.35(76,867.89, 106,122.56) | 4,530.12(3,828.61, 5,285.71) | 0.05(0.03,0.08) |
| South Africa | 242,998.21 (210,642.89, 283,269.33) | 1,784.98(1,547.31, 2,080.80) | 270,587.90(232,616.52, 318,872.46) | 1,779.58(1,529.85, 2,097.13) | -0.03(-0.07,0.01) |
| Thailand | 74,187.47 (61,510.62, 89,826.43) | 440.06(364.86, 532.82) | 44,472.82(35,934.92, 54,844.40) | 455.37(367.95, 561.57) | 0.09(0.06,0.12) |
| Uruguay | 18,513.21 (15,748.21, 22,232.22) | 2,261.63(1,923.85, 2,715.96) | 15,287.38(12,615.61, 18,672.26) | 2,317.95(1,912.85, 2,831.18) | 0.03(0.01,0.06) |
| Turkmenistan | 50,586.27 (43,815.35, 59,003.12) | 3,370.48(2,919.34, 3,931.28) | 50,449.84(42,842.29, 58,973.86) | 3,310.35(2,811.17, 3,869.67) | 0.09(0.06,0.13) |
| Belgium | 32,453.58 (26,718.65, 38,886.06) | 1,796.86(1,479.34, 2,153.01) | 34,557.25(28,500.82, 40,794.11) | 1,807.29(1,490.54, 2,133.46) | 0.26(-0.10,0.61) |
| Solomon Islands | 508.57 (432.29, 598.18) | 326.62(277.63, 384.18) | 837.75(712.27, 981.75) | 322.16(273.90, 377.53) | -0.04(-0.06,-0.01) |
| Morocco | 258,622.54 (218,390.61, 307,006.15) | 2,642.85(2,231.72, 3,137.28) | 262,406.05(218,571.20, 319,150.44) | 2,679.84(2,232.17, 3,259.34) | 0.06(0.03,0.09) |
| Namibia | 10,133.61 (8,588.71, 11,778.43) | 1,686.88(1,429.71, 1,960.68) | 13,798.55(11,371.24, 16,497.59) | 1,671.69(1,377.62, 1,998.68) | 0.02(-0.02,0.05) |
| Grenada | 1,536.94 (1,275.60, 1,864.92) | 4,600.55(3,818.29, 5,582.30) | 1,038.31(854.51, 1,300.32) | 4,756.52(3,914.54, 5,956.81) | 0.13(0.08,0.17) |
| Mozambique | 40,520.84 (34,375.66, 48,945.00) | 653.12(554.07, 788.91) | 91,957.89(76,503.02, 112,426.82) | 644.59(536.26, 788.07) | -0.00(-0.05,0.04) |
| Eswatini | 6,041.60 (5,060.34, 7,178.85) | 1,566.24(1,311.85, 1,861.06) | 6,488.32(5,387.95, 7,802.11) | 1,572.41(1,305.74, 1,890.80) | -0.07(-0.11,-0.03) |
| San Marino | 75.65 (62.89, 90.95) | 1,846.32(1,534.83, 2,219.80) | 79.62(66.65, 96.56) | 1,810.51(1,515.64, 2,195.66) | -0.01(-0.10,0.08) |
| Rwanda | 22,489.08 (18,987.36, 27,375.14) | 662.83(559.62, 806.83) | 34,405.14(28,556.19, 42,143.62) | 692.20(574.52, 847.89) | 0.11(0.06,0.15) |
| Guam | 186.16 (157.74, 217.44) | 446.37(378.23, 521.39) | 159.32(136.87, 186.65) | 435.58(374.19, 510.28) | -0.08(-0.15,-0.02) |
| Democratic People's Republic of Korea | 10,417.00 (8,669.57, 12,307.40) | 175.10(145.73, 206.87) | 7,880.99(6,526.16, 9,449.21) | 165.09(136.71, 197.94) | -0.22(-0.26,-0.18) |
| Equatorial Guinea | 1,782.50 (1,493.00, 2,167.47) | 905.21(758.19, 1,100.71) | 5,341.12(4,410.25, 6,399.12) | 913.01(753.89, 1,093.87) | 0.02(0.01,0.04) |
| Serbia | 77,444.52 (64,869.85, 90,861.68) | 3,570.79(2,991.00, 4,189.42) | 42,946.25(36,980.72, 50,425.69) | 3,234.32(2,785.05, 3,797.61) | -0.22(-0.32,-0.11) |
| Gabon | 4,300.06 (3,662.18, 5,256.76) | 1,055.22(898.69, 1,289.99) | 6,889.56(5,765.67, 8,217.44) | 1,077.94(902.10, 1,285.70) | 0.08(0.05,0.10) |
| Republic of Korea | 543,862.65 (453,746.03, 639,606.22) | 4,783.07(3,990.53, 5,625.10) | 292,474.60(245,680.50, 342,789.40) | 4,814.80(4,044.46, 5,643.09) | -0.00(-0.07,0.06) |
| Bosnia and Herzegovina | 52,078.72 (44,695.38, 60,396.85) | 4,753.47(4,079.56, 5,512.71) | 23,051.21(19,456.13, 27,066.92) | 4,700.03(3,967.01, 5,518.82) | 0.10(0.02,0.18) |
| Romania | 204,838.35 (176,969.83, 235,981.38) | 3,678.82(3,178.31, 4,238.14) | 210,590.96(183,444.49, 246,932.37) | 6,996.36(6,094.49, 8,203.71) | 0.61(0.20,1.02) |
| Liberia | 10,343.53 (8,679.86, 12,317.59) | 915.21(768.01, 1,089.88) | 20,737.19(17,080.30, 24,859.30) | 948.67(781.37, 1,137.24) | 0.03(-0.02,0.08) |
| Cook Islands | 28.41 (23.52, 33.15) | 430.84(356.64, 502.72) | 16.62(14.10, 19.73) | 438.70(372.40, 521.02) | -0.02(-0.06,0.02) |
| Malta | 1,103.85 (920.52, 1,300.54) | 1,261.24(1,051.77, 1,485.98) | 786.85(662.66, 933.93) | 1,229.18(1,035.18, 1,458.95) | -0.32(-0.54,-0.10) |
| Timor-Leste | 995.98 (841.51, 1,197.07) | 299.43(252.99, 359.88) | 1,692.40(1,408.11, 2,082.27) | 325.06(270.46, 399.94) | 0.27(0.23,0.31) |
| Tonga | 163.82 (140.16, 191.04) | 391.85(335.28, 456.96) | 154.23(130.31, 182.04) | 395.24(333.94, 466.49) | 0.02(-0.01,0.05) |
| Tuvalu | 11.42 (9.65, 13.51) | 328.46(277.66, 388.73) | 11.81(9.78, 14.14) | 316.67(262.30, 379.30) | -0.07(-0.12,-0.02) |
| Sri Lanka | 25,292.76 (21,299.59, 30,889.29) | 457.12(384.95, 558.27) | 24,572.00(20,618.84, 29,597.63) | 481.43(403.97, 579.89) | 0.08(0.04,0.11) |
| Uzbekistan | 302,570.35 (260,786.24, 352,743.65) | 3,536.50(3,048.12, 4,122.94) | 353,013.78(305,399.98, 409,412.16) | 3,498.28(3,026.44, 4,057.17) | 0.02(-0.02,0.06) |
| Cyprus | 2,399.01 (2,015.98, 2,855.10) | 1,212.03(1,018.51, 1,442.45) | 2,644.18(2,240.35, 3,218.22) | 1,209.05(1,024.40, 1,471.53) | -0.29(-0.56,-0.03) |
| Guinea-Bissau | 4,430.60 (3,693.57, 5,289.98) | 918.45(765.67, 1,096.60) | 8,340.66(7,128.28, 9,696.16) | 928.65(793.66, 1,079.57) | 0.01(-0.04,0.06) |
| Canada | 174,831.48 (149,745.00, 208,325.92) | 3,039.65(2,603.49, 3,621.99) | 190,214.88(159,785.06, 228,625.18) | 3,081.99(2,588.95, 3,704.34) | -0.04(-0.06,-0.01) |
| Ghana | 68,996.46 (59,258.60, 80,051.34) | 1,027.26(882.27, 1,191.85) | 131,073.54(111,377.78, 154,170.92) | 1,017.41(864.53, 1,196.69) | 0.01(-0.03,0.05) |
| Brunei Darussalam | 4,289.97 (3,706.56, 5,011.78) | 4,736.15(4,092.07, 5,533.03) | 4,278.36(3,627.28, 5,011.92) | 4,522.67(3,834.42, 5,298.13) | -0.17(-0.19,-0.14) |
| Netherlands | 48,629.14 (40,647.13, 58,552.78) | 1,784.38(1,491.49, 2,148.52) | 49,240.05(40,712.94, 61,388.97) | 1,835.93(1,518.00, 2,288.91) | 0.11(0.07,0.14) |
| France | 200,547.51 (167,000.22, 243,399.23) | 1,712.00(1,425.62, 2,077.81) | 209,341.17(172,648.62, 249,475.77) | 1,803.48(1,487.37, 2,149.24) | 0.12(0.09,0.16) |
| Viet Nam | 129,613.58 (110,239.35, 154,095.08) | 488.88(415.80, 581.22) | 143,065.57(113,564.38, 177,081.05) | 577.75(458.61, 715.11) | 0.60(0.51,0.69) |
| Zimbabwe | 77,496.83 (65,724.90, 92,322.38) | 1,609.05(1,364.63, 1,916.87) | 100,881.85(85,534.33, 119,942.96) | 1,602.86(1,359.01, 1,905.72) | -0.10(-0.13,-0.06) |
| United States Virgin Islands | 1,668.07 (1,354.60, 2,043.54) | 5,221.11(4,239.94, 6,396.32) | 707.78(576.09, 856.35) | 5,285.79(4,302.26, 6,395.31) | 0.05(0.01,0.08) |
| Guatemala | 216,311.21 (181,188.53, 261,918.01) | 5,326.25(4,461.42, 6,449.23) | 267,166.13(221,067.25, 328,709.71) | 5,414.77(4,480.47, 6,662.10) | 0.05(0.02,0.07) |
| Japan | 1,642,262.34 (1,454,592.44, 1,836,869.07) | 7,112.74(6,299.93, 7,955.60) | 1,016,275.64(902,408.48, 1,148,988.95) | 6,580.24(5,842.96, 7,439.54) | -0.04(-0.18,0.09) |
| Bangladesh | 672,496.86 (566,094.18, 797,417.03) | 1,374.93(1,157.39, 1,630.33) | 662,323.51(545,445.16, 801,434.24) | 1,447.20(1,191.81, 1,751.16) | 0.22(0.20,0.25) |
| Bahrain | 5,349.62 (4,421.19, 6,616.68) | 3,277.00(2,708.27, 4,053.15) | 10,054.16(8,216.24, 12,474.34) | 3,388.53(2,769.10, 4,204.19) | 0.11(0.07,0.15) |
| Colombia | 756,547.47 (642,068.55, 899,961.04) | 6,486.73(5,505.17, 7,716.37) | 689,671.38(570,984.21, 849,091.01) | 6,498.35(5,380.03, 8,000.46) | 0.02(-0.00,0.04) |
| China | 882,263.01 (769,234.25, 1,054,402.81) | 277.11(241.61, 331.18) | 484,098.32(417,347.71, 554,697.29) | 186.46(160.75, 213.65) | -1.65(-2.05,-1.26) |
| Greenland | 315.43 (262.65, 385.01) | 2,217.71(1,846.59, 2,706.86) | 264.58(215.98, 318.25) | 2,251.31(1,837.80, 2,708.05) | -0.04(-0.06,-0.01) |
| Sudan | 195,359.17 (162,444.31, 233,276.56) | 2,196.91(1,826.77, 2,623.31) | 380,307.86(309,550.27, 464,063.26) | 2,292.49(1,865.97, 2,797.37) | 0.11(0.08,0.14) |
| Saint Lucia | 2,486.59 (2,090.44, 2,957.61) | 4,824.28(4,055.70, 5,738.11) | 1,504.53(1,224.86, 1,855.04) | 5,067.30(4,125.38, 6,247.82) | 0.16(0.12,0.19) |
| Palestine | 28,169.16 (23,600.62, 33,240.92) | 2,909.17(2,437.36, 3,432.96) | 56,421.20(46,887.01, 68,155.25) | 3,021.75(2,511.13, 3,650.19) | 0.14(0.12,0.17) |
| Portugal | 34,543.15 (28,439.60, 42,091.37) | 1,632.55(1,344.09,1,989.29) | 22,648.35(19,002.02, 26,796.95) | 1,662.56(1,394.89, 1,967.10) | 0.14(0.08,0.20) |
| Spain | 145,548.86 (118,593.48, 176,686.29) | 1,857.43(1,513.44, 2,254.79) | 121,552.04(98,851.08, 146,834.90) | 1,875.58(1,525.30, 2,265.70) | -0.17(-0.27,-0.06) |
| Guyana | 11,983.58 (9,940.49, 14,271.80) | 4,076.96(3,381.88, 4,855.44) | 8,856.22(7,153.75, 10,887.68) | 4,150.30(3,352.47, 5,102.31) | 0.14(0.08,0.21) |
| Vanuatu | 229.79 (193.93, 269.97) | 337.49(284.82, 396.50) | 383.31(323.96, 460.54) | 328.90(277.97, 395.16) | -0.09(-0.11,-0.07) |
| Armenia | 41,143.08 (35,001.31, 48,205.82) | 3,943.90(3,355.17, 4,620.93) | 47,855.66(41,408.30, 54,859.60) | 8,078.73(6,990.32, 9,261.09) | 0.44(-0.04,0.93) |
| Tokelau | 2.18 (1.86, 2.54) | 363.68(309.17, 422.57) | 1.37(1.15, 1.66) | 351.90(294.36, 424.70) | -0.05(-0.15,0.04) |
| Albania | 39,704.75 (33,560.80, 45,994.02) | 3,553.78(3,003.87, 4,116.71) | 15,039.82(12,846.84, 17,698.47) | 3,389.77(2,895.50, 3,988.99) | -0.07(-0.15,0.01) |
| Saudi Arabia | 192,022.66 (157,699.24, 228,330.24) | 2,930.13(2,406.38, 3,484.16) | 228,989.54(185,401.14, 283,236.42) | 3,026.85(2,450.69, 3,743.91) | 0.08(0.03,0.13) |
| Algeria | 291,868.82 (244,033.82, 349,317.19) | 2,721.48(2,275.45, 3,257.15) | 360,816.81(296,252.68, 431,703.43) | 2,712.56(2,227.18, 3,245.48) | -0.09(-0.18,-0.01) |
| Egypt | 557,950.58 (464,314.75, 667,935.65) | 2,515.36(2,093.23, 3,011.20) | 943,264.76(763,002.48, 1,146,079.45) | 2,559.38(2,070.27, 3,109.68) | -0.02(-0.07,0.03) |
| Antigua and Barbuda | 923.96 (767.63, 1,114.29) | 5,079.34(4,219.95, 6,125.66) | 901.74(722.63, 1,116.39) | 5,335.15(4,275.46, 6,605.13) | 0.04(-0.02,0.09) |
| Mexico | 2,213,560.83 (1,897,092.45, 2,574,158.68) | 6,624.21(5,677.16, 7,703.31) | 3,363,519.99(2,909,082.56, 3,869,650.72) | 10,489.06(9,071.90, 12,067.41) | 1.89(1.35,2.42) |
| Cambodia | 14,885.23 (12,380.83, 17,706.03) | 319.36(265.63, 379.88) | 16,901.43(13,932.47, 20,959.91) | 330.33(272.30, 409.65) | 0.10(0.04,0.16) |
| United States of America | 1,749,390.04 (1,520,341.73, 2,020,713.58) | 3,128.92(2,719.25, 3,614.21) | 1,655,175.28(1,450,854.98, 1,903,964.15) | 2,784.88(2,441.11, 3,203.48) | -1.11(-1.58,-0.62) |
| India | 14,963,825.86 (12,606,317.91, 18,324,817.69) | 4,582.81(3,860.80, 5,612.14) | 14,911,592.52(13,003,210.37, 17,040,191.98) | 4,069.77(3,548.92, 4,650.72) | -0.95(-1.35,-0.55) |
| Norway | 20,350.95 (17,517.07, 24,048.51) | 2,549.13(2,194.17, 3,012.29) | 24,014.66(20,606.83, 28,678.06) | 2,599.56(2,230.67, 3,104.37) | 0.58(0.19,0.97) |
| Singapore | 36,014.10 (30,072.13, 41,232.16) | 5,545.97(4,630.94, 6,349.52) | 46,807.89(40,606.68, 54,365.20) | 5,763.86(5,000.25, 6,694.46) | 0.10(0.05,0.16) |
| Suriname | 5,379.58 (4,388.94, 6,587.66) | 4,129.66(3,369.19, 5,057.05) | 5,893.24(4,869.50, 7,204.79) | 4,113.41(3,398.85, 5,028.86) | -0.02(-0.05,0.01) |
| Iran (Islamic Republic of) | 728,237.45 (623,930.84, 851,247.67) | 2,868.82(2,457.92, 3,353.41) | 602,985.89(514,084.62, 710,773.98) | 2,988.12(2,547.57, 3,522.27) | -0.18(-0.35,-0.01) |
| Northern Mariana Islands | 60.00 (50.47, 70.44) | 493.47(415.05, 579.27) | 50.43(41.83, 59.63) | 447.71(371.36, 529.38) | -0.49(-0.64,-0.33) |
| Finland | 21,568.55 (17,813.08, 25,747.42) | 2,235.21(1,846.02, 2,668.28) | 19,833.11(16,560.61, 24,375.15) | 2,341.32(1,955.00, 2,877.51) | -0.01(-0.17,0.15) |
| Slovakia | 62,178.50 (53,373.17, 72,096.63) | 4,690.32(4,026.10, 5,438.47) | 41,349.84(35,090.46, 48,464.53) | 4,827.46(4,096.70, 5,658.08) | 0.11(-0.43,0.65) |
| Slovenia | 19,873.41 (17,166.33, 22,918.18) | 4,805.80(4,151.17, 5,542.08) | 32,753.95(28,340.53, 37,156.48) | 10,489.10(9,075.75, 11,898.96) | 2.16(1.53,2.79) |
| Eritrea | 10,662.09 (8,870.80, 12,762.09) | 669.78(557.25, 801.70) | 17,323.84(14,495.44, 21,343.47) | 686.19(574.16, 845.41) | 0.07(0.05,0.08) |
| El Salvador | 127,819.71 (105,203.74, 152,214.68) | 5,922.83(4,874.86, 7,053.22) | 108,837.86(91,346.67, 131,287.19) | 5,984.07(5,022.38, 7,218.37) | 0.06(0.03,0.09) |
| North Macedonia | 20,289.47 (17,307.42, 23,716.10) | 3,851.65(3,285.55, 4,502.14) | 12,633.38(10,874.63, 14,480.00) | 3,856.49(3,319.61, 4,420.19) | 0.09(0.05,0.13) |
| Denmark | 18,399.14 (15,271.55, 22,859.06) | 2,082.88(1,728.82, 2,587.77) | 20,141.17(16,514.44, 24,935.10) | 2,110.87(1,730.78, 2,613.30) | 0.10(-0.16,0.36) |
| Sao Tome and Principe | 609.71 (510.41, 717.21) | 1,075.97(900.73, 1,265.67) | 870.46(745.37, 1,034.39) | 1,118.38(957.67, 1,329.00) | 0.02(-0.03,0.07) |
| Republic of Moldova | 73,311.48 (61,536.64, 85,975.69) | 5,931.73(4,979.02, 6,956.41) | 30,403.68(25,541.53, 36,675.33) | 5,821.00(4,890.10, 7,021.75) | 0.09(0.02,0.16) |
| Zambia | 25,415.85 (21,482.83, 30,871.48) | 676.94(572.18, 822.24) | 56,807.72(46,426.48, 69,102.50) | 686.78(561.28, 835.42) | -0.01(-0.03,0.01) |
| Sweden | 37,434.71 (31,236.94, 44,559.41) | 2,423.83(2,022.54, 2,885.15) | 48,136.01(40,917.69, 57,449.83) | 2,643.77(2,247.32, 3,155.31) | 0.21(-0.06,0.49) |
| Qatar | 4,638.82 (3,888.69, 5,585.65) | 3,709.77(3,109.88, 4,466.97) | 18,831.60(15,945.70, 22,950.10) | 3,812.83(3,228.52, 4,646.70) | -0.40(-0.79,-0.00) |
| Israel | 26,304.51 (21,910.92, 31,389.49) | 1,715.82(1,429.23, 2,047.51) | 45,894.70(38,416.70, 55,051.85) | 1,746.55(1,461.97, 2,095.03) | -0.01(-0.04,0.02) |
| Cabo Verde | 1,888.49 (1,610.31, 2,215.26) | 1,200.37(1,023.55, 1,408.07) | 1,750.39(1,484.74, 2,087.15) | 1,222.32(1,036.82, 1,457.49) | 0.08(0.05,0.12) |
| Costa Rica | 85,659.71 (71,715.34, 101,292.23) | 7,619.67(6,379.28, 9,010.23) | 76,203.79(63,871.13, 90,400.72) | 7,490.82(6,278.53, 8,886.38) | -0.01(-0.03,0.01) |
| Cameroon | 46,450.75 (40,259.41, 54,557.51) | 951.46(824.64, 1,117.51) | 129,195.65(109,369.66, 154,658.40) | 959.36(812.14, 1,148.43) | 0.03(0.01,0.06) |
| Haiti | 84,735.86 (68,493.51, 101,541.83) | 3,123.23(2,524.56, 3,742.67) | 137,439.35(110,111.33, 167,193.15) | 3,157.51(2,529.68, 3,841.07) | 0.02(-0.02,0.06) |
| Micronesia (Federated States of) | 153.79 (128.01, 182.24) | 334.87(278.73, 396.82) | 104.45(87.43, 125.45) | 341.31(285.70, 409.95) | 0.07(0.04,0.10) |
| Burundi | 16,896.36 (14,420.19, 20,299.20) | 644.62(550.15, 774.45) | 39,420.84(31,956.99, 49,087.79) | 673.38(545.89, 838.51) | 0.04(-0.01,0.10) |
| Honduras | 126,730.56 (103,331.02, 150,743.54) | 5,735.90(4,676.82, 6,822.74) | 184,549.75(152,995.41, 218,839.14) | 5,631.38(4,668.52, 6,677.69) | 0.00(-0.02,0.03) |
| Somalia | 24,382.77 (20,507.38, 29,078.34) | 625.90(526.42, 746.43) | 63,857.30(52,100.66, 76,965.01) | 618.21(504.39, 745.10) | -0.08(-0.14,-0.02) |
| Pakistan | 1,883,945.79 (1,589,838.89, 2,313,080.69) | 3,825.89(3,228.62, 4,697.37) | 3,230,181.28(2,717,551.25, 3,834,325.16) | 3,780.55(3,180.58, 4,487.63) | 2.91(1.99,3.83) |
| Bhutan | 3,774.42 (3,231.20, 4,426.71) | 1,439.55(1,232.37, 1,688.33) | 2,828.19(2,321.80, 3,453.57) | 1,511.06(1,240.50, 1,845.19) | 0.22(0.18,0.25) |
| Jamaica | 41,272.24 (34,856.24, 49,443.16) | 4,941.56(4,173.36, 5,919.87) | 28,673.80(23,665.47, 35,076.07) | 4,910.47(4,052.78, 6,006.88) | 0.02(-0.01,0.06) |
| Oman | 24,398.15 (20,160.87, 28,920.14) | 2,903.25(2,399.04, 3,441.34) | 36,014.24(29,555.82, 43,490.78) | 2,944.72(2,416.65, 3,556.04) | -0.02(-0.09,0.06) |
| Syrian Arab Republic | 169,682.59 (141,151.06, 201,488.37) | 2,865.24(2,383.46, 3,402.31) | 113,893.65(94,010.83, 137,784.19) | 3,109.03(2,566.28, 3,761.19) | 0.34(0.27,0.41) |
| Djibouti | 1,237.05 (1,045.87, 1,459.21) | 710.48(600.68, 838.08) | 2,910.16(2,416.10, 3,527.83) | 704.35(584.77, 853.84) | -0.12(-0.15,-0.08) |
| Kyrgyzstan | 61,676.23 (51,965.28, 71,123.71) | 3,676.51(3,097.64, 4,239.67) | 83,204.03(71,490.64, 97,717.33) | 3,658.30(3,143.29, 4,296.42) | 0.09(0.03,0.15) |
| Nigeria | 595,332.11 (515,696.71, 690,647.42) | 1,521.63(1,318.09, 1,765.25) | 1,782,229.12(1,562,751.77, 2,026,204.14) | 1,754.35(1,538.31, 1,994.51) | -0.50(-0.94,-0.05) |
| Estonia | 24,009.91 (20,219.92, 28,152.77) | 6,877.57(5,791.94, 8,064.27) | 14,914.15(12,868.74, 17,393.25) | 6,900.46(5,954.09, 8,047.49) | 0.24(0.13,0.34) |
| Tunisia | 93,561.56 (77,810.57, 112,879.30) | 3,012.79(2,505.59, 3,634.84) | 83,227.77(66,473.46, 102,270.50) | 3,009.26(2,403.48, 3,697.79) | -0.02(-0.07,0.03) |
| Benin | 22,158.57 (18,250.19, 25,766.89) | 914.97(753.59, 1,063.97) | 57,912.90(49,018.45, 69,034.52) | 952.45(806.17, 1,135.36) | 0.12(0.09,0.14) |
| Mali | 36,760.35 (31,612.37, 43,624.43) | 890.12(765.47, 1,056.33) | 102,971.92(87,846.79, 122,286.52) | 889.55(758.88, 1,056.40) | 0.01(-0.01,0.04) |
| Belarus | 156,561.00 (133,535.65, 181,387.70) | 6,512.87(5,555.03, 7,545.65) | 101,897.61(84,939.30, 120,152.01) | 6,456.67(5,382.11, 7,613.34) | 0.22(0.13,0.30) |
| Lao People's Democratic Republic | 5,668.78 (4,798.79, 6,849.22) | 307.57(260.36, 371.61) | 7,286.57(6,067.12, 9,019.67) | 317.31(264.21, 392.78) | 0.12(0.08,0.17) |
| Uganda | 55,663.25 (47,317.64, 64,962.87) | 661.11(561.99, 771.57) | 133,278.34(112,605.41, 162,266.15) | 671.91(567.69, 818.05) | 0.05(0.02,0.08) |
| Bahamas | 3,918.23 (3,230.20, 4,753.72) | 4,857.72(4,004.73, 5,893.55) | 4,133.72(3,431.55, 5,166.46) | 5,091.83(4,226.92, 6,363.95) | 0.21(0.16,0.26) |
| Saint Vincent and the Grenadines | 1,837.59 (1,538.36, 2,216.11) | 4,472.46(3,744.17, 5,393.73) | 1,123.45(915.07, 1,391.75) | 4,502.71(3,667.54, 5,578.03) | 0.01(-0.02,0.03) |
| Germany | 263,141.92 (221,405.71, 320,746.00) | 2,032.64(1,710.25, 2,477.60) | 250,281.47(205,186.17, 302,925.61) | 2,091.85(1,714.94, 2,531.84) | 0.34(0.07,0.62) |
| Azerbaijan | 84,518.82 (71,987.28, 97,869.42) | 3,482.53(2,966.18, 4,032.63) | 79,349.09(67,265.19, 93,871.38) | 3,361.42(2,849.51, 3,976.62) | 0.07(0.00,0.14) |
| Chad | 26,162.59 (22,540.42, 31,154.44) | 894.03(770.25, 1,064.61) | 80,320.55(66,770.58, 95,845.25) | 890.98(740.67, 1,063.19) | -0.00(-0.02,0.02) |
| Bulgaria | 67,497.27 (58,792.99, 78,654.90) | 3,887.59(3,386.26, 4,530.23) | 73,839.46(63,588.12, 85,366.35) | 7,565.01(6,514.74, 8,745.97) | 0.59(0.16,1.03) |
| Mauritania | 9,821.12 (8,295.77, 11,552.96) | 1,062.48(897.46, 1,249.83) | 20,072.69(17,300.24, 23,758.06) | 1,083.25(933.63, 1,282.13) | 0.07(0.05,0.09) |
| Seychelles | 117.76 (99.46, 138.75) | 496.31(419.19, 584.77) | 121.52(102.08, 145.20) | 519.19(436.11, 620.32) | 0.07(0.04,0.11) |
| Marshall Islands | 72.49 (61.34, 86.80) | 330.36(279.53, 395.56) | 58.66(48.97, 70.97) | 336.01(280.50, 406.53) | 0.05(0.03,0.07) |
| Burkina Faso | 42,097.94 (35,364.32, 49,575.85) | 892.08(749.39, 1,050.55) | 93,656.95(79,671.02, 113,277.26) | 902.96(768.12, 1,092.13) | 0.05(0.03,0.08) |
| Monaco | 61.49 (51.33, 72.93) | 1,744.34(1,456.14, 2,068.97) | 90.02(75.73, 106.37) | 1,807.90(1,520.88, 2,136.19) | 0.18(0.15,0.22) |
| Fiji | 980.78 (826.37, 1,159.67) | 348.49(293.63, 412.05) | 953.14(787.10, 1,139.27) | 349.75(288.82, 418.04) | 0.11(0.08,0.15) |
| Nepal | 101,659.43 (86,271.24, 120,854.69) | 1,206.61(1,023.97, 1,434.45) | 107,163.70(92,163.62, 128,380.98) | 1,161.39(998.82, 1,391.33) | -0.44(-0.70,-0.18) |
| Indonesia | 305,302.22 (261,161.20, 362,867.62) | 450.72(385.55, 535.70) | 309,380.43(267,556.99, 364,041.35) | 459.78(397.63, 541.02) | -0.71(-1.13,-0.29) |
| South Sudan | 16,597.58 (14,085.44, 19,850.24) | 632.49(536.76, 756.44) | 27,819.08(23,007.02, 33,813.36) | 647.72(535.68, 787.28) | -0.01(-0.06,0.03) |
| Australia | 141,869.34 (119,568.99, 168,944.04) | 3,747.38(3,158.33, 4,462.54) | 182,609.37(154,942.35, 217,654.07) | 3,844.87(3,262.34, 4,582.74) | 0.05(0.02,0.08) |
| Palau | 16.72 (14.03, 19.53) | 367.06(307.95, 428.82) | 11.60(9.37, 14.15) | 355.91(287.51, 434.12) | -0.09(-0.14,-0.04) |
| Senegal | 37,463.21 (32,361.52, 44,975.63) | 1,026.03(886.31, 1,231.78) | 65,806.80(54,637.64, 78,744.10) | 1,034.61(859.01, 1,238.00) | 0.00(-0.02,0.03) |
| Greece | 36,873.98 (29,893.92, 44,959.44) | 1,822.22(1,477.28, 2,221.78) | 25,639.92(21,284.62, 31,420.17) | 1,838.31(1,526.05, 2,252.74) | -0.05(-0.10,-0.01) |
| Niger | 35,236.81 (29,555.67, 42,111.00) | 867.27(727.44, 1,036.46) | 110,157.37(94,691.47, 130,283.97) | 863.03(741.86, 1,020.71) | -0.05(-0.07,-0.02) |
| Lithuania | 58,792.94 (50,355.15, 68,237.98) | 7,077.55(6,061.80, 8,214.56) | 28,233.37(24,085.20, 32,734.00) | 6,923.52(5,906.28, 8,027.18) | -0.16(-0.56,0.24) |
| Kiribati | 85.59 (72.73, 100.27) | 289.80(246.28, 339.51) | 120.20(99.43, 146.43) | 286.09(236.65, 348.50) | -0.01(-0.03,0.02) |
| Maldives | 397.57 (334.78, 474.89) | 378.49(318.72, 452.10) | 403.15(336.28, 486.38) | 402.47(335.72, 485.56) | 0.14(0.04,0.24) |
| Comoros | 1,534.80 (1,289.33, 1,826.34) | 721.61(606.20, 858.68) | 1,752.76(1,425.31, 2,159.03) | 729.85(593.50, 899.02) | 0.09(0.06,0.11) |
| Switzerland | 18,754.47 (15,892.89, 22,474.10) | 1,622.86(1,375.24, 1,944.72) | 21,753.36(18,130.84, 26,109.22) | 1,632.53(1,360.67, 1,959.42) | 0.00(-0.12,0.12) |
| United Republic of Tanzania | 83,304.22 (70,086.90, 98,264.12) | 689.86(580.40, 813.75) | 167,315.84(137,722.74, 201,645.04) | 685.64(564.37, 826.32) | -0.01(-0.03,0.00) |
| Barbados | 3,257.22 (2,701.12, 4,020.44) | 5,223.12(4,331.39, 6,446.99) | 2,492.73(2,028.51, 3,009.08) | 5,293.54(4,307.72, 6,390.05) | 0.04(0.03,0.06) |
| Georgia | 55,924.51 (47,373.88, 65,587.84) | 4,085.93(3,461.21, 4,791.95) | 32,493.06(27,977.76, 37,398.50) | 4,415.21(3,801.66, 5,081.77) | 0.37(0.29,0.45) |
| Panama | 53,991.82 (45,456.43, 64,121.64) | 6,474.58(5,451.04, 7,689.33) | 75,010.71(61,173.59, 90,165.70) | 6,503.67(5,303.95, 7,817.66) | -0.05(-0.08,-0.02) |
| Nauru | 13.56 (11.44, 15.93) | 321.21(271.08, 377.39) | 12.84(10.76, 15.22) | 322.70(270.56, 382.59) | 0.02(-0.01,0.05) |
| Niue | 2.82 (2.35, 3.38) | 348.93(291.30, 418.16) | 1.41(1.16, 1.68) | 362.94(300.46, 432.93) | 0.13(0.06,0.20) |
| Belize | 3,492.73 (2,953.69, 4,209.02) | 4,266.70(3,608.20, 5,141.71) | 5,529.54(4,512.01, 6,831.72) | 4,491.32(3,664.84, 5,549.00) | 0.18(0.16,0.20) |
| Latvia | 38,190.06 (32,515.41, 44,093.66) | 6,712.28(5,714.90, 7,749.89) | 19,720.21(16,612.15, 22,927.56) | 6,638.52(5,592.23, 7,718.22) | 0.29(-0.04,0.61) |
| Ethiopia | 170,231.11 (147,013.74, 198,781.45) | 698.70(603.41, 815.89) | 418,413.85(356,651.99, 491,186.69) | 943.43(804.17, 1,107.51) | 0.21(0.04,0.39) |
| Angola | 41,790.40 (35,253.39, 49,539.89) | 886.37(747.72, 1,050.73) | 136,589.04(114,690.56, 164,807.77) | 895.93(752.29, 1,081.03) | -0.03(-0.06,0.00) |
| Kazakhstan | 192,856.95 (165,275.52, 222,995.37) | 3,711.48(3,180.68, 4,291.48) | 203,661.78(173,548.27, 237,461.01) | 3,753.11(3,198.17, 4,375.97) | 0.17(0.10,0.23) |
| Croatia | 49,844.91 (42,978.07, 57,854.37) | 5,050.07(4,354.35, 5,861.55) | 32,885.88(27,618.41, 37,620.68) | 5,507.21(4,625.10, 6,300.12) | 0.33(0.02,0.64) |
| Czechia | 110,709.36 (95,454.70, 127,439.47) | 5,023.82(4,331.59, 5,783.01) | 182,855.53(155,826.76, 209,426.94) | 10,653.78(9,078.99, 12,201.92) | 1.71(1.07,2.35) |
| Papua New Guinea | 4,694.39 (4,002.96, 5,537.34) | 276.13(235.46, 325.71) | 10,838.24(8,878.45, 12,997.62) | 276.69(226.66, 331.82) | 0.05(0.02,0.09) |
| Mongolia | 28,634.46 (24,380.44, 33,481.73) | 3,181.58(2,708.92, 3,720.16) | 34,715.49(29,831.49, 40,835.11) | 3,194.86(2,745.39, 3,758.05) | 0.09(0.04,0.15) |
| New Zealand | 32,956.75 (27,724.51, 38,535.85) | 4,118.93(3,465.00, 4,816.20) | 41,440.76(35,527.78, 47,564.04) | 4,221.25(3,618.94, 4,844.98) | -0.08(-0.16,-0.00) |
| Trinidad and Tobago | 18,868.87 (15,253.55, 22,779.69) | 4,643.85(3,754.08, 5,606.35) | 12,877.20(10,793.52, 15,670.30) | 4,726.96(3,962.08, 5,752.25) | -0.03(-0.06,0.01) |
| Kenya | 91,758.58 (79,724.89, 107,303.68) | 821.46(713.73, 960.63) | 204,339.46(176,593.94, 240,326.60) | 1,094.76(946.11, 1,287.56) | 0.54(0.30,0.78) |
| Nicaragua | 109,409.09 (92,672.23, 132,620.17) | 6,007.60(5,088.59, 7,282.11) | 116,207.80(95,862.21, 140,323.81) | 5,867.85(4,840.51, 7,085.58) | -0.01(-0.03,0.02) |
| Iraq | 222,630.91 (187,144.45, 266,105. | 2,702.99(2,272.15, 3,230.82) | 371,464.85(305,624.98, 457,622.00) | 2,759.38(2,270.30, 3,399.39) | 0.11(0.07,0.14) |
| Ireland | 17,890.90 (14,551.07, 21,442.07) | 1,820.94(1,481.01, 2,182.38) | 18,478.73(15,034.57, 22,208.93) | 1,853.07(1,507.68, 2,227.14) | -0.13(-0.21,-0.06) |
| Jordan | 46,943.15 (38,923.62, 55,489.51) | 2,873.97(2,383.00, 3,397.20) | 137,626.44(100,406.31, 207,572.25) | 3,788.19(2,763.70, 5,713.46) | 1.10(0.92,1.29) |
| United Kingdom | 178,144.55 (150,685.05, 211,045.65) | 1,631.29(1,379.84, 1,932.57) | 136,735.49(115,577.73, 161,997.87) | 1,160.44(980.88, 1,374.84) | 0.16(-0.65,0.97) |
| Iceland | 1,197.03 (1,008.12, 1,400.22) | 1,886.98(1,589.19, 2,207.29) | 1,313.21(1,093.78, 1,570.08) | 1,944.43(1,619.53, 2,324.77) | 0.18(0.05,0.31) |
| Cuba | 131,512.96 (109,812.75, 155,787.83) | 5,252.05(4,385.44, 6,221.48) | 94,155.05(78,206.54, 115,173.36) | 5,298.49(4,401.00, 6,481.27) | -0.02(-0.05,0.01) |
| Botswana | 9,984.65 (8,496.91, 11,701.32) | 1,691.04(1,439.07, 1,981.78) | 11,799.84(10,072.39, 13,959.08) | 1,689.85(1,442.46, 1,999.07) | 0.32(-0.14,0.79) |
| Turkey | 580,634.39 (488,260.14, 691,514.25) | 2,833.92(2,383.07, 3,375.10) | 537,386.65(442,671.92, 656,910.99) | 2,901.59(2,390.18, 3,546.95) | 0.44(0.21,0.67) |
| Hungary | 92,487.35 (80,359.34, 105,625.58) | 4,339.95(3,770.84, 4,956.45) | 64,301.00(54,757.17, 74,743.05) | 4,630.94(3,943.59, 5,382.97) | 0.18(0.15,0.20) |
| Bolivia (Plurinational State of) | 107,765.57 (89,927.08, 129,694.14) | 4,012.36(3,348.19, 4,828.81) | 146,394.73(121,185.52, 178,899.78) | 4,198.88(3,475.83, 5,131.19) | 0.17(0.12,0.22) |
| United Arab Emirates | 19,402.49 (16,103.58, 23,034.30) | 3,291.97(2,732.25, 3,908.16) | 45,666.20(38,149.81, 54,922.26) | 3,410.98(2,849.55, 4,102.35) | 0.03(0.00,0.07) |
| Argentina | 225,088.77 (189,734.27, 266,421.31) | 2,220.85(1,872.03, 2,628.66) | 231,900.13(190,673.84, 282,088.19) | 2,277.24(1,872.40, 2,770.09) | 0.04(0.02,0.06) |
| Taiwan (Province of China) | 11,891.47 (9,796.06, 14,226.94) | 215.89(177.85, 258.29) | 6,204.72(4,981.12, 7,424.43) | 210.56(169.04, 251.95) | -0.02(-0.09,0.05) |
| Malaysia | 32,095.94 (27,471.83, 37,835.20) | 488.30(417.95, 575.61) | 38,560.94(31,850.29, 47,047.87) | 506.47(418.33, 617.94) | 0.05(0.03,0.07) |

**Supplementary Table 5**. Mortality of UTIs in children, by countries and territories, 1990-2021

| Location | Rate per 100 000 (95% UI) | | | | |
| --- | --- | --- | --- | --- | --- |
|  | 1990 | | 2021 | | 1990-2021 |
|  | Cases | Rate | Cases | Rate | EAPC* |
| Venezuela (Bolivarian Republic of) | 9.80(8.67,10.96) | 0.14(0.12,0.15) | 11.10(8.08,14.64) | 0.17(0.12,0.22) | 1.56(0.96,2.17) |
| Russian Federation | 46.19(44.98,47.78) | 0.13(0.13,0.14) | 16.62(15.29,18.30) | 0.06(0.06,0.07) | -2.42(-3.00,-1.84) |
| Yemen | 16.50(8.68,25.76) | 0.23(0.12,0.36) | 17.15(9.76,25.37) | 0.12(0.07,0.18) | -1.72(-2.06,-1.39) |
| Central African Republic | 5.39(2.53,8.20) | 0.44(0.21,0.67) | 6.03(3.59,8.75) | 0.26(0.16,0.38) | -1.36(-1.50,-1.21) |
| Italy | 1.51(1.45,1.59) | 0.02(0.02,0.02) | 1.01(0.86,1.19) | 0.01(0.01,0.02) | 0.39(-0.37,1.15) |
| Samoa | 0.09(0.06,0.14) | 0.13(0.09,0.20) | 0.05(0.03,0.08) | 0.06(0.04,0.09) | -2.35(-2.44,-2.27) |
| Tajikistan | 17.39(13.15,22.39) | 0.75(0.57,0.96) | 16.33(10.91,27.64) | 0.46(0.30,0.77) | -1.96(-2.31,-1.62) |
| Andorra | 0.01(0.00,0.01) | 0.06(0.04,0.09) | 0.00(0.00,0.00) | 0.01(0.01,0.02) | -4.67(-4.97,-4.36) |
| Madagascar | 44.98(23.59,68.40) | 0.82(0.43,1.25) | 34.26(23.16,51.46) | 0.29(0.20,0.44) | -2.87(-3.04,-2.70) |
| Poland | 6.18(5.89,6.49) | 0.06(0.06,0.07) | 2.46(2.11,2.86) | 0.04(0.04,0.05) | 0.31(-2.17,2.86) |
| Puerto Rico | 0.56(0.50,0.61) | 0.06(0.05,0.06) | 0.30(0.25,0.37) | 0.07(0.06,0.08) | 1.35(0.41,2.29) |
| Gambia | 3.17(1.82,4.63) | 0.69(0.39,1.00) | 2.67(1.72,3.81) | 0.27(0.17,0.38) | -3.22(-3.55,-2.90) |
| Montenegro | 0.01(0.01,0.01) | 0.01(0.00,0.01) | 0.00(0.00,0.00) | 0.00(0.00,0.00) | -4.13(-4.59,-3.67) |
| Austria | 0.15(0.13,0.16) | 0.01(0.01,0.01) | 0.14(0.12,0.16) | 0.01(0.01,0.01) | 1.05(0.52,1.59) |
| Ukraine | 12.19(10.65,14.41) | 0.11(0.09,0.13) | 3.48(2.99,4.15) | 0.05(0.05,0.07) | -2.15(-2.57,-1.72) |
| Kuwait | 0.06(0.05,0.07) | 0.01(0.01,0.01) | 0.77(0.64,0.92) | 0.09(0.08,0.11) | 5.74(3.94,7.57) |
| Lesotho | 0.22(0.12,0.33) | 0.03(0.02,0.05) | 0.33(0.18,0.49) | 0.05(0.03,0.08) | 2.41(2.06,2.76) |
| Togo | 13.05(6.89,18.80) | 0.74(0.39,1.07) | 9.05(5.69,12.98) | 0.27(0.17,0.39) | -2.90(-3.07,-2.73) |
| Philippines | 150.57(91.63,190.03) | 0.60(0.36,0.75) | 107.14(76.10,134.66) | 0.32(0.22,0.40) | -1.37(-1.57,-1.16) |
| Bermuda | 0.00(0.00,0.00) | 0.01(0.01,0.02) | 0.00(0.00,0.00) | 0.03(0.02,0.04) | 3.24(2.51,3.97) |
| Ecuador | 4.42(4.03,4.91) | 0.11(0.10,0.13) | 8.57(6.46,11.43) | 0.17(0.13,0.23) | 1.28(0.17,2.40) |
| Sierra Leone | 29.91(12.22,45.14) | 1.65(0.67,2.49) | 19.49(10.77,28.75) | 0.55(0.30,0.80) | -3.57(-3.76,-3.38) |
| Chile | 2.58(2.39,2.80) | 0.07(0.06,0.07) | 3.24(2.76,3.98) | 0.09(0.08,0.11) | 1.76(1.24,2.28) |
| Lebanon | 2.95(2.07,3.92) | 0.28(0.20,0.37) | 1.17(0.81,1.59) | 0.09(0.06,0.12) | -3.57(-3.91,-3.23) |
| Côte d'Ivoire | 46.91(28.25,65.26) | 0.82(0.50,1.14) | 45.83(29.92,63.83) | 0.40(0.26,0.55) | -1.99(-2.26,-1.72) |
| Dominican Republic | 2.64(1.88,3.74) | 0.10(0.07,0.14) | 1.13(0.66,2.03) | 0.04(0.02,0.07) | -2.49(-2.80,-2.17) |
| Peru | 71.08(57.71,88.37) | 0.86(0.70,1.06) | 27.92(17.87,38.78) | 0.29(0.19,0.41) | -2.78(-3.09,-2.46) |
| Brazil | 303.78(268.06,337.15) | 0.58(0.52,0.65) | 187.25(148.97,225.75) | 0.39(0.31,0.47) | -0.18(-0.57,0.21) |
| American Samoa | 0.04(0.03,0.06) | 0.23(0.17,0.30) | 0.01(0.01,0.02) | 0.10(0.07,0.14) | -2.77(-2.98,-2.55) |
| Democratic Republic of the Congo | 64.72(29.99,97.13) | 0.37(0.17,0.55) | 39.92(26.42,57.90) | 0.11(0.07,0.15) | -3.57(-3.85,-3.28) |
| Libya | 2.01(1.39,2.79) | 0.11(0.08,0.15) | 1.79(1.00,2.82) | 0.12(0.07,0.19) | 1.10(0.84,1.36) |
| Dominica | 0.01(0.01,0.01) | 0.03(0.02,0.04) | 0.01(0.01,0.02) | 0.09(0.05,0.13) | 4.40(4.02,4.78) |
| Saint Kitts and Nevis | 0.02(0.01,0.02) | 0.11(0.10,0.13) | 0.02(0.02,0.03) | 0.24(0.19,0.30) | 3.32(2.58,4.06) |
| Malawi | 39.62(19.28,58.65) | 0.87(0.42,1.29) | 20.69(14.48,30.56) | 0.25(0.18,0.38) | -3.76(-3.98,-3.53) |
| Myanmar | 83.52(39.83,118.10) | 0.57(0.27,0.80) | 42.35(30.35,56.01) | 0.27(0.19,0.36) | -2.54(-2.81,-2.26) |
| Afghanistan | 19.35(12.00,27.54) | 0.45(0.28,0.64) | 36.86(21.89,53.97) | 0.26(0.15,0.38) | -1.43(-1.66,-1.21) |
| Congo | 3.05(1.63,4.58) | 0.29(0.15,0.44) | 2.02(1.33,2.88) | 0.10(0.07,0.15) | -3.43(-3.91,-2.96) |
| Luxembourg | 0.00(0.00,0.01) | 0.01(0.01,0.01) | 0.01(0.00,0.01) | 0.01(0.00,0.01) | -0.58(-0.97,-0.20) |
| Mauritius | 0.06(0.06,0.07) | 0.02(0.02,0.02) | 0.14(0.11,0.17) | 0.07(0.05,0.08) | 4.54(3.89,5.20) |
| Guinea | 40.14(17.70,58.69) | 1.46(0.64,2.13) | 29.47(17.39,41.83) | 0.49(0.29,0.69) | -2.89(-3.08,-2.71) |
| Paraguay | 2.18(1.34,3.14) | 0.13(0.08,0.19) | 2.50(1.55,3.82) | 0.12(0.08,0.19) | -0.22(-0.50,0.06) |
| South Africa | 6.86(4.25,9.67) | 0.05(0.03,0.07) | 4.57(3.45,6.62) | 0.03(0.02,0.04) | -1.57(-1.86,-1.27) |
| Thailand | 35.07(20.98,59.71) | 0.21(0.12,0.35) | 19.13(15.47,23.54) | 0.20(0.16,0.24) | 0.10(-0.10,0.31) |
| Uruguay | 0.38(0.35,0.42) | 0.05(0.04,0.05) | 0.62(0.50,0.74) | 0.09(0.08,0.11) | 2.16(1.27,3.06) |
| Turkmenistan | 5.63(4.91,6.53) | 0.38(0.33,0.43) | 10.73(8.81,13.02) | 0.70(0.58,0.85) | 2.25(1.81,2.69) |
| Belgium | 0.32(0.28,0.35) | 0.02(0.02,0.02) | 0.39(0.33,0.46) | 0.02(0.02,0.02) | 0.84(0.38,1.31) |
| Solomon Islands | 0.21(0.14,0.33) | 0.14(0.09,0.21) | 0.19(0.12,0.32) | 0.07(0.05,0.12) | -2.17(-2.44,-1.91) |
| Morocco | 26.41(15.65,37.29) | 0.27(0.16,0.38) | 9.83(6.36,13.45) | 0.10(0.06,0.14) | -2.62(-3.11,-2.13) |
| Namibia | 0.18(0.09,0.30) | 0.03(0.02,0.05) | 0.23(0.10,0.36) | 0.03(0.01,0.04) | 0.30(-0.03,0.62) |
| Grenada | 0.02(0.02,0.03) | 0.06(0.05,0.08) | 0.02(0.02,0.03) | 0.10(0.08,0.13) | 2.25(1.73,2.78) |
| Mozambique | 113.62(57.72,189.24) | 1.83(0.93,3.05) | 80.19(51.00,135.41) | 0.56(0.36,0.95) | -3.31(-3.54,-3.09) |
| Eswatini | 0.23(0.13,0.34) | 0.06(0.03,0.09) | 0.23(0.12,0.34) | 0.05(0.03,0.08) | 0.03(-0.11,0.17) |
| San Marino | 0.00(0.00,0.00) | 0.01(0.01,0.01) | 0.00(0.00,0.00) | 0.00(0.00,0.01) | -2.38(-2.60,-2.16) |
| Rwanda | 50.60(28.36,84.86) | 1.49(0.84,2.50) | 22.66(14.73,33.28) | 0.46(0.30,0.67) | -4.39(-4.77,-4.01) |
| Guam | 0.05(0.04,0.07) | 0.12(0.09,0.16) | 0.04(0.03,0.05) | 0.11(0.08,0.14) | 0.28(-0.16,0.73) |
| Democratic People's Republic of Korea | 5.86(3.70,8.76) | 0.10(0.06,0.15) | 1.65(0.91,3.24) | 0.03(0.02,0.07) | -3.15(-3.39,-2.91) |
| Equatorial Guinea | 0.66(0.32,1.02) | 0.34(0.16,0.52) | 0.78(0.45,1.26) | 0.13(0.08,0.22) | -3.23(-3.44,-3.02) |
| Serbia | 0.57(0.35,0.92) | 0.03(0.02,0.04) | 0.08(0.05,0.14) | 0.01(0.00,0.01) | -4.55(-5.22,-3.87) |
| Gabon | 0.85(0.48,1.28) | 0.21(0.12,0.31) | 0.77(0.48,1.17) | 0.12(0.07,0.18) | -1.18(-1.60,-0.75) |
| Republic of Korea | 3.22(2.34,4.83) | 0.03(0.02,0.04) | 0.57(0.34,0.85) | 0.01(0.01,0.01) | -2.92(-3.28,-2.55) |
| Bosnia and Herzegovina | 0.29(0.19,0.41) | 0.03(0.02,0.04) | 0.06(0.04,0.10) | 0.01(0.01,0.02) | -3.05(-3.82,-2.28) |
| Romania | 8.65(7.61,9.64) | 0.16(0.14,0.17) | 1.22(1.03,1.48) | 0.04(0.03,0.05) | -3.58(-4.64,-2.50) |
| Liberia | 21.35(10.17,30.77) | 1.89(0.90,2.72) | 7.87(4.61,11.41) | 0.36(0.21,0.52) | -4.71(-5.45,-3.97) |
| Cook Islands | 0.00(0.00,0.00) | 0.01(0.00,0.01) | 0.00(0.00,0.00) | 0.00(0.00,0.01) | -6.85(-7.91,-5.77) |
| Malta | 0.02(0.02,0.03) | 0.03(0.02,0.03) | 0.03(0.02,0.03) | 0.04(0.03,0.05) | 2.04(1.60,2.47) |
| Timor-Leste | 1.59(0.72,2.29) | 0.48(0.22,0.69) | 0.92(0.67,1.20) | 0.18(0.13,0.23) | -3.54(-3.86,-3.22) |
| Tonga | 0.05(0.03,0.07) | 0.12(0.08,0.18) | 0.03(0.02,0.05) | 0.08(0.05,0.14) | -1.29(-1.61,-0.96) |
| Tuvalu | 0.01(0.01,0.02) | 0.29(0.15,0.45) | 0.00(0.00,0.00) | 0.07(0.04,0.10) | -4.50(-4.77,-4.22) |
| Sri Lanka | 8.10(5.77,10.61) | 0.15(0.10,0.19) | 4.06(2.58,5.68) | 0.08(0.05,0.11) | -1.45(-2.04,-0.86) |
| Uzbekistan | 19.76(16.05,24.73) | 0.23(0.19,0.29) | 19.52(15.98,24.08) | 0.19(0.16,0.24) | 1.47(0.32,2.63) |
| Cyprus | 0.19(0.15,0.25) | 0.10(0.07,0.13) | 0.05(0.04,0.07) | 0.02(0.02,0.03) | -3.92(-4.37,-3.48) |
| Guinea-Bissau | 6.22(3.15,9.38) | 1.29(0.65,1.94) | 2.88(1.88,4.11) | 0.32(0.21,0.46) | -4.05(-4.42,-3.67) |
| Canada | 1.83(1.67,1.97) | 0.03(0.03,0.03) | 1.06(0.91,1.22) | 0.02(0.01,0.02) | -1.19(-1.54,-0.83) |
| Ghana | 42.39(21.99,63.45) | 0.63(0.33,0.94) | 45.92(27.38,84.47) | 0.36(0.21,0.66) | -1.10(-1.35,-0.84) |
| Brunei Darussalam | 0.09(0.06,0.14) | 0.10(0.07,0.16) | 0.09(0.06,0.13) | 0.10(0.07,0.14) | 1.05(0.60,1.50) |
| Netherlands | 1.08(0.98,1.19) | 0.04(0.04,0.04) | 1.16(1.00,1.31) | 0.04(0.04,0.05) | 0.65(0.21,1.09) |
| France | 1.79(1.63,1.96) | 0.02(0.01,0.02) | 2.01(1.73,2.28) | 0.02(0.01,0.02) | 0.90(0.48,1.31) |
| Viet Nam | 5.14(3.53,8.32) | 0.02(0.01,0.03) | 3.23(2.27,4.56) | 0.01(0.01,0.02) | -1.00(-1.12,-0.88) |
| Zimbabwe | 1.49(0.71,2.26) | 0.03(0.01,0.05) | 3.41(1.64,5.19) | 0.05(0.03,0.08) | 3.06(2.49,3.63) |
| United States Virgin Islands | 0.03(0.02,0.05) | 0.10(0.07,0.16) | 0.01(0.00,0.01) | 0.05(0.03,0.08) | -1.86(-2.35,-1.36) |
| Guatemala | 14.83(13.04,16.99) | 0.37(0.32,0.42) | 14.53(11.31,18.65) | 0.29(0.23,0.38) | 0.48(-0.11,1.08) |
| Japan | 4.71(4.54,4.87) | 0.02(0.02,0.02) | 3.56(3.23,3.94) | 0.02(0.02,0.03) | 1.52(1.10,1.95) |
| Bangladesh | 763.14(419.31,1018.21) | 1.56(0.86,2.08) | 214.33(163.20,279.45) | 0.47(0.36,0.61) | -3.54(-3.71,-3.38) |
| Bahrain | 0.03(0.01,0.05) | 0.02(0.01,0.03) | 0.03(0.02,0.06) | 0.01(0.01,0.02) | -2.31(-3.35,-1.26) |
| Colombia | 38.23(32.85,44.57) | 0.33(0.28,0.38) | 25.23(18.80,33.51) | 0.24(0.18,0.32) | 0.13(-0.25,0.52) |
| China | 377.82(228.54,473.53) | 0.12(0.07,0.15) | 40.55(32.74,53.83) | 0.02(0.01,0.02) | -6.95(-7.26,-6.63) |
| Greenland | 0.02(0.01,0.02) | 0.11(0.07,0.17) | 0.00(0.00,0.01) | 0.04(0.03,0.07) | -3.34(-3.57,-3.10) |
| Sudan | 33.32(16.51,51.30) | 0.37(0.19,0.58) | 28.81(16.37,42.17) | 0.17(0.10,0.25) | -2.03(-2.38,-1.69) |
| Saint Lucia | 0.03(0.03,0.04) | 0.06(0.05,0.08) | 0.04(0.03,0.05) | 0.12(0.09,0.16) | 2.96(2.25,3.67) |
| Palestine | 0.58(0.31,0.97) | 0.06(0.03,0.10) | 0.46(0.31,0.69) | 0.02(0.02,0.04) | -2.15(-2.44,-1.85) |
| Portugal | 0.99(0.90,1.08) | 0.05(0.04,0.05) | 0.41(0.36,0.48) | 0.03(0.03,0.04) | -1.53(-1.90,-1.15) |
| Spain | 2.33(2.14,2.53) | 0.03(0.03,0.03) | 1.91(1.67,2.23) | 0.03(0.03,0.03) | 0.27(-0.27,0.82) |
| Guyana | 0.37(0.30,0.45) | 0.13(0.10,0.15) | 0.46(0.34,0.61) | 0.22(0.16,0.29) | 3.04(2.26,3.83) |
| Vanuatu | 0.07(0.04,0.11) | 0.10(0.07,0.15) | 0.07(0.05,0.11) | 0.06(0.04,0.09) | -1.62(-2.11,-1.13) |
| Armenia | 2.55(2.05,3.21) | 0.24(0.20,0.31) | 0.77(0.61,0.97) | 0.13(0.10,0.16) | 0.52(-0.59,1.65) |
| Tokelau | 0.00(0.00,0.00) | 0.07(0.04,0.11) | 0.00(0.00,0.00) | 0.21(0.14,0.40) | -0.85(-2.68,1.01) |
| Albania | 0.72(0.30,1.23) | 0.06(0.03,0.11) | 0.06(0.03,0.08) | 0.01(0.01,0.02) | -5.03(-5.54,-4.51) |
| Saudi Arabia | 23.98(16.11,32.93) | 0.37(0.25,0.50) | 4.70(3.23,7.25) | 0.06(0.04,0.10) | -5.94(-6.21,-5.67) |
| Algeria | 22.46(15.33,30.35) | 0.21(0.14,0.28) | 12.58(8.85,17.08) | 0.09(0.07,0.13) | -1.54(-1.90,-1.18) |
| Egypt | 5.20(3.11,8.68) | 0.02(0.01,0.04) | 6.22(4.09,8.88) | 0.02(0.01,0.02) | 0.93(0.20,1.68) |
| Antigua and Barbuda | 0.01(0.01,0.01) | 0.04(0.04,0.05) | 0.02(0.02,0.03) | 0.13(0.11,0.15) | 3.96(3.02,4.90) |
| Mexico | 147.34(133.69,163.29) | 0.44(0.40,0.49) | 81.27(62.56,104.91) | 0.25(0.20,0.33) | -0.83(-1.34,-0.32) |
| Cambodia | 20.42(11.97,27.55) | 0.44(0.26,0.59) | 8.78(6.62,11.28) | 0.17(0.13,0.22) | -3.30(-3.46,-3.14) |
| United States of America | 32.26(31.39,33.22) | 0.06(0.06,0.06) | 22.63(20.77,24.66) | 0.04(0.03,0.04) | -0.90(-1.22,-0.57) |
| India | 3,337.17(2,250.00, 4,295.58) | 1.02(0.69,1.32) | 1,754.60(1,039.00, 2,709.96) | 0.48(0.28,0.74) | -2.25(-2.39,-2.10) |
| Norway | 0.12(0.11,0.13) | 0.02(0.01,0.02) | 0.05(0.05,0.06) | 0.01(0.00,0.01) | -2.16(-2.66,-1.67) |
| Singapore | 0.93(0.85,1.01) | 0.14(0.13,0.16) | 0.75(0.64,0.90) | 0.09(0.08,0.11) | -0.32(-0.91,0.26) |
| Suriname | 0.40(0.31,0.56) | 0.31(0.24,0.43) | 0.52(0.32,0.72) | 0.36(0.22,0.50) | 0.57(0.24,0.89) |
| Iran (Islamic Republic of) | 105.24(57.80,135.68) | 0.41(0.23,0.53) | 10.55(8.59,13.09) | 0.05(0.04,0.06) | -4.29(-5.25,-3.31) |
| Northern Mariana Islands | 0.02(0.01,0.02) | 0.13(0.09,0.19) | 0.01(0.00,0.01) | 0.05(0.04,0.07) | -2.94(-3.49,-2.39) |
| Finland | 0.17(0.15,0.19) | 0.02(0.02,0.02) | 0.16(0.14,0.20) | 0.02(0.02,0.02) | 0.59(-0.04,1.23) |
| Slovakia | 0.89(0.71,1.12) | 0.07(0.05,0.08) | 0.24(0.18,0.31) | 0.03(0.02,0.04) | -2.33(-2.66,-2.00) |
| Slovenia | 0.12(0.10,0.14) | 0.03(0.02,0.03) | 0.05(0.03,0.06) | 0.01(0.01,0.02) | -1.49(-3.29,0.35) |
| Eritrea | 12.07(6.61,18.87) | 0.76(0.42,1.19) | 10.02(5.95,16.79) | 0.40(0.24,0.67) | -1.99(-2.18,-1.80) |
| El Salvador | 13.99(10.84,17.56) | 0.65(0.50,0.81) | 3.59(2.54,4.97) | 0.20(0.14,0.27) | -3.32(-3.77,-2.86) |
| North Macedonia | 0.09(0.05,0.13) | 0.02(0.01,0.02) | 0.01(0.00,0.01) | 0.00(0.00,0.00) | -5.48(-6.36,-4.60) |
| Denmark | 0.24(0.21,0.27) | 0.03(0.02,0.03) | 0.19(0.16,0.22) | 0.02(0.02,0.02) | -0.43(-0.76,-0.11) |
| Sao Tome and Principe | 0.66(0.45,0.91) | 1.17(0.79,1.60) | 0.18(0.10,0.47) | 0.24(0.13,0.60) | -4.93(-5.44,-4.41) |
| Republic of Moldova | 2.72(2.38,3.15) | 0.22(0.19,0.25) | 0.52(0.41,0.67) | 0.10(0.08,0.13) | -2.37(-3.56,-1.16) |
| Zambia | 42.04(21.28,63.60) | 1.12(0.57,1.69) | 31.20(20.18,50.59) | 0.38(0.24,0.61) | -3.23(-3.40,-3.06) |
| Sweden | 0.58(0.53,0.63) | 0.04(0.03,0.04) | 0.18(0.16,0.21) | 0.01(0.01,0.01) | -3.31(-3.78,-2.83) |
| Qatar | 0.02(0.00,0.03) | 0.01(0.00,0.02) | 0.01(0.01,0.02) | 0.00(0.00,0.00) | -6.42(-7.26,-5.58) |
| Israel | 0.84(0.77,0.92) | 0.05(0.05,0.06) | 0.88(0.75,1.03) | 0.03(0.03,0.04) | -0.41(-0.92,0.11) |
| Cabo Verde | 0.36(0.17,0.53) | 0.23(0.11,0.33) | 0.09(0.06,0.15) | 0.06(0.04,0.10) | -4.30(-4.56,-4.04) |
| Costa Rica | 1.17(1.05,1.32) | 0.10(0.09,0.12) | 1.08(0.90,1.28) | 0.11(0.09,0.13) | 1.04(0.52,1.56) |
| Cameroon | 56.42(32.91,78.40) | 1.16(0.67,1.61) | 67.49(42.15,96.46) | 0.50(0.31,0.72) | -2.20(-2.42,-1.98) |
| Haiti | 17.60(7.47,24.85) | 0.65(0.28,0.92) | 21.43(9.61,34.63) | 0.49(0.22,0.80) | -0.29(-0.52,-0.06) |
| Micronesia (Federated States of) | 0.06(0.04,0.09) | 0.14(0.10,0.19) | 0.02(0.01,0.03) | 0.06(0.04,0.09) | -3.11(-3.30,-2.92) |
| Burundi | 31.23(15.14,51.28) | 1.19(0.58,1.96) | 20.08(11.43,34.67) | 0.34(0.20,0.59) | -3.22(-3.62,-2.82) |
| Honduras | 19.04(15.01,24.00) | 0.86(0.68,1.09) | 7.34(3.93,14.57) | 0.22(0.12,0.44) | -4.18(-4.24,-4.12) |
| Somalia | 43.07(22.05,72.44) | 1.11(0.57,1.86) | 47.79(25.47,76.04) | 0.46(0.25,0.74) | -2.20(-2.62,-1.77) |
| Pakistan | 695.44(507.02,931.59) | 1.41(1.03,1.89) | 1,047.78(781.05, 1,423.87) | 1.23(0.91,1.67) | 0.50(0.16,0.84) |
| Bhutan | 3.18(1.59,4.93) | 1.21(0.61,1.88) | 1.18(0.76,1.79) | 0.63(0.40,0.96) | -2.38(-2.71,-2.05) |
| Jamaica | 0.82(0.70,1.00) | 0.10(0.08,0.12) | 0.72(0.52,0.95) | 0.12(0.09,0.16) | 1.45(0.87,2.03) |
| Oman | 1.96(1.29,2.64) | 0.23(0.15,0.31) | 1.23(0.93,1.56) | 0.10(0.08,0.13) | -1.14(-1.87,-0.40) |
| Syrian Arab Republic | 48.85(32.53,64.80) | 0.82(0.55,1.09) | 9.60(7.03,13.77) | 0.26(0.19,0.38) | -3.32(-4.03,-2.59) |
| Djibouti | 1.13(0.63,1.76) | 0.65(0.36,1.01) | 1.45(0.89,2.24) | 0.35(0.21,0.54) | -1.90(-2.39,-1.40) |
| Kyrgyzstan | 4.98(4.34,5.65) | 0.30(0.26,0.34) | 3.41(3.00,3.82) | 0.15(0.13,0.17) | -0.56(-1.53,0.43) |
| Nigeria | 362.78(177.98,486.46) | 0.93(0.45,1.24) | 508.00(281.99,727.69) | 0.50(0.28,0.72) | -1.70(-1.84,-1.56) |
| Estonia | 0.61(0.56,0.68) | 0.18(0.16,0.19) | 0.09(0.08,0.10) | 0.04(0.04,0.05) | -4.07(-5.10,-3.03) |
| Tunisia | 5.71(3.76,7.62) | 0.18(0.12,0.25) | 1.54(1.11,2.12) | 0.06(0.04,0.08) | -3.19(-3.42,-2.96) |
| Benin | 30.04(16.06,43.14) | 1.24(0.66,1.78) | 34.63(21.51,50.26) | 0.57(0.35,0.83) | -2.16(-2.34,-1.97) |
| Mali | 53.72(20.57,81.11) | 1.30(0.50,1.96) | 55.86(27.56,81.70) | 0.48(0.24,0.71) | -2.97(-3.10,-2.84) |
| Belarus | 2.93(2.47,3.40) | 0.12(0.10,0.14) | 0.87(0.69,1.05) | 0.06(0.04,0.07) | -2.31(-3.08,-1.53) |
| Lao People's Democratic Republic | 10.26(5.49,14.38) | 0.56(0.30,0.78) | 5.03(3.54,6.99) | 0.22(0.15,0.30) | -3.16(-3.31,-3.02) |
| Uganda | 71.61(38.22,114.32) | 0.85(0.45,1.36) | 92.80(57.34,139.01) | 0.47(0.29,0.70) | -1.55(-1.78,-1.31) |
| Bahamas | 0.07(0.06,0.09) | 0.09(0.07,0.11) | 0.13(0.10,0.17) | 0.16(0.12,0.21) | 2.15(1.34,2.97) |
| Saint Vincent and the Grenadines | 0.05(0.04,0.06) | 0.12(0.10,0.15) | 0.06(0.05,0.07) | 0.23(0.18,0.29) | 1.93(1.43,2.43) |
| Germany | 1.92(1.72,2.13) | 0.01(0.01,0.02) | 1.75(1.56,1.97) | 0.01(0.01,0.02) | 0.95(0.51,1.39) |
| Azerbaijan | 7.94(6.25,9.96) | 0.33(0.26,0.41) | 5.57(4.14,7.10) | 0.24(0.18,0.30) | -1.30(-1.74,-0.85) |
| Chad | 25.59(10.68,37.80) | 0.87(0.36,1.29) | 47.90(20.11,74.78) | 0.53(0.22,0.83) | -1.54(-1.61,-1.47) |
| Bulgaria | 3.44(2.98,3.90) | 0.20(0.17,0.22) | 0.72(0.59,0.90) | 0.07(0.06,0.09) | -2.33(-3.37,-1.27) |
| Mauritania | 5.77(3.68,8.09) | 0.62(0.40,0.87) | 4.16(2.70,5.85) | 0.22(0.15,0.32) | -3.25(-3.61,-2.89) |
| Seychelles | 0.02(0.01,0.04) | 0.09(0.06,0.16) | 0.03(0.02,0.04) | 0.14(0.08,0.19) | 2.88(2.19,3.58) |
| Marshall Islands | 0.02(0.01,0.03) | 0.08(0.05,0.12) | 0.01(0.01,0.02) | 0.06(0.04,0.10) | -0.92(-1.55,-0.29) |
| Burkina Faso | 49.17(22.47,73.70) | 1.04(0.48,1.56) | 62.40(32.68,90.35) | 0.60(0.32,0.87) | -1.45(-1.63,-1.27) |
| Monaco | 0.00(0.00,0.00) | 0.01(0.00,0.01) | 0.00(0.00,0.00) | 0.00(0.00,0.01) | -1.88(-2.47,-1.28) |
| Fiji | 0.11(0.08,0.16) | 0.04(0.03,0.06) | 0.17(0.11,0.27) | 0.06(0.04,0.10) | 2.39(1.71,3.08) |
| Nepal | 101.57(61.77,137.94) | 1.21(0.73,1.64) | 40.01(27.86,57.55) | 0.43(0.30,0.62) | -2.90(-3.10,-2.70) |
| Indonesia | 98.28(62.71,128.19) | 0.15(0.09,0.19) | 55.83(44.34,68.88) | 0.08(0.07,0.10) | -1.66(-1.78,-1.54) |
| South Sudan | 37.51(14.26,63.53) | 1.43(0.54,2.42) | 49.56(30.45,77.44) | 1.15(0.71,1.80) | -0.43(-1.16,0.31) |
| Australia | 2.37(2.16,2.57) | 0.06(0.06,0.07) | 1.58(1.32,1.91) | 0.03(0.03,0.04) | -0.72(-1.17,-0.27) |
| Palau | 0.01(0.00,0.01) | 0.13(0.08,0.21) | 0.00(0.00,0.00) | 0.07(0.05,0.09) | -1.98(-2.31,-1.66) |
| Senegal | 38.09(21.19,55.02) | 1.04(0.58,1.51) | 18.28(12.84,25.64) | 0.29(0.20,0.40) | -3.65(-3.97,-3.32) |
| Greece | 0.20(0.19,0.22) | 0.01(0.01,0.01) | 0.18(0.15,0.22) | 0.01(0.01,0.02) | 1.67(1.29,2.06) |
| Niger | 60.47(25.12,90.39) | 1.49(0.62,2.22) | 48.21(28.26,70.91) | 0.38(0.22,0.56) | -4.62(-4.93,-4.31) |
| Lithuania | 0.86(0.79,0.94) | 0.10(0.10,0.11) | 0.17(0.14,0.23) | 0.04(0.03,0.06) | -3.07(-4.09,-2.03) |
| Kiribati | 0.12(0.08,0.17) | 0.40(0.26,0.57) | 0.07(0.04,0.12) | 0.16(0.10,0.28) | -2.91(-3.06,-2.76) |
| Maldives | 0.37(0.24,0.48) | 0.35(0.23,0.46) | 0.10(0.08,0.14) | 0.10(0.08,0.14) | -3.31(-3.70,-2.92) |
| Comoros | 2.16(1.20,3.38) | 1.01(0.56,1.59) | 1.30(0.88,1.91) | 0.54(0.37,0.80) | -2.20(-2.47,-1.93) |
| Switzerland | 0.19(0.16,0.22) | 0.02(0.01,0.02) | 0.18(0.15,0.22) | 0.01(0.01,0.02) | -0.06(-0.36,0.25) |
| United Republic of Tanzania | 168.01(90.13,260.63) | 1.39(0.75,2.16) | 151.97(102.15,223.89) | 0.62(0.42,0.92) | -2.03(-2.24,-1.81) |
| Barbados | 0.10(0.08,0.12) | 0.16(0.13,0.19) | 0.12(0.09,0.17) | 0.26(0.19,0.35) | 2.55(1.64,3.48) |
| Georgia | 0.64(0.57,0.72) | 0.05(0.04,0.05) | 0.13(0.11,0.15) | 0.02(0.01,0.02) | -1.08(-2.32,0.16) |
| Panama | 1.25(1.06,1.44) | 0.15(0.13,0.17) | 2.26(1.78,2.87) | 0.20(0.15,0.25) | 1.73(1.37,2.10) |
| Nauru | 0.01(0.00,0.01) | 0.14(0.09,0.20) | 0.00(0.00,0.01) | 0.10(0.07,0.16) | -1.07(-1.75,-0.39) |
| Niue | 0.00(0.00,0.00) | 0.08(0.05,0.11) | 0.00(0.00,0.00) | 0.28(0.22,0.37) | 1.18(0.16,2.21) |
| Belize | 0.13(0.11,0.15) | 0.15(0.13,0.18) | 0.22(0.18,0.27) | 0.18(0.15,0.22) | 1.30(0.45,2.17) |
| Latvia | 1.07(0.96,1.20) | 0.19(0.17,0.21) | 0.13(0.11,0.16) | 0.04(0.04,0.05) | -4.14(-5.09,-3.18) |
| Ethiopia | 493.53(261.42,727.05) | 2.03(1.07,2.98) | 269.54(186.10,369.14) | 0.61(0.42,0.83) | -4.08(-4.35,-3.81) |
| Angola | 21.34(7.91,33.65) | 0.45(0.17,0.71) | 23.02(14.47,34.67) | 0.15(0.09,0.23) | -3.48(-3.81,-3.14) |
| Kazakhstan | 10.78(9.23,12.79) | 0.21(0.18,0.25) | 11.90(9.84,14.59) | 0.22(0.18,0.27) | 0.08(-0.39,0.54) |
| Croatia | 1.10(1.00,1.22) | 0.11(0.10,0.12) | 0.24(0.20,0.29) | 0.04(0.03,0.05) | -2.31(-3.77,-0.82) |
| Czechia | 2.89(2.63,3.14) | 0.13(0.12,0.14) | 0.69(0.58,0.86) | 0.04(0.03,0.05) | -3.06(-4.34,-1.76) |
| Papua New Guinea | 3.38(1.58,5.60) | 0.20(0.09,0.33) | 6.21(3.40,9.62) | 0.16(0.09,0.25) | -0.58(-0.76,-0.39) |
| Mongolia | 2.61(1.85,3.58) | 0.29(0.21,0.40) | 1.04(0.77,1.42) | 0.10(0.07,0.13) | -3.30(-3.89,-2.70) |
| New Zealand | 1.09(0.99,1.19) | 0.14(0.12,0.15) | 0.49(0.42,0.56) | 0.05(0.04,0.06) | -1.16(-2.32,0.01) |
| Trinidad and Tobago | 0.28(0.23,0.33) | 0.07(0.06,0.08) | 0.35(0.26,0.47) | 0.13(0.10,0.17) | 2.82(1.70,3.95) |
| Kenya | 62.57(40.16,84.52) | 0.56(0.36,0.76) | 47.69(33.93,80.42) | 0.26(0.18,0.43) | -1.44(-1.85,-1.03) |
| Nicaragua | 5.13(3.85,6.63) | 0.28(0.21,0.36) | 1.64(1.10,2.49) | 0.08(0.06,0.13) | -2.99(-3.36,-2.63) |
| Iraq | 5.19(3.40,8.10) | 0.06(0.04,0.10) | 2.48(1.70,3.35) | 0.02(0.01,0.02) | -3.45(-3.72,-3.17) |
| Ireland | 0.22(0.20,0.25) | 0.02(0.02,0.03) | 0.16(0.14,0.19) | 0.02(0.01,0.02) | -0.13(-0.99,0.74) |
| Jordan | 1.10(0.86,1.39) | 0.07(0.05,0.09) | 1.27(0.96,1.67) | 0.04(0.03,0.05) | -1.94(-2.13,-1.75) |
| United Kingdom | 2.12(2.05,2.19) | 0.02(0.02,0.02) | 4.46(3.92,5.13) | 0.04(0.03,0.04) | 2.32(0.66,4.01) |
| Iceland | 0.00(0.00,0.00) | 0.01(0.00,0.01) | 0.01(0.01,0.01) | 0.01(0.01,0.01) | 2.20(1.15,3.26) |
| Cuba | 0.38(0.33,0.42) | 0.02(0.01,0.02) | 0.51(0.42,0.61) | 0.03(0.02,0.03) | 3.34(2.74,3.95) |
| Botswana | 0.19(0.10,0.30) | 0.03(0.02,0.05) | 0.31(0.17,0.50) | 0.05(0.02,0.07) | 1.73(1.43,2.04) |
| Turkey | 46.20(30.87,64.69) | 0.23(0.15,0.32) | 9.67(7.11,12.80) | 0.05(0.04,0.07) | -4.26(-4.44,-4.08) |
| Hungary | 2.10(1.87,2.32) | 0.10(0.09,0.11) | 0.54(0.42,0.69) | 0.04(0.03,0.05) | -1.99(-3.46,-0.50) |
| Bolivia (Plurinational State of) | 27.23(21.19,34.92) | 1.01(0.79,1.30) | 13.67(9.20,19.42) | 0.39(0.26,0.56) | -2.69(-2.81,-2.57) |
| United Arab Emirates | 0.62(0.45,0.88) | 0.10(0.08,0.15) | 0.38(0.28,0.54) | 0.03(0.02,0.04) | -2.73(-3.21,-2.25) |
| Argentina | 4.91(4.52,5.30) | 0.05(0.04,0.05) | 11.13(9.50,12.98) | 0.11(0.09,0.13) | 3.32(2.54,4.12) |
| Taiwan (Province of China) | 4.60(4.22,4.98) | 0.08(0.08,0.09) | 2.58(2.26,2.98) | 0.09(0.08,0.10) | 1.25(0.35,2.16) |
| Malaysia | 5.66(3.86,7.73) | 0.09(0.06,0.12) | 3.87(2.88,5.13) | 0.05(0.04,0.07) | -1.11(-1.50,-0.71) |

**Supplementary Table 6**. DALYs of UTIs in children, by countries and territories, 1990-2021

| Location | Rate per 100 000 (95% UI) | | | | |
| --- | --- | --- | --- | --- | --- |
|  | 1990 | | 2021 | | 1990-2021 |
|  | Cases​ | Rate | Cases​ | Rate | EAPC* |
| Venezuela (Bolivarian Republic of) | 1,159.29(1,003.91, 1,375.46) | 16.34(14.15,19.39) | 1,234.25(949.94, 1,609.56) | 18.63(14.34,24.30) | 1.12(0.67,1.58) |
| Russian Federation | 5,641.10(4,948.26, 6,548.24) | 16.26(14.26,18.87) | 3,195.64(2,476.38, 4,115.09) | 12.25(9.50,15.78) | -1.09(-1.54,-0.64) |
| Yemen | 1,547.13(870.45, 2,363.26) | 21.81(12.27,33.31) | 1,698.58(1,016.80, 2,428.16) | 12.32(7.37,17.61) | -1.58(-1.89,-1.27) |
| Central African Republic | 479.41(227.22, 729.87) | 39.21(18.58,59.69) | 537.83(320.53, 774.61) | 23.55(14.03,33.92) | -1.35(-1.50,-1.21) |
| Italy | 250.33(204.15, 311.13) | 2.71(2.21,3.37) | 184.77(143.55, 238.44) | 2.43(1.89,3.14) | 0.32(0.14,0.51) |
| Samoa | 8.58(5.71, 12.70) | 12.04(8.01,17.83) | 4.54(2.99, 7.05) | 5.68(3.74,8.81) | -2.28(-2.36,-2.20) |
| Tajikistan | 1,540.35(1,176.28, 1,993.41) | 66.34(50.66,85.85) | 1,459.65(991.53, 2,453.88) | 40.72(27.66,68.46) | -1.89(-2.22,-1.56) |
| Andorra | 0.63(0.43, 0.85) | 6.63(4.52,8.96) | 0.24(0.17, 0.32) | 2.32(1.65,3.13) | -3.26(-3.48,-3.04) |
| Madagascar | 3,924.98(2,071.46, 5,962.27) | 71.94(37.97,109.28) | 2,986.04(2,015.13, 4,486.06) | 25.45(17.17,38.23) | -2.88(-3.04,-2.71) |
| Poland | 913.88(765.37, 1,106.35) | 9.54(7.99,11.55) | 337.71(279.69, 416.07) | 5.74(4.75,7.07) | -1.72(-2.58,-0.84) |
| Puerto Rico | 84.64(69.05, 106.63) | 8.50(6.93,10.71) | 43.19(34.12, 54.18) | 9.72(7.68,12.19) | 0.89(0.30,1.50) |
| Gambia | 280.31(163.75, 407.84) | 60.77(35.50,88.42) | 239.49(156.89, 340.46) | 24.11(15.79,34.27) | -3.18(-3.49,-2.86) |
| Montenegro | 5.81(3.78, 8.74) | 3.59(2.34,5.41) | 3.54(2.22, 5.41) | 3.17(1.99,4.86) | -0.38(-0.44,-0.33) |
| Austria | 33.57(24.28, 49.17) | 2.49(1.80,3.65) | 31.84(22.21, 47.61) | 2.45(1.71,3.67) | 0.36(-0.05,0.76) |
| Ukraine | 2,027.74(1,589.39, 2,586.50) | 17.83(13.97,22.74) | 800.91(589.90, 1,062.50) | 12.62(9.30,16.74) | -0.75(-1.43,-0.05) |
| Kuwait | 17.52(11.87, 24.30) | 3.16(2.14,4.38) | 85.48(70.77, 103.68) | 10.11(8.37,12.26) | 3.53(2.49,4.58) |
| Lesotho | 25.62(15.97, 36.31) | 3.75(2.34,5.32) | 33.34(20.39, 48.64) | 5.29(3.23,7.71) | 1.79(1.53,2.05) |
| Togo | 1,153.26(612.34, 1,654.45) | 65.43(34.74,93.87) | 810.68(513.88, 1,158.88) | 24.50(15.53,35.02) | -2.86(-3.03,-2.70) |
| Philippines | 12,840.54(7,792.66, 16,211.48) | 50.93(30.91,64.29) | 9,063.58(6,460.53, 11,385.73) | 26.66(19.00,33.49) | -1.39(-1.60,-1.18) |
| Bermuda | 0.58(0.41, 0.84) | 4.84(3.41,7.03) | 0.52(0.38, 0.72) | 6.21(4.52,8.50) | 1.06(0.82,1.31) |
| Ecuador | 486.22(431.39, 553.59) | 12.58(11.16,14.32) | 924.64(715.74, 1,197.34) | 18.23(14.11,23.61) | 1.32(0.40,2.25) |
| Sierra Leone | 2,645.50(1,085.86, 3,979.16) | 145.95(59.91,219.52) | 1,736.94(962.44, 2,552.61) | 48.57(26.91,71.38) | -3.55(-3.74,-3.36) |
| Chile | 282.96(252.59, 327.42) | 7.12(6.36,8.24) | 329.45(280.78, 399.41) | 9.02(7.69,10.94) | 1.35(0.88,1.83) |
| Lebanon | 277.68(203.09, 363.68) | 26.55(19.42,34.77) | 126.42(91.16, 166.20) | 9.89(7.13,13.00) | -3.16(-3.45,-2.87) |
| Côte d'Ivoire | 4,164.56(2,517.09, 5,798.53) | 73.02(44.13,101.67) | 4,089.51(2,701.70, 5,705.51) | 35.34(23.35,49.31) | -1.98(-2.24,-1.71) |
| Dominican Republic | 301.14(227.50, 404.76) | 11.17(8.44,15.02) | 173.72(115.79, 272.30) | 5.91(3.94,9.27) | -1.68(-1.91,-1.45) |
| Peru | 6,460.58(5,297.26, 7,966.12) | 77.83(63.82,95.97) | 2,688.03(1,785.40, 3,686.89) | 28.18(18.72,38.66) | -2.65(-2.94,-2.35) |
| Brazil | 28,041.93(25,010.23, 31,070.76) | 53.98(48.15,59.82) | 17,765.28(14,534.10, 21,178.39) | 36.87(30.16,43.95) | -0.16(-0.52,0.21) |
| American Samoa | 3.93(2.95, 5.17) | 20.68(15.52,27.19) | 1.28(0.88, 1.85) | 9.02(6.17,13.05) | -2.74(-2.95,-2.52) |
| Democratic Republic of the Congo | 5,787.48(2,703.72, 8,699.23) | 32.69(15.27,49.14) | 3,674.59(2,482.09, 5,224.26) | 9.67(6.53,13.75) | -3.50(-3.77,-3.23) |
| Libya | 209.23(155.58, 281.26) | 11.55(8.59,15.53) | 182.38(114.07, 268.93) | 12.23(7.65,18.03) | 0.91(0.69,1.14) |
| Dominica | 1.32(0.97, 1.82) | 5.32(3.91,7.32) | 1.48(0.96, 2.06) | 10.84(7.01,15.04) | 2.75(2.50,2.99) |
| Saint Kitts and Nevis | 1.82(1.55, 2.15) | 12.86(11.01,15.26) | 2.32(1.86, 2.87) | 23.57(18.84,29.11) | 2.72(2.14,3.30) |
| Malawi | 3,467.45(1,675.26, 5,117.38) | 76.21(36.82,112.48) | 1,800.83(1,276.25, 2,619.05) | 22.17(15.71,32.24) | -3.78(-4.00,-3.56) |
| Myanmar | 7,177.94(3,365.95, 10,176.44) | 48.58(22.78,68.87) | 3,629.57(2,588.98, 4,813.70) | 23.24(16.58,30.83) | -2.54(-2.82,-2.27) |
| Afghanistan | 1,751.18(1,096.42, 2,480.22) | 40.65(25.45,57.57) | 3,414.09(2,102.52, 4,938.82) | 24.04(14.81,34.78) | -1.37(-1.59,-1.16) |
| Congo | 273.64(147.45, 407.72) | 25.99(14.00,38.72) | 186.01(126.59, 261.52) | 9.64(6.56,13.56) | -3.36(-3.82,-2.90) |
| Luxembourg | 1.09(0.75, 1.55) | 1.65(1.14,2.35) | 1.57(1.05, 2.22) | 1.55(1.04,2.19) | -0.17(-0.38,0.03) |
| Mauritius | 6.44(5.64, 7.53) | 1.95(1.71,2.28) | 12.21(10.19, 15.39) | 5.89(4.91,7.42) | 4.11(3.52,4.70) |
| Guinea | 3,537.95(1,578.69, 5,169.56) | 128.57(57.37,187.86) | 2,620.08(1,564.21, 3,718.62) | 43.34(25.87,61.51) | -2.87(-3.05,-2.69) |
| Paraguay | 239.45(161.65, 334.66) | 14.34(9.68,20.04) | 276.66(190.74, 388.82) | 13.78(9.50,19.37) | -0.18(-0.41,0.05) |
| South Africa | 745.66(508.01, 1,008.31) | 5.48(3.73,7.41) | 553.29(425.91, 754.84) | 3.64(2.80,4.96) | -1.18(-1.39,-0.98) |
| Thailand | 2,910.19(1,754.05, 4,992.56) | 17.26(10.40,29.61) | 1,615.42(1,315.44, 1,977.78) | 16.54(13.47,20.25) | 0.18(-0.02,0.39) |
| Uruguay | 44.79(38.59, 53.38) | 5.47(4.71,6.52) | 61.88(50.32, 74.37) | 9.38(7.63,11.28) | 1.72(0.98,2.46) |
| Turkmenistan | 515.01(448.89, 595.02) | 34.31(29.91,39.65) | 930.60(767.83, 1,123.17) | 61.06(50.38,73.70) | 2.10(1.68,2.53) |
| Belgium | 47.27(37.78, 60.99) | 2.62(2.09,3.38) | 54.23(42.43, 69.75) | 2.84(2.22,3.65) | 0.59(0.17,1.02) |
| Solomon Islands | 19.55(12.45, 29.80) | 12.56(7.99,19.14) | 17.32(11.64, 29.28) | 6.66(4.48,11.26) | -2.13(-2.38,-1.87) |
| Morocco | 2,476.89(1,539.74, 3,479.58) | 25.31(15.73,35.56) | 1,020.78(698.07, 1,378.16) | 10.42(7.13,14.07) | -2.37(-2.81,-1.93) |
| Namibia | 22.10(13.72, 33.08) | 3.68(2.28,5.51) | 28.07(16.86, 40.40) | 3.40(2.04,4.89) | 0.19(-0.04,0.43) |
| Grenada | 2.86(2.26, 3.55) | 8.55(6.77,10.63) | 2.52(2.01, 3.14) | 11.56(9.19,14.38) | 1.60(1.24,1.97) |
| Mozambique | 9,911.15(5,039.29, 16,511.02) | 159.75(81.22,266.13) | 6,941.92(4,464.60, 11,719.27) | 48.66(31.30,82.15) | -3.34(-3.56,-3.12) |
| Eswatini | 23.67(14.68, 33.46) | 6.14(3.81,8.67) | 23.00(13.86, 32.64) | 5.57(3.36,7.91) | -0.01(-0.13,0.11) |
| San Marino | 0.08(0.05, 0.11) | 1.88(1.24,2.68) | 0.06(0.04, 0.09) | 1.37(0.84,2.11) | -0.81(-0.87,-0.74) |
| Rwanda | 4,381.18(2,450.89, 7,309.71) | 129.13(72.24,215.44) | 1,961.89(1,278.44, 2,883.34) | 39.47(25.72,58.01) | -4.39(-4.77,-4.00) |
| Guam | 4.77(3.64, 6.08) | 11.43(8.73,14.58) | 3.63(2.58, 4.84) | 9.93(7.05,13.22) | 0.27(-0.16,0.70) |
| Democratic People's Republic of Korea | 506.15(322.50, 751.63) | 8.51(5.42,12.63) | 142.02(80.90, 274.33) | 2.98(1.69,5.75) | -3.18(-3.41,-2.94) |
| Equatorial Guinea | 58.93(28.80, 90.24) | 29.92(14.63,45.83) | 70.86(41.86, 110.72) | 12.11(7.15,18.93) | -3.16(-3.36,-2.95) |
| Serbia | 100.70(70.00, 141.64) | 4.64(3.23,6.53) | 35.89(23.65, 52.16) | 2.70(1.78,3.93) | -1.58(-1.75,-1.40) |
| Gabon | 77.40(44.83, 114.75) | 18.99(11.00,28.16) | 70.36(44.74, 105.64) | 11.01(7.00,16.53) | -1.17(-1.58,-0.75) |
| Republic of Korea | 631.69(444.91, 881.98) | 5.56(3.91,7.76) | 242.19(159.80, 356.99) | 3.99(2.63,5.88) | -0.90(-1.06,-0.75) |
| Bosnia and Herzegovina | 59.23(42.77, 81.96) | 5.41(3.90,7.48) | 20.57(13.56, 30.30) | 4.19(2.76,6.18) | -0.98(-1.27,-0.69) |
| Romania | 882.31(771.41, 1,000.39) | 15.85(13.85,17.97) | 243.00(182.71, 327.24) | 8.07(6.07,10.87) | -2.19(-2.95,-1.43) |
| Liberia | 1,887.14(902.12, 2,715.06) | 166.98(79.82,240.23) | 702.46(419.92, 1,014.29) | 32.14(19.21,46.40) | -4.68(-5.41,-3.95) |
| Cook Islands | 0.06(0.04, 0.08) | 0.93(0.62,1.26) | 0.02(0.01, 0.03) | 0.51(0.33,0.78) | -3.34(-3.81,-2.86) |
| Malta | 2.78(2.35, 3.38) | 3.17(2.68,3.87) | 2.62(2.07, 3.15) | 4.09(3.24,4.92) | 1.46(1.15,1.78) |
| Timor-Leste | 138.36(61.48, 199.04) | 41.60(18.48,59.84) | 78.97(57.53, 103.50) | 15.17(11.05,19.88) | -3.57(-3.89,-3.25) |
| Tonga | 4.66(3.17, 6.76) | 11.14(7.58,16.17) | 2.90(1.83, 4.83) | 7.44(4.70,12.39) | -1.26(-1.57,-0.94) |
| Tuvalu | 0.92(0.48, 1.40) | 26.45(13.75,40.38) | 0.23(0.15, 0.35) | 6.24(4.09,9.28) | -4.42(-4.70,-4.14) |
| Sri Lanka | 690.53(499.96, 897.27) | 12.48(9.04,16.22) | 352.51(229.51, 488.43) | 6.91(4.50,9.57) | -1.40(-1.96,-0.84) |
| Uzbekistan | 1,870.45(1,524.33, 2,325.50) | 21.86(17.82,27.18) | 1,852.13(1,518.84, 2,262.64) | 18.35(15.05,22.42) | 1.33(0.28,2.39) |
| Cyprus | 18.30(14.18, 24.05) | 9.25(7.16,12.15) | 5.97(4.52, 7.80) | 2.73(2.07,3.57) | -3.45(-3.81,-3.09) |
| Guinea-Bissau | 548.01(280.19, 822.88) | 113.60(58.08,170.58) | 257.45(169.89, 366.37) | 28.66(18.92,40.79) | -4.01(-4.38,-3.64) |
| Canada | 268.26(219.63, 336.06) | 4.66(3.82,5.84) | 212.75(159.08, 290.71) | 3.45(2.58,4.71) | -0.61(-0.79,-0.43) |
| Ghana | 3,764.12(1,987.34, 5,602.07) | 56.04(29.59,83.41) | 4,107.21(2,473.36, 7,486.72) | 31.88(19.20,58.11) | -1.08(-1.33,-0.83) |
| Brunei Darussalam | 10.68(7.93, 15.31) | 11.79(8.75,16.91) | 10.69(7.96, 14.19) | 11.31(8.41,15.00) | 0.75(0.42,1.09) |
| Netherlands | 122.02(105.27, 141.10) | 4.48(3.86,5.18) | 128.66(107.75, 151.47) | 4.80(4.02,5.65) | 0.50(0.16,0.84) |
| France | 275.66(215.75, 359.64) | 2.35(1.84,3.07) | 299.22(236.41, 388.18) | 2.58(2.04,3.34) | 0.57(0.32,0.82) |
| Viet Nam | 502.50(362.68, 791.34) | 1.90(1.37,2.98) | 351.23(261.31, 474.35) | 1.42(1.06,1.92) | -0.69(-0.79,-0.58) |
| Zimbabwe | 176.86(104.70, 242.82) | 3.67(2.17,5.04) | 352.51(191.66, 525.42) | 5.60(3.05,8.35) | 2.20(1.83,2.58) |
| United States Virgin Islands | 3.94(2.91, 5.49) | 12.33(9.12,17.17) | 0.98(0.66, 1.41) | 7.34(4.93,10.54) | -1.18(-1.48,-0.88) |
| Guatemala | 1,435.39(1,261.16, 1,633.69) | 35.34(31.05,40.23) | 1,434.90(1,127.91, 1,815.21) | 29.08(22.86,36.79) | 0.42(-0.10,0.94) |
| Japan | 1,493.02(1,070.97, 2,073.95) | 6.47(4.64,8.98) | 980.63(709.91, 1,341.89) | 6.35(4.60,8.69) | 0.46(0.32,0.61) |
| Bangladesh | 66,472.55(36,543.21, 88,488.91) | 135.90(74.71,180.92) | 18,380.47(14,099.84, 23,858.29) | 40.16(30.81,52.13) | -3.60(-3.76,-3.44) |
| Bahrain | 5.91(3.99, 8.53) | 3.62(2.45,5.23) | 9.33(6.18, 14.37) | 3.14(2.08,4.84) | -0.87(-1.32,-0.41) |
| Colombia | 3,817.09(3,289.08, 4,461.01) | 32.73(28.20,38.25) | 2,607.11(2,010.11, 3,352.12) | 24.57(18.94,31.59) | 0.07(-0.25,0.39) |
| China | 32,984.05(20,004.76, 41,195.89) | 10.36(6.28,12.94) | 3,686.97(3,002.83, 4,805.54) | 1.42(1.16,1.85) | -6.86(-7.16,-6.56) |
| Greenland | 1.51(1.09, 2.31) | 10.62(7.64,16.25) | 0.57(0.39, 0.92) | 4.88(3.34,7.81) | -2.67(-2.88,-2.46) |
| Sudan | 3,042.03(1,566.53, 4,655.35) | 34.21(17.62,52.35) | 2,742.56(1,684.17, 3,948.97) | 16.53(10.15,23.80) | -1.93(-2.26,-1.61) |
| Saint Lucia | 4.41(3.55, 5.58) | 8.55(6.89,10.83) | 4.10(3.13, 5.22) | 13.81(10.55,17.57) | 2.08(1.61,2.56) |
| Palestine | 68.11(43.55, 103.92) | 7.03(4.50,10.73) | 74.89(51.07, 101.16) | 4.01(2.74,5.42) | -1.37(-1.54,-1.19) |
| Portugal | 105.04(92.51, 120.54) | 4.96(4.37,5.70) | 49.06(39.81, 60.33) | 3.60(2.92,4.43) | -1.12(-1.41,-0.84) |
| Spain | 285.69(241.04, 345.80) | 3.65(3.08,4.41) | 234.99(194.67, 292.98) | 3.63(3.00,4.52) | 0.17(-0.22,0.56) |
| Guyana | 40.20(33.27, 48.81) | 13.68(11.32,16.61) | 45.47(34.55, 58.38) | 21.31(16.19,27.36) | 2.52(1.88,3.17) |
| Vanuatu | 6.34(4.18, 9.55) | 9.31(6.14,14.03) | 6.71(4.70, 9.75) | 5.76(4.03,8.36) | -1.58(-2.05,-1.11) |
| Armenia | 241.84(195.54, 300.69) | 23.18(18.74,28.82) | 94.47(75.02, 118.82) | 15.95(12.66,20.06) | 0.58(-0.36,1.53) |
| Tokelau | 0.04(0.02, 0.06) | 6.24(3.98,10.34) | 0.08(0.05, 0.14) | 19.30(12.78,35.64) | -0.76(-2.52,1.03) |
| Albania | 89.17(50.94, 138.43) | 7.98(4.56,12.39) | 14.80(9.92, 21.18) | 3.33(2.23,4.77) | -2.66(-3.07,-2.26) |
| Saudi Arabia | 2,216.07(1,530.81, 2,993.16) | 33.82(23.36,45.67) | 545.13(393.90, 790.19) | 7.21(5.21,10.44) | -5.27(-5.52,-5.02) |
| Algeria | 2,142.52(1,516.76, 2,834.47) | 19.98(14.14,26.43) | 1,318.10(973.07, 1,749.74) | 9.91(7.32,13.15) | -1.35(-1.67,-1.03) |
| Egypt | 805.30(530.76, 1,163.60) | 3.63(2.39,5.25) | 1,130.35(798.65, 1,558.92) | 3.07(2.17,4.23) | 0.48(0.10,0.86) |
| Antigua and Barbuda | 1.27(0.98, 1.66) | 6.99(5.40,9.15) | 2.46(2.09, 2.94) | 14.58(12.38,17.39) | 2.65(2.08,3.22) |
| Mexico | 14,287.24(12,915.01, 16,084.10) | 42.76(38.65,48.13) | 9,204.46(7,411.48, 11,631.74) | 28.70(23.11,36.27) | -0.44(-0.95,0.08) |
| Cambodia | 1,761.45(1,013.39, 2,376.91) | 37.79(21.74,51.00) | 752.40(569.32, 957.22) | 14.71(11.13,18.71) | -3.31(-3.47,-3.16) |
| United States of America | 3,907.52(3,473.21, 4,476.23) | 6.99(6.21,8.01) | 2,996.31(2,554.06, 3,567.90) | 5.04(4.30,6.00) | -1.00(-1.18,-0.83) |
| India | 297,897.42(202,235.76, 382,053.96) | 91.23(61.94,117.01) | 160,543.21(98,414.73, 243,629.54) | 43.82(26.86,66.49) | -2.20(-2.33,-2.07) |
| Norway | 23.25(18.25, 30.18) | 2.91(2.29,3.78) | 19.21(13.54, 26.53) | 2.08(1.47,2.87) | -0.32(-0.73,0.08) |
| Singapore | 103.23(90.94, 117.20) | 15.90(14.00,18.05) | 94.96(79.05, 117.88) | 11.69(9.73,14.52) | -0.21(-0.63,0.20) |
| Suriname | 38.72(30.16, 52.51) | 29.72(23.15,40.31) | 48.34(31.14, 66.66) | 33.74(21.73,46.53) | 0.47(0.17,0.78) |
| Iran (Islamic Republic of) | 9,702.98(5,480.32, 12,518.45) | 38.22(21.59,49.32) | 1,273.03(1,025.50, 1,553.29) | 6.31(5.08,7.70) | -3.83(-4.65,-2.99) |
| Northern Mariana Islands | 1.43(1.03, 2.07) | 11.79(8.48,17.04) | 0.57(0.42, 0.80) | 5.07(3.71,7.07) | -2.86(-3.39,-2.33) |
| Finland | 27.76(21.56, 36.74) | 2.88(2.23,3.81) | 25.97(20.40, 34.65) | 3.07(2.41,4.09) | 0.29(-0.12,0.71) |
| Slovakia | 117.36(92.51, 148.83) | 8.85(6.98,11.23) | 48.23(35.54, 65.22) | 5.63(4.15,7.61) | -1.15(-1.35,-0.95) |
| Slovenia | 23.76(18.32, 31.62) | 5.75(4.43,7.65) | 25.50(17.22, 37.41) | 8.17(5.52,11.98) | 1.27(0.52,2.02) |
| Eritrea | 1,050.09(577.41, 1,642.77) | 65.97(36.27,103.20) | 870.17(523.40, 1,459.79) | 34.47(20.73,57.82) | -2.00(-2.19,-1.81) |
| El Salvador | 1,307.11(1,030.48, 1,642.63) | 60.57(47.75,76.12) | 375.53(279.39, 515.20) | 20.65(15.36,28.33) | -3.02(-3.43,-2.61) |
| North Macedonia | 21.68(14.88, 29.59) | 4.12(2.82,5.62) | 8.98(5.55, 13.59) | 2.74(1.70,4.15) | -0.96(-1.22,-0.70) |
| Denmark | 31.37(25.77, 38.66) | 3.55(2.92,4.38) | 28.78(22.40, 36.83) | 3.02(2.35,3.86) | -0.25(-0.53,0.03) |
| Sao Tome and Principe | 58.29(39.43, 79.78) | 102.87(69.58,140.79) | 16.47(9.05, 41.35) | 21.16(11.63,53.13) | -4.88(-5.39,-4.37) |
| Republic of Moldova | 279.73(243.81, 327.94) | 22.63(19.73,26.53) | 63.35(49.46, 80.57) | 12.13(9.47,15.42) | -1.73(-2.59,-0.86) |
| Zambia | 3,685.27(1,858.09, 5,568.49) | 98.16(49.49,148.31) | 2,700.42(1,763.86, 4,325.56) | 32.65(21.32,52.29) | -3.27(-3.44,-3.10) |
| Sweden | 73.16(62.26, 86.83) | 4.74(4.03,5.62) | 45.82(32.78, 63.22) | 2.52(1.80,3.47) | -1.55(-1.83,-1.27) |
| Qatar | 4.58(2.62, 6.86) | 3.66(2.10,5.49) | 13.19(8.23, 20.09) | 2.67(1.67,4.07) | -1.58(-1.92,-1.25) |
| Israel | 88.80(78.59, 101.28) | 5.79(5.13,6.61) | 103.60(85.55, 126.62) | 3.94(3.26,4.82) | -0.33(-0.73,0.08) |
| Cabo Verde | 33.05(15.48, 47.68) | 21.01(9.84,30.30) | 9.16(6.45, 14.03) | 6.40(4.51,9.80) | -4.07(-4.32,-3.82) |
| Costa Rica | 157.11(130.21, 190.44) | 13.98(11.58,16.94) | 141.34(115.20, 173.48) | 13.89(11.32,17.05) | 0.62(0.30,0.94) |
| Cameroon | 4,973.92(2,917.24, 6,896.99) | 101.88(59.75,141.27) | 5,991.44(3,762.28, 8,534.25) | 44.49(27.94,63.37) | -2.18(-2.40,-1.96) |
| Haiti | 1,601.85(705.11, 2,254.44) | 59.04(25.99,83.10) | 1,967.39(927.06, 3,135.63) | 45.20(21.30,72.04) | -0.29(-0.51,-0.07) |
| Micronesia (Federated States of) | 5.78(4.03, 8.10) | 12.59(8.77,17.63) | 1.58(1.06, 2.56) | 5.16(3.48,8.36) | -3.03(-3.21,-2.85) |
| Burundi | 2,714.16(1,312.00, 4,459.40) | 103.55(50.05,170.13) | 1,738.16(991.06, 3,019.68) | 29.69(16.93,51.58) | -3.23(-3.63,-2.82) |
| Honduras | 1,736.34(1,387.85, 2,163.89) | 78.59(62.81,97.94) | 750.72(440.69, 1,395.31) | 22.91(13.45,42.58) | -3.83(-3.88,-3.77) |
| Somalia | 3,761.17(1,930.51, 6,322.70) | 96.55(49.56,162.30) | 4,161.03(2,236.88, 6,606.38) | 40.28(21.66,63.96) | -2.21(-2.63,-1.78) |
| Pakistan | 61,852.38(45,014.30, 82,992.49) | 125.61(91.41,168.54) | 92,161.64(68,738.66, 124,985.36) | 107.86(80.45,146.28) | 0.51(0.17,0.86) |
| Bhutan | 278.67(141.56, 430.79) | 106.28(53.99,164.30) | 103.00(66.06, 154.98) | 55.03(35.29,82.80) | -2.40(-2.73,-2.08) |
| Jamaica | 98.53(82.84, 119.07) | 11.80(9.92,14.26) | 79.93(61.73, 102.06) | 13.69(10.57,17.48) | 1.05(0.62,1.48) |
| Oman | 186.03(127.68, 246.36) | 22.14(15.19,29.32) | 129.33(102.72, 160.41) | 10.57(8.40,13.12) | -1.01(-1.65,-0.36) |
| Syrian Arab Republic | 4,334.93(2,916.12, 5,753.90) | 73.20(49.24,97.16) | 877.73(663.54, 1,226.46) | 23.96(18.11,33.48) | -3.22(-3.91,-2.52) |
| Djibouti | 98.80(54.98, 153.20) | 56.74(31.58,87.99) | 125.68(77.13, 192.73) | 30.42(18.67,46.65) | -1.92(-2.41,-1.43) |
| Kyrgyzstan | 458.43(401.54, 521.83) | 27.33(23.94,31.11) | 337.06(297.58, 382.28) | 14.82(13.08,16.81) | -0.47(-1.36,0.41) |
| Nigeria | 32,177.89(15,925.30, 42,957.64) | 82.24(40.70,109.80) | 45,632.83(25,862.11, 64,761.23) | 44.92(25.46,63.75) | -1.68(-1.81,-1.55) |
| Estonia | 67.42(59.36, 78.01) | 19.31(17.00,22.35) | 17.16(13.14, 22.85) | 7.94(6.08,10.57) | -2.31(-2.92,-1.70) |
| Tunisia | 556.81(386.53, 736.24) | 17.93(12.45,23.71) | 185.17(140.13, 238.58) | 6.70(5.07,8.63) | -2.65(-2.84,-2.47) |
| Benin | 2,652.09(1,428.37, 3,795.45) | 109.51(58.98,156.72) | 3,072.13(1,922.73, 4,455.27) | 50.53(31.62,73.27) | -2.14(-2.33,-1.96) |
| Mali | 4,731.18(1,840.02, 7,119.61) | 114.56(44.55,172.40) | 4,964.57(2,486.72, 7,254.26) | 42.89(21.48,62.67) | -2.94(-3.07,-2.81) |
| Belarus | 349.90(291.44, 420.64) | 14.56(12.12,17.50) | 139.22(107.91, 180.52) | 8.82(6.84,11.44) | -1.28(-1.74,-0.81) |
| Lao People's Democratic Republic | 885.12(469.09, 1,246.56) | 48.02(25.45,67.63) | 431.26(303.14, 601.72) | 18.78(13.20,26.20) | -3.19(-3.33,-3.04) |
| Uganda | 6,278.16(3,356.30, 9,982.31) | 74.57(39.86,118.56) | 8,049.83(4,989.01, 12,071.98) | 40.58(25.15,60.86) | -1.58(-1.81,-1.35) |
| Bahamas | 8.56(6.97, 10.71) | 10.61(8.64,13.27) | 13.80(10.64, 17.63) | 16.99(13.10,21.71) | 1.69(1.05,2.32) |
| Saint Vincent and the Grenadines | 5.62(4.64, 6.78) | 13.67(11.30,16.50) | 5.54(4.50, 6.81) | 22.19(18.04,27.30) | 1.57(1.15,1.98) |
| Germany | 326.34(254.88, 438.67) | 2.52(1.97,3.39) | 302.43(233.31, 408.04) | 2.53(1.95,3.41) | 0.64(0.31,0.97) |
| Azerbaijan | 728.61(579.23, 898.43) | 30.02(23.87,37.02) | 511.32(393.01, 644.73) | 21.66(16.65,27.31) | -1.22(-1.61,-0.83) |
| Chad | 2,261.33(955.24, 3,335.97) | 77.27(32.64,114.00) | 4,249.39(1,803.51, 6,609.01) | 47.14(20.01,73.31) | -1.53(-1.60,-1.46) |
| Bulgaria | 338.18(294.50, 383.69) | 19.48(16.96,22.10) | 109.38(86.79, 136.39) | 11.21(8.89,13.97) | -1.61(-2.43,-0.78) |
| Mauritania | 510.62(328.57, 712.95) | 55.24(35.55,77.13) | 375.49(250.30, 519.37) | 20.26(13.51,28.03) | -3.19(-3.54,-2.83) |
| Seychelles | 1.81(1.30, 3.26) | 7.63(5.48,13.73) | 2.93(1.73, 3.94) | 12.50(7.39,16.84) | 2.85(2.19,3.51) |
| Marshall Islands | 1.60(1.11, 2.32) | 7.30(5.06,10.57) | 1.01(0.63, 1.64) | 5.77(3.63,9.39) | -0.90(-1.51,-0.29) |
| Burkina Faso | 4,346.35(1,997.89, 6,489.81) | 92.10(42.34,137.52) | 5,546.36(2,908.80, 8,019.03) | 53.47(28.04,77.31) | -1.44(-1.62,-1.26) |
| Monaco | 0.05(0.04, 0.08) | 1.51(1.00,2.22) | 0.08(0.05, 0.11) | 1.51(1.02,2.23) | -0.30(-0.42,-0.18) |
| Fiji | 10.85(7.78, 14.90) | 3.86(2.77,5.29) | 15.77(10.34, 24.57) | 5.79(3.80,9.02) | 2.29(1.64,2.95) |
| Nepal | 8,893.48(5,377.77, 12,073.05) | 105.56(63.83,143.30) | 3,485.11(2,441.34, 4,990.19) | 37.77(26.46,54.08) | -2.94(-3.13,-2.75) |
| Indonesia | 8,467.14(5,400.23, 11,053.10) | 12.50(7.97,16.32) | 4,831.74(3,847.28, 5,980.98) | 7.18(5.72,8.89) | -1.69(-1.79,-1.58) |
| South Sudan | 3,283.27(1,252.63, 5,560.88) | 125.12(47.73,211.91) | 4,293.38(2,631.73, 6,711.68) | 99.96(61.27,156.27) | -0.46(-1.19,0.27) |
| Australia | 297.48(256.70, 351.98) | 7.86(6.78,9.30) | 252.77(196.00, 322.88) | 5.32(4.13,6.80) | -0.44(-0.72,-0.16) |
| Palau | 0.56(0.35, 0.85) | 12.20(7.77,18.75) | 0.20(0.14, 0.28) | 6.11(4.39,8.52) | -1.93(-2.25,-1.61) |
| Senegal | 3,352.14(1,881.08, 4,820.33) | 91.81(51.52,132.02) | 1,640.83(1,166.83, 2,304.60) | 25.80(18.34,36.23) | -3.59(-3.92,-3.27) |
| Greece | 39.84(28.89, 56.44) | 1.97(1.43,2.79) | 30.56(22.80, 41.98) | 2.19(1.63,3.01) | 0.80(0.62,0.98) |
| Niger | 5,321.05(2,244.05, 7,936.48) | 130.97(55.23,195.34) | 4,288.18(2,570.00, 6,269.17) | 33.60(20.13,49.12) | -4.58(-4.89,-4.27) |
| Lithuania | 111.91(94.02, 134.99) | 13.47(11.32,16.25) | 33.13(24.16, 44.74) | 8.12(5.92,10.97) | -1.65(-1.99,-1.31) |
| Kiribati | 10.69(7.03, 15.20) | 36.19(23.80,51.47) | 6.08(3.89, 10.53) | 14.46(9.26,25.07) | -2.89(-3.04,-2.74) |
| Maldives | 31.46(20.96, 40.97) | 29.95(19.96,39.00) | 8.79(6.71, 11.96) | 8.78(6.70,11.94) | -3.26(-3.66,-2.86) |
| Comoros | 187.98(104.93, 294.37) | 88.38(49.34,138.40) | 111.79(76.27, 164.65) | 46.55(31.76,68.56) | -2.23(-2.49,-1.97) |
| Switzerland | 27.86(22.35, 35.77) | 2.41(1.93,3.10) | 28.76(21.97, 37.78) | 2.16(1.65,2.83) | -0.04(-0.19,0.12) |
| United Republic of Tanzania | 14,652.76(7,877.68, 22,765.72) | 121.34(65.24,188.53) | 13,168.21(8,887.77, 19,377.55) | 53.96(36.42,79.41) | -2.05(-2.26,-1.84) |
| Barbados | 10.46(8.93, 12.37) | 16.78(14.32,19.84) | 12.16(9.04, 16.33) | 25.82(19.20,34.67) | 2.09(1.33,2.86) |
| Georgia | 90.21(74.57, 112.13) | 6.59(5.45,8.19) | 31.78(22.59, 42.98) | 4.32(3.07,5.84) | -0.31(-0.99,0.39) |
| Panama | 143.81(120.28, 167.98) | 17.25(14.42,20.14) | 245.35(199.24, 299.25) | 21.27(17.27,25.95) | 1.33(1.05,1.60) |
| Nauru | 0.53(0.36, 0.76) | 12.50(8.65,18.07) | 0.37(0.25, 0.57) | 9.40(6.33,14.38) | -1.06(-1.72,-0.39) |
| Niue | 0.06(0.04, 0.08) | 7.36(5.09,10.41) | 0.10(0.08, 0.13) | 25.19(19.83,32.94) | 1.16(0.16,2.17) |
| Belize | 13.36(11.50, 15.76) | 16.32(14.04,19.25) | 22.69(18.69, 27.77) | 18.43(15.18,22.55) | 1.07(0.35,1.80) |
| Latvia | 115.33(100.35, 133.77) | 20.27(17.64,23.51) | 23.76(17.78, 31.77) | 8.00(5.99,10.69) | -2.47(-2.99,-1.95) |
| Ethiopia | 42,853.74(22,710.06, 63,206.61) | 175.89(93.21,259.43) | 23,304.42(16,129.33, 31,862.84) | 52.55(36.37,71.84) | -4.11(-4.38,-3.83) |
| Angola | 1,896.57(713.48, 2,979.98) | 40.23(15.13,63.20) | 2,089.34(1,349.28, 3,087.65) | 13.70(8.85,20.25) | -3.42(-3.74,-3.09) |
| Kazakhstan | 1,025.40(875.56, 1,208.65) | 19.73(16.85,23.26) | 1,121.40(934.32, 1,358.39) | 20.67(17.22,25.03) | 0.10(-0.30,0.51) |
| Croatia | 126.91(109.02, 148.64) | 12.86(11.05,15.06) | 42.21(32.55, 54.33) | 7.07(5.45,9.10) | -1.16(-1.76,-0.56) |
| Czechia | 319.43(281.47, 365.86) | 14.50(12.77,16.60) | 179.28(130.02, 250.73) | 10.45(7.58,14.61) | -0.91(-1.89,0.07) |
| Papua New Guinea | 306.02(144.78, 505.37) | 18.00(8.52,29.73) | 562.76(311.23, 868.85) | 14.37(7.95,22.18) | -0.57(-0.75,-0.39) |
| Mongolia | 239.71(174.38, 320.37) | 26.63(19.38,35.60) | 108.81(82.34, 142.01) | 10.01(7.58,13.07) | -2.92(-3.42,-2.41) |
| New Zealand | 117.02(104.54, 132.18) | 14.63(13.07,16.52) | 69.62(56.55, 86.00) | 7.09(5.76,8.76) | -0.91(-1.67,-0.15) |
| Trinidad and Tobago | 36.41(29.40, 44.55) | 8.96(7.24,10.96) | 38.59(30.05, 49.92) | 14.17(11.03,18.32) | 2.06(1.22,2.91) |
| Kenya | 5,468.38(3,531.10, 7,402.60) | 48.96(31.61,66.27) | 4,181.36(2,997.48, 6,971.22) | 22.40(16.06,37.35) | -1.47(-1.87,-1.07) |
| Nicaragua | 521.30(402.79, 662.91) | 28.62(22.12,36.40) | 217.96(157.07, 302.70) | 11.01(7.93,15.28) | -2.37(-2.68,-2.06) |
| Iraq | 591.10(416.54, 853.97) | 7.18(5.06,10.37) | 448.42(322.74, 607.40) | 3.33(2.40,4.51) | -2.20(-2.34,-2.06) |
| Ireland | 29.98(24.41, 37.47) | 3.05(2.48,3.81) | 25.17(19.45, 33.45) | 2.52(1.95,3.35) | -0.10(-0.65,0.45) |
| Jordan | 125.38(97.18, 158.26) | 7.68(5.95,9.69) | 196.77(143.61, 273.52) | 5.42(3.95,7.53) | -1.01(-1.13,-0.89) |
| United Kingdom | 291.10(249.31, 350.27) | 2.67(2.28,3.21) | 462.76(406.20, 529.64) | 3.93(3.45,4.49) | 1.84(0.52,3.19) |
| Iceland | 1.03(0.68, 1.53) | 1.62(1.07,2.41) | 1.35(0.95, 1.87) | 2.00(1.41,2.77) | 0.86(0.41,1.31) |
| Cuba | 117.40(82.00, 167.77) | 4.69(3.27,6.70) | 103.63(76.74, 140.50) | 5.83(4.32,7.91) | 1.19(1.00,1.39) |
| Botswana | 22.83(13.88, 32.74) | 3.87(2.35,5.55) | 34.20(20.28, 49.50) | 4.90(2.90,7.09) | 1.37(1.13,1.61) |
| Turkey | 4,418.55(3,017.89, 6,065.40) | 21.57(14.73,29.60) | 1,174.15(884.51, 1,533.05) | 6.34(4.78,8.28) | -3.54(-3.69,-3.38) |
| Hungary | 240.84(207.67, 279.76) | 11.30(9.74,13.13) | 88.88(66.14, 119.33) | 6.40(4.76,8.59) | -1.10(-1.88,-0.31) |
| Bolivia (Plurinational State of) | 2,456.84(1,916.43, 3,151.31) | 91.47(71.35,117.33) | 1,282.73(888.19, 1,773.22) | 36.79(25.47,50.86) | -2.58(-2.69,-2.47) |
| United Arab Emirates | 65.60(49.10, 89.27) | 11.13(8.33,15.15) | 61.71(45.04, 84.18) | 4.61(3.36,6.29) | -1.89(-2.22,-1.56) |
| Argentina | 565.90(493.04, 668.71) | 5.58(4.86,6.60) | 1,090.09(931.32, 1,270.84) | 10.70(9.15,12.48) | 2.73(2.09,3.38) |
| Taiwan (Province of China) | 388.00(356.47, 419.61) | 7.04(6.47,7.62) | 217.30(189.71, 249.75) | 7.37(6.44,8.48) | 1.22(0.35,2.10) |
| Malaysia | 496.50(343.60, 669.67) | 7.55(5.23,10.19) | 345.71(260.52, 449.65) | 4.54(3.42,5.91) | -1.08(-1.46,-0.69) |

**Supplementary Table 7**. Frontier analysis of the relationship between SDI and the incidence rate of UTIs in children in 204 countries and territories in 2021.

| value | SDI | frontier | eff_diff | location_name |
| --- | --- | --- | --- | --- |
| 165.0913036 | 0.569854634 | 164.7754962 | 0.315807485 | Democratic People's Republic of Korea |
| 618.207507 | 0.077688109 | 616.8188302 | 1.388676832 | Somalia |
| 276.6945063 | 0.417797443 | 274.9250103 | 1.76949601 | Papua New Guinea |
| 322.1589946 | 0.429360316 | 274.9118739 | 47.24712072 | Solomon Islands |
| 325.0617976 | 0.444667619 | 274.9173074 | 50.14449019 | Timor-Leste |
| 186.4558348 | 0.72162976 | 135.6211289 | 50.83470583 | China |
| 328.9028141 | 0.473100706 | 260.2645584 | 68.63825574 | Vanuatu |
| 330.3280047 | 0.473621491 | 260.33803 | 69.98997472 | Cambodia |
| 210.5624975 | 0.874747053 | 135.3934986 | 75.16899888 | Taiwan (Province of China) |
| 317.3088686 | 0.489136091 | 219.4092499 | 97.89961875 | Lao People's Democratic Republic |
| 286.0896585 | 0.527186583 | 168.0850116 | 118.0046469 | Kiribati |
| 316.6689847 | 0.576620529 | 164.8080689 | 151.8609158 | Tuvalu |
| 324.8468872 | 0.53390084 | 166.6975678 | 158.1493194 | Myanmar |
| 322.6985769 | 0.625177834 | 161.8789617 | 160.8196151 | Nauru |
| 336.0088595 | 0.574091128 | 164.8509605 | 171.1578991 | Marshall Islands |
| 341.3140575 | 0.587534967 | 164.8064422 | 176.5076153 | Micronesia (Federated States of) |
| 349.7455737 | 0.675051631 | 140.271037 | 209.4745367 | Fiji |
| 351.9021364 | 0.686425621 | 138.3740537 | 213.5280827 | Tokelau |
| 355.9078547 | 0.754046931 | 135.5816245 | 220.3262303 | Palau |
| 387.3060663 | 0.593392769 | 164.8363989 | 222.4696674 | Samoa |
| 362.9427908 | 0.72622205 | 136.052706 | 226.8900849 | Niue |
| 395.2446086 | 0.626349936 | 161.9403804 | 233.3042282 | Tonga |
| 376.0257254 | 0.723727533 | 135.4988341 | 240.5268914 | American Samoa |
| 863.0321661 | 0.168072774 | 616.7898463 | 246.2423198 | Niger |
| 402.4737262 | 0.650886627 | 152.504801 | 249.9689253 | Maldives |
| 890.9757531 | 0.240436019 | 616.6789826 | 274.2967705 | Chad |
| 438.3140669 | 0.651219329 | 151.7231357 | 286.5909312 | Philippines |
| 435.5754827 | 0.803982203 | 136.2367813 | 299.3387014 | Guam |
| 438.7032293 | 0.779109955 | 135.6837563 | 303.019473 | Cook Islands |
| 459.7829242 | 0.656868336 | 148.6351303 | 311.1477939 | Indonesia |
| 447.7146828 | 0.771535213 | 136.5623943 | 311.1522885 | Northern Mariana Islands |
| 455.3703035 | 0.682547933 | 138.2986066 | 317.0716969 | Thailand |
| 481.4267382 | 0.701534935 | 138.4047331 | 343.0220051 | Sri Lanka |
| 647.7157819 | 0.278371125 | 300.7336834 | 346.9820985 | South Sudan |
| 644.594374 | 0.326462614 | 276.2024369 | 368.3919371 | Mozambique |
| 506.4715568 | 0.742523828 | 135.4372015 | 371.0343553 | Malaysia |
| 673.3818835 | 0.289374365 | 300.3310037 | 373.0508798 | Burundi |
| 519.1885913 | 0.730150775 | 136.1730553 | 383.0155361 | Seychelles |
| 521.45523 | 0.718260446 | 135.7056128 | 385.7496172 | Mauritius |
| 671.911295 | 0.423261181 | 274.923048 | 396.988247 | Uganda |
| 682.0136262 | 0.384553634 | 274.9315453 | 407.0820809 | Malawi |
| 685.6389062 | 0.446568273 | 274.9126521 | 410.7262541 | United Republic of Tanzania |
| 686.193707 | 0.403863943 | 274.9259505 | 411.2677566 | Eritrea |
| 577.7477033 | 0.627933721 | 161.7644286 | 415.9832747 | Viet Nam |
| 691.0321682 | 0.400246943 | 274.9258947 | 416.1062734 | Madagascar |
| 692.1973883 | 0.435588706 | 274.9216421 | 417.2757462 | Rwanda |
| 729.8463752 | 0.475978688 | 243.4301711 | 486.4162041 | Comoros |
| 704.3485229 | 0.487958371 | 214.387697 | 489.9608259 | Djibouti |
| 686.7838917 | 0.505948954 | 168.9615133 | 517.8223784 | Zambia |
| 889.5457467 | 0.268579941 | 344.5485633 | 544.9971834 | Mali |
| 862.4903399 | 0.30916769 | 300.3637919 | 562.126548 | Central African Republic |
| 902.9641572 | 0.285118402 | 300.4091327 | 602.5550245 | Burkina Faso |
| 891.3225981 | 0.336401293 | 274.9019307 | 616.4206674 | Guinea |
| 895.9319415 | 0.453721949 | 274.9193355 | 621.0126061 | Angola |
| 902.3042113 | 0.358665881 | 274.9300091 | 627.3742022 | Sierra Leone |
| 912.3204089 | 0.383179849 | 274.9253648 | 637.3950442 | Democratic Republic of the Congo |
| 928.6518133 | 0.353109621 | 274.9216826 | 653.7301307 | Guinea-Bissau |
| 943.426727 | 0.358823295 | 274.9120677 | 668.5146593 | Ethiopia |
| 948.6661607 | 0.352442452 | 274.9185128 | 673.7476479 | Liberia |
| 952.4527734 | 0.373486574 | 274.9272071 | 677.5255663 | Benin |
| 960.2111758 | 0.425941883 | 274.9185262 | 685.2926496 | Côte d'Ivoire |
| 982.1915087 | 0.408533695 | 274.9090136 | 707.2824951 | Togo |
| 959.3560306 | 0.479691223 | 242.9745934 | 716.3814372 | Cameroon |
| 1034.605646 | 0.408054193 | 274.9228286 | 759.6828169 | Senegal |
| 913.0121485 | 0.657857456 | 145.631261 | 767.3808875 | Equatorial Guinea |
| 1070.004415 | 0.40971416 | 274.9168676 | 795.0875471 | Gambia |
| 983.6493005 | 0.583075236 | 164.8225965 | 818.826704 | Congo |
| 1017.4082 | 0.56493039 | 164.8924642 | 852.5157356 | Ghana |
| 1161.386093 | 0.433174635 | 274.9202776 | 886.4658156 | Nepal |
| 1083.245623 | 0.4989451 | 172.0282331 | 911.2173894 | Mauritania |
| 1077.942103 | 0.634691393 | 155.8334585 | 922.1086448 | Gabon |
| 1094.759238 | 0.523768077 | 168.4241835 | 926.3350543 | Kenya |
| 1118.383663 | 0.505413747 | 169.0810211 | 949.3026415 | Sao Tome and Principe |
| 1160.443245 | 0.859000182 | 135.9297704 | 1024.513474 | United Kingdom |
| 1222.32308 | 0.533534539 | 166.9803463 | 1055.342734 | Cabo Verde |
| 1209.048856 | 0.835630545 | 135.6650816 | 1073.383774 | Cyprus |
| 1229.182845 | 0.801585034 | 135.4338742 | 1093.748971 | Malta |
| 1447.196012 | 0.492420885 | 214.8730259 | 1232.322986 | Bangladesh |
| 1511.06002 | 0.473062378 | 260.6271923 | 1250.432828 | Bhutan |
| 1602.864148 | 0.473819486 | 261.9314155 | 1340.932732 | Zimbabwe |
| 1537.669863 | 0.510393066 | 168.5268246 | 1369.143039 | Lesotho |
| 1572.411764 | 0.585459713 | 164.8795083 | 1407.532256 | Eswatini |
| 1632.525879 | 0.933059111 | 135.623256 | 1496.902623 | Switzerland |
| 1671.689294 | 0.617564872 | 164.8213289 | 1506.867965 | Namibia |
| 1662.563016 | 0.744151851 | 135.4442528 | 1527.118763 | Portugal |
| 1689.850468 | 0.642721629 | 151.2427314 | 1538.607736 | Botswana |
| 1754.351384 | 0.503390833 | 168.9070025 | 1585.444382 | Nigeria |
| 1746.54581 | 0.809011652 | 135.9474021 | 1610.598408 | Israel |
| 1779.577435 | 0.679626598 | 138.7414671 | 1640.835968 | South Africa |
| 1793.820066 | 0.884428955 | 136.1491949 | 1657.670872 | Luxembourg |
| 1803.478981 | 0.838364875 | 135.2125316 | 1668.266449 | France |
| 1807.285184 | 0.853654016 | 136.3759898 | 1670.909195 | Belgium |
| 1807.900104 | 0.908262831 | 136.5820972 | 1671.318007 | Monaco |
| 1810.505955 | 0.888005474 | 135.758005 | 1674.74795 | San Marino |
| 1835.933392 | 0.888464256 | 135.4856352 | 1700.447757 | Netherlands |
| 1838.314191 | 0.791854408 | 135.3412126 | 1702.972979 | Greece |
| 1853.067617 | 0.87375385 | 135.6899322 | 1717.377685 | Ireland |
| 1875.576867 | 0.769283698 | 135.9640357 | 1739.612832 | Spain |
| 2076.89822 | 0.337199998 | 274.9299788 | 1801.968241 | Afghanistan |
| 1944.430762 | 0.87636168 | 135.4886968 | 1808.942065 | Iceland |
| 2004.15062 | 0.869444113 | 135.5075536 | 1868.643066 | Andorra |
| 2004.550302 | 0.805773534 | 135.4171447 | 1869.133157 | Italy |
| 2091.845603 | 0.902957091 | 135.6604391 | 1956.185164 | Germany |
| 2110.874252 | 0.896424204 | 135.9756919 | 1974.89856 | Denmark |
| 2323.536425 | 0.450376375 | 274.920652 | 2048.615773 | Yemen |
| 2251.309076 | 0.826210336 | 135.9847054 | 2115.324371 | Greenland |
| 2292.491405 | 0.541949735 | 165.4148684 | 2127.076537 | Sudan |
| 2277.244115 | 0.723122973 | 135.9040216 | 2141.340094 | Argentina |
| 2317.952354 | 0.719283445 | 135.2615653 | 2182.690788 | Uruguay |
| 2341.320258 | 0.859831368 | 135.983484 | 2205.336774 | Finland |
| 2441.938042 | 0.771514716 | 135.9867102 | 2305.951332 | Chile |
| 2559.381647 | 0.606787094 | 164.8139047 | 2394.567742 | Egypt |
| 2560.464569 | 0.853837004 | 135.7310634 | 2424.733505 | Austria |
| 2599.563371 | 0.91613281 | 135.5277109 | 2464.03566 | Norway |
| 2643.769186 | 0.886880299 | 135.5176046 | 2508.251581 | Sweden |
| 2679.839186 | 0.562698301 | 164.8370551 | 2515.002131 | Morocco |
| 2712.561513 | 0.659500924 | 143.1927591 | 2569.368754 | Algeria |
| 2759.378501 | 0.662626231 | 146.1933685 | 2613.185133 | Iraq |
| 2784.880667 | 0.862448354 | 135.7567025 | 2649.123965 | United States of America |
| 2901.58974 | 0.712692673 | 136.8826757 | 2764.707064 | Turkey |
| 2944.720683 | 0.773391602 | 135.4303037 | 2809.290379 | Oman |
| 2988.124147 | 0.697207398 | 138.0350197 | 2850.089127 | Iran (Islamic Republic of) |
| 3021.750693 | 0.631011665 | 156.4394638 | 2865.311229 | Palestine |
| 3009.262139 | 0.682432216 | 139.5011619 | 2869.760977 | Tunisia |
| 3157.513327 | 0.448278285 | 274.933031 | 2882.580296 | Haiti |
| 3026.854146 | 0.815143493 | 135.4848151 | 2891.369331 | Saudi Arabia |
| 3081.994034 | 0.87317068 | 135.3682826 | 2946.625751 | Canada |
| 3109.032386 | 0.623004075 | 161.6845367 | 2947.347849 | Syrian Arab Republic |
| 3083.212517 | 0.725771399 | 135.5914757 | 2947.621041 | Libya |
| 3156.372153 | 0.744746351 | 136.5010123 | 3019.87114 | Lebanon |
| 3194.861648 | 0.617621565 | 164.7628011 | 3030.098847 | Mongolia |
| 3234.003264 | 0.541511187 | 165.48688 | 3068.516384 | Tajikistan |
| 3234.324599 | 0.792416294 | 136.9689355 | 3097.355663 | Serbia |
| 3297.761072 | 0.812042809 | 136.3550298 | 3161.406043 | Poland |
| 3310.351859 | 0.682160776 | 137.6106054 | 3172.741254 | Turkmenistan |
| 3361.416944 | 0.694851274 | 137.2267687 | 3224.190175 | Azerbaijan |
| 3388.526102 | 0.753043204 | 135.8312743 | 3252.694828 | Bahrain |
| 3389.765037 | 0.706849791 | 136.7161631 | 3253.048874 | Albania |
| 3410.976373 | 0.849317734 | 135.838051 | 3275.138322 | United Arab Emirates |
| 3498.279342 | 0.662621694 | 145.0239424 | 3353.2554 | Uzbekistan |
| 3535.752047 | 0.846651055 | 136.2991613 | 3399.452886 | Kuwait |
| 3658.302082 | 0.603979328 | 164.8395645 | 3493.462518 | Kyrgyzstan |
| 3780.550756 | 0.504028689 | 168.8552777 | 3611.695478 | Pakistan |
| 3753.109593 | 0.725144495 | 135.6183702 | 3617.491223 | Kazakhstan |
| 3788.191619 | 0.725307227 | 135.575588 | 3652.616031 | Jordan |
| 3812.828148 | 0.846860584 | 135.8366357 | 3676.991512 | Qatar |
| 3844.870661 | 0.844252814 | 135.6081584 | 3709.262503 | Australia |
| 3856.490782 | 0.750629703 | 135.6651739 | 3720.825608 | North Macedonia |
| 4000.549412 | 0.619388201 | 164.7979349 | 3835.751477 | Dominican Republic |
| 4069.768152 | 0.575401649 | 164.7787795 | 3904.989373 | India |
| 4113.410786 | 0.633665739 | 156.906842 | 3956.503944 | Suriname |
| 4150.300885 | 0.650812335 | 150.8827675 | 3999.418117 | Guyana |
| 4198.881413 | 0.599010799 | 164.8454637 | 4034.03595 | Bolivia (Plurinational State of) |
| 4221.246189 | 0.849442499 | 135.4491335 | 4085.797055 | New Zealand |
| 4415.207894 | 0.732473604 | 135.7328469 | 4279.475047 | Georgia |
| 4491.322547 | 0.610229002 | 164.8434674 | 4326.479079 | Belize |
| 4502.71186 | 0.637195963 | 156.5357095 | 4346.176151 | Saint Vincent and the Grenadines |
| 4530.12219 | 0.635718099 | 156.5766534 | 4373.545536 | Paraguay |
| 4512.564882 | 0.795800584 | 135.927451 | 4376.63743 | Montenegro |
| 4522.671342 | 0.810234367 | 135.8765303 | 4386.794812 | Brunei Darussalam |
| 4564.750949 | 0.662054037 | 146.3842204 | 4418.366729 | Peru |
| 4608.625167 | 0.754987055 | 135.5216502 | 4473.103516 | Saint Kitts and Nevis |
| 4622.99428 | 0.746967185 | 136.0054249 | 4486.988855 | Dominica |
| 4630.935951 | 0.790754768 | 136.0934711 | 4494.84248 | Hungary |
| 4700.029763 | 0.723077893 | 135.7654966 | 4564.264266 | Bosnia and Herzegovina |
| 4726.959277 | 0.768763254 | 136.0076368 | 4590.95164 | Trinidad and Tobago |
| 4756.517424 | 0.668993028 | 140.5257837 | 4615.99164 | Grenada |
| 4814.798359 | 0.886675267 | 136.3112646 | 4678.487095 | Republic of Korea |
| 4827.459933 | 0.81061053 | 135.5034373 | 4691.956496 | Slovakia |
| 4910.473799 | 0.683263064 | 139.4526664 | 4771.021133 | Jamaica |
| 5076.970155 | 0.653043887 | 148.9168397 | 4928.053315 | Brazil |
| 5067.303625 | 0.672509735 | 138.7776425 | 4928.525982 | Saint Lucia |
| 5091.833574 | 0.805020668 | 135.7018753 | 4956.131698 | Bahamas |
| 5285.788005 | 0.821830853 | 135.6059596 | 5150.182045 | United States Virgin Islands |
| 5298.487377 | 0.668729864 | 140.6198274 | 5157.86755 | Cuba |
| 5293.535037 | 0.746748764 | 135.5506118 | 5157.984425 | Barbados |
| 5335.149207 | 0.749886887 | 135.3897406 | 5199.759467 | Antigua and Barbuda |
| 5414.770881 | 0.539972424 | 166.2685869 | 5248.502295 | Guatemala |
| 5507.210768 | 0.798341027 | 135.8125285 | 5371.398239 | Croatia |
| 5631.37661 | 0.513037248 | 168.6396742 | 5462.736936 | Honduras |
| 5720.307976 | 0.821365422 | 135.7236147 | 5584.584361 | Bermuda |
| 5763.859759 | 0.856097766 | 136.145298 | 5627.714461 | Singapore |
| 5776.703109 | 0.661017053 | 143.3835243 | 5633.319585 | Ecuador |
| 5820.995981 | 0.732214875 | 135.5738176 | 5685.422164 | Republic of Moldova |
| 5867.851107 | 0.523958472 | 168.4829078 | 5699.368199 | Nicaragua |
| 5984.067589 | 0.563775188 | 164.8093595 | 5819.25823 | El Salvador |
| 5976.980023 | 0.825525847 | 135.814136 | 5841.165887 | Puerto Rico |
| 6457.672332 | 0.596513059 | 164.8104665 | 6292.861865 | Venezuela (Bolivarian Republic of) |
| 6456.665332 | 0.784484711 | 136.0093237 | 6320.656008 | Belarus |
| 6498.349004 | 0.655442913 | 146.3674014 | 6351.981602 | Colombia |
| 6503.672623 | 0.708864828 | 137.3244479 | 6366.348175 | Panama |
| 6580.237048 | 0.871241813 | 136.0186591 | 6444.218389 | Japan |
| 6638.517484 | 0.830663516 | 135.4035487 | 6503.113935 | Latvia |
| 6900.457017 | 0.844917787 | 135.3738773 | 6765.08314 | Estonia |
| 6923.518681 | 0.856484049 | 135.8293829 | 6787.689298 | Lithuania |
| 6996.36038 | 0.768453864 | 135.6327187 | 6860.727662 | Romania |
| 7490.82482 | 0.700340477 | 136.5838996 | 7354.240921 | Costa Rica |
| 7565.014839 | 0.768150939 | 135.7993303 | 7429.215508 | Bulgaria |
| 8078.725221 | 0.701833194 | 136.903172 | 7941.822049 | Armenia |
| 10489.05528 | 0.664575304 | 144.6760668 | 10344.37921 | Mexico |
| 10480.51733 | 0.808536005 | 135.5193062 | 10344.99803 | Russian Federation |
| 10489.09929 | 0.842430731 | 136.2560124 | 10352.84328 | Slovenia |
| 10653.77606 | 0.828450433 | 135.6881514 | 10518.08791 | Czechia |
| 12182.6968 | 0.760773913 | 136.3967108 | 12046.30009 | Ukraine |

**Supplementary Table 8** . Frontier analysis of the relationship between SDI and the mortality rate of UTIs in children in 204 countries and territories in 2021.

| **value** | **SDI** | **frontier** | **eff_diff** | **location_name** |
| --- | --- | --- | --- | --- |
| 0.377679656 | 0.168072774 | 0.377679656 | 0 | Niger |
| 0.462704439 | 0.077688109 | 0.462704439 | 0 | Somalia |
| 0.001745901 | 0.795800584 | 0.001180558 | 0.000565343 | Montenegro |
| 0.002188233 | 0.750629703 | 0.00154624 | 0.000641993 | North Macedonia |
| 0.002387722 | 0.779109955 | 0.001336085 | 0.001051637 | Cook Islands |
| 0.002464307 | 0.846860584 | 0.001344288 | 0.001120019 | Qatar |
| 0.003459812 | 0.888005474 | 0.001180748 | 0.002279064 | San Marino |
| 0.004787784 | 0.908262831 | 0.001259888 | 0.003527896 | Monaco |
| 0.005309114 | 0.884428955 | 0.001202066 | 0.004107047 | Luxembourg |
| 0.005692863 | 0.91613281 | 0.001200739 | 0.004492124 | Norway |
| 0.006382789 | 0.792416294 | 0.001248342 | 0.005134447 | Serbia |
| 0.013043312 | 0.627933721 | 0.006525719 | 0.006517593 | Viet Nam |
| 0.009434881 | 0.886675267 | 0.001305444 | 0.008129438 | Republic of Korea |
| 0.009774583 | 0.87636168 | 0.001186462 | 0.00858812 | Iceland |
| 0.010157438 | 0.886880299 | 0.001176431 | 0.008981006 | Sweden |
| 0.010468559 | 0.853837004 | 0.001243163 | 0.009225396 | Austria |
| 0.012441001 | 0.706849791 | 0.002725457 | 0.009715544 | Albania |
| 0.011337859 | 0.753043204 | 0.001612665 | 0.009725194 | Bahrain |
| 0.016863567 | 0.606787094 | 0.006960397 | 0.00990317 | Egypt |
| 0.012262798 | 0.723077893 | 0.002344446 | 0.009918352 | Bosnia and Herzegovina |
| 0.012776889 | 0.791854408 | 0.001267088 | 0.011509801 | Greece |
| 0.013337815 | 0.805773534 | 0.001233751 | 0.012104064 | Italy |
| 0.01335409 | 0.869444113 | 0.00118322 | 0.01217087 | Andorra |
| 0.013652302 | 0.933059111 | 0.001272332 | 0.01237997 | Switzerland |
| 0.015619859 | 0.72162976 | 0.002447858 | 0.013172001 | China |
| 0.014526814 | 0.842430731 | 0.001186816 | 0.013339998 | Slovenia |
| 0.01461486 | 0.902957091 | 0.001189992 | 0.013424868 | Germany |
| 0.018401026 | 0.662626231 | 0.004520844 | 0.013880182 | Iraq |
| 0.016359205 | 0.87375385 | 0.001264244 | 0.01509496 | Ireland |
| 0.017417914 | 0.732473604 | 0.002304789 | 0.015113124 | Georgia |
| 0.01719195 | 0.87317068 | 0.001309476 | 0.015882474 | Canada |
| 0.017326668 | 0.838364875 | 0.001254728 | 0.01607194 | France |
| 0.0246368 | 0.631011665 | 0.006479962 | 0.018156838 | Palestine |
| 0.019401186 | 0.859831368 | 0.001166081 | 0.018235105 | Finland |
| 0.020275197 | 0.896424204 | 0.001188879 | 0.019086318 | Denmark |
| 0.02042552 | 0.853654016 | 0.001160325 | 0.019265195 | Belgium |
| 0.027667291 | 0.617564872 | 0.00665773 | 0.021009562 | Namibia |
| 0.023068412 | 0.871241813 | 0.001259108 | 0.021809304 | Japan |
| 0.023504694 | 0.835630545 | 0.00117497 | 0.022329724 | Cyprus |
| 0.028556014 | 0.668729864 | 0.004501578 | 0.024054436 | Cuba |
| 0.034583804 | 0.569854634 | 0.00970486 | 0.024878945 | Democratic People's Republic of Korea |
| 0.030054183 | 0.679626598 | 0.00388145 | 0.026172733 | South Africa |
| 0.027905163 | 0.81061053 | 0.001280665 | 0.026624498 | Slovakia |
| 0.028375113 | 0.849317734 | 0.001191538 | 0.027183575 | United Arab Emirates |
| 0.029505685 | 0.769283698 | 0.001376007 | 0.028129678 | Spain |
| 0.029387506 | 0.821365422 | 0.001164293 | 0.028223213 | Bermuda |
| 0.030387022 | 0.744151851 | 0.001916197 | 0.028470825 | Portugal |
| 0.038409229 | 0.619388201 | 0.006576963 | 0.031832266 | Dominican Republic |
| 0.033250438 | 0.844252814 | 0.001229114 | 0.032021324 | Australia |
| 0.033430766 | 0.809011652 | 0.001197618 | 0.032233148 | Israel |
| 0.035048171 | 0.725307227 | 0.002381099 | 0.032667072 | Jordan |
| 0.037841227 | 0.859000182 | 0.001218962 | 0.036622264 | United Kingdom |
| 0.051705223 | 0.510393066 | 0.015065655 | 0.036639568 | Lesotho |
| 0.038071665 | 0.862448354 | 0.00131121 | 0.036760455 | United States of America |
| 0.054135394 | 0.473819486 | 0.016865433 | 0.037269961 | Zimbabwe |
| 0.038530722 | 0.790754768 | 0.001250437 | 0.037280285 | Hungary |
| 0.039648428 | 0.801585034 | 0.001132492 | 0.038515937 | Malta |
| 0.045078843 | 0.642721629 | 0.006191713 | 0.03888713 | Botswana |
| 0.040350402 | 0.798341027 | 0.00123673 | 0.039113672 | Croatia |
| 0.040467886 | 0.768453864 | 0.001352514 | 0.039115372 | Romania |
| 0.040399453 | 0.828450433 | 0.001261053 | 0.0391384 | Czechia |
| 0.040596779 | 0.844917787 | 0.001275884 | 0.039320895 | Estonia |
| 0.040666346 | 0.826210336 | 0.001226306 | 0.03944004 | Greenland |
| 0.041827052 | 0.812042809 | 0.00118618 | 0.040640872 | Poland |
| 0.042304005 | 0.856484049 | 0.001239155 | 0.04106485 | Lithuania |
| 0.043277741 | 0.888464256 | 0.001288541 | 0.0419892 | Netherlands |
| 0.043476573 | 0.830663516 | 0.001201136 | 0.042275436 | Latvia |
| 0.045255 | 0.821830853 | 0.00118754 | 0.04406746 | United States Virgin Islands |
| 0.06188418 | 0.473100706 | 0.016944164 | 0.044940017 | Vanuatu |
| 0.054556365 | 0.585459713 | 0.007176605 | 0.04737976 | Eswatini |
| 0.055274829 | 0.587534967 | 0.007324123 | 0.047950706 | Micronesia (Federated States of) |
| 0.050772452 | 0.742523828 | 0.001923203 | 0.048849249 | Malaysia |
| 0.050208562 | 0.849442499 | 0.001218161 | 0.048990401 | New Zealand |
| 0.052272938 | 0.697207398 | 0.003001365 | 0.049271573 | Iran (Islamic Republic of) |
| 0.064695094 | 0.533534539 | 0.015029985 | 0.049665109 | Cabo Verde |
| 0.052203529 | 0.712692673 | 0.002510614 | 0.049692916 | Turkey |
| 0.055592236 | 0.682432216 | 0.00358033 | 0.052011906 | Tunisia |
| 0.053326193 | 0.771535213 | 0.00124113 | 0.052085063 | Northern Mariana Islands |
| 0.060594354 | 0.593392769 | 0.007414855 | 0.053179499 | Samoa |
| 0.072082859 | 0.429360316 | 0.018821742 | 0.053261117 | Solomon Islands |
| 0.054875798 | 0.760773913 | 0.00152814 | 0.053347659 | Ukraine |
| 0.062117598 | 0.574091128 | 0.00870008 | 0.053417518 | Marshall Islands |
| 0.055009372 | 0.784484711 | 0.001330204 | 0.053679169 | Belarus |
| 0.062133126 | 0.675051631 | 0.004130994 | 0.058002132 | Fiji |
| 0.067465226 | 0.576620529 | 0.009050508 | 0.058414718 | Tuvalu |
| 0.062116649 | 0.815143493 | 0.001202432 | 0.060914217 | Saudi Arabia |
| 0.063727597 | 0.808536005 | 0.001190877 | 0.06253672 | Russian Federation |
| 0.06613755 | 0.718260446 | 0.002567733 | 0.063569817 | Mauritius |
| 0.065648393 | 0.754046931 | 0.001629826 | 0.064018567 | Palau |
| 0.068434418 | 0.825525847 | 0.00118983 | 0.067244588 | Puerto Rico |
| 0.08298314 | 0.523958472 | 0.015024396 | 0.067958744 | Nicaragua |
| 0.073953926 | 0.768150939 | 0.001378554 | 0.072575372 | Bulgaria |
| 0.080196513 | 0.626349936 | 0.006526609 | 0.073669904 | Tonga |
| 0.079630967 | 0.701534935 | 0.003004335 | 0.076626632 | Sri Lanka |
| 0.10505427 | 0.383179849 | 0.028073176 | 0.076981095 | Democratic Republic of the Congo |
| 0.082974952 | 0.656868336 | 0.005025556 | 0.077949396 | Indonesia |
| 0.100366098 | 0.562698301 | 0.014990643 | 0.085375455 | Morocco |
| 0.087590571 | 0.874747053 | 0.001211659 | 0.086378911 | Taiwan (Province of China) |
| 0.088724989 | 0.771514716 | 0.001264043 | 0.087460946 | Chile |
| 0.096041797 | 0.617621565 | 0.006736416 | 0.089305381 | Mongolia |
| 0.091062962 | 0.746967185 | 0.001648267 | 0.089414695 | Dominica |
| 0.09136354 | 0.744746351 | 0.001939646 | 0.089423894 | Lebanon |
| 0.094574518 | 0.659500924 | 0.004943551 | 0.089630967 | Algeria |
| 0.091297571 | 0.846651055 | 0.001271833 | 0.090025738 | Kuwait |
| 0.093449738 | 0.719283445 | 0.002585298 | 0.09086444 | Uruguay |
| 0.0926771 | 0.856097766 | 0.001189471 | 0.091487629 | Singapore |
| 0.09900817 | 0.668993028 | 0.004250741 | 0.094757429 | Grenada |
| 0.096185585 | 0.810234367 | 0.001186769 | 0.094998816 | Brunei Darussalam |
| 0.100917032 | 0.650886627 | 0.005713604 | 0.095203428 | Maldives |
| 0.098396733 | 0.723727533 | 0.002380116 | 0.096016617 | American Samoa |
| 0.102852783 | 0.625177834 | 0.006524138 | 0.096328645 | Nauru |
| 0.104656844 | 0.583075236 | 0.008194082 | 0.096462762 | Congo |
| 0.099195427 | 0.732214875 | 0.002404604 | 0.096790823 | Republic of Moldova |
| 0.100621874 | 0.773391602 | 0.00127434 | 0.099347534 | Oman |
| 0.105979648 | 0.700340477 | 0.003038311 | 0.102941338 | Costa Rica |
| 0.107492399 | 0.803982203 | 0.001211187 | 0.106281212 | Guam |
| 0.124385821 | 0.450376375 | 0.018040775 | 0.106345046 | Yemen |
| 0.109271718 | 0.723122973 | 0.002316173 | 0.106955545 | Argentina |
| 0.120424724 | 0.634691393 | 0.006486652 | 0.113938072 | Gabon |
| 0.1196945 | 0.725771399 | 0.002451094 | 0.117243406 | Libya |
| 0.26392829 | 0.30916769 | 0.146228656 | 0.117699634 | Central African Republic |
| 0.124451551 | 0.635718099 | 0.006487506 | 0.117964045 | Paraguay |
| 0.12310246 | 0.672509735 | 0.004125979 | 0.118976481 | Saint Lucia |
| 0.122585116 | 0.683263064 | 0.003547468 | 0.119037648 | Jamaica |
| 0.129236533 | 0.701833194 | 0.003014679 | 0.126221853 | Armenia |
| 0.129287217 | 0.749886887 | 0.001740353 | 0.127546864 | Antigua and Barbuda |
| 0.129336131 | 0.768763254 | 0.001333803 | 0.128002328 | Trinidad and Tobago |
| 0.133417729 | 0.657857456 | 0.005015747 | 0.128401982 | Equatorial Guinea |
| 0.25953733 | 0.337199998 | 0.130737568 | 0.128799762 | Afghanistan |
| 0.151004108 | 0.453721949 | 0.017614616 | 0.133389493 | Angola |
| 0.15844911 | 0.417797443 | 0.020325106 | 0.138124004 | Papua New Guinea |
| 0.34303483 | 0.289374365 | 0.204691229 | 0.138343601 | Burundi |
| 0.141008957 | 0.730150775 | 0.002292251 | 0.138716706 | Seychelles |
| 0.149883693 | 0.603979328 | 0.007142208 | 0.142741485 | Kyrgyzstan |
| 0.159551311 | 0.527186583 | 0.015014861 | 0.144536451 | Kiribati |
| 0.171692211 | 0.473621491 | 0.016840703 | 0.154851508 | Cambodia |
| 0.175901125 | 0.444667619 | 0.018061622 | 0.157839504 | Timor-Leste |
| 0.159265277 | 0.805020668 | 0.001189788 | 0.158075488 | Bahamas |
| 0.173667612 | 0.541949735 | 0.015038712 | 0.1586289 | Sudan |
| 0.167620732 | 0.596513059 | 0.006965535 | 0.160655197 | Venezuela (Bolivarian Republic of) |
| 0.169101819 | 0.661017053 | 0.004958775 | 0.164143044 | Ecuador |
| 0.178733999 | 0.610229002 | 0.006871256 | 0.171862743 | Belize |
| 0.197552063 | 0.563775188 | 0.014392164 | 0.183159899 | El Salvador |
| 0.193471236 | 0.662621694 | 0.004679065 | 0.188792171 | Uzbekistan |
| 0.195862429 | 0.682547933 | 0.003512227 | 0.192350201 | Thailand |
| 0.196291468 | 0.708864828 | 0.002801363 | 0.193490105 | Panama |
| 0.219119346 | 0.489136091 | 0.016049053 | 0.203070294 | Lao People's Democratic Republic |
| 0.224050404 | 0.513037248 | 0.014981577 | 0.209068827 | Honduras |
| 0.224521882 | 0.4989451 | 0.015190493 | 0.209331389 | Mauritania |
| 0.215455229 | 0.650812335 | 0.005678348 | 0.209776882 | Guyana |
| 0.213783344 | 0.686425621 | 0.003550847 | 0.210232497 | Tokelau |
| 0.219334866 | 0.725144495 | 0.002244696 | 0.217090171 | Kazakhstan |
| 0.236237298 | 0.505413747 | 0.015175089 | 0.221062209 | Sao Tome and Principe |
| 0.229278018 | 0.637195963 | 0.006468833 | 0.222809185 | Saint Vincent and the Grenadines |
| 0.254698305 | 0.384553634 | 0.027834127 | 0.226864178 | Malawi |
| 0.236007887 | 0.694851274 | 0.003261599 | 0.232746288 | Azerbaijan |
| 0.237742958 | 0.655442913 | 0.004992019 | 0.232750939 | Colombia |
| 0.239467066 | 0.754987055 | 0.001610358 | 0.237856707 | Saint Kitts and Nevis |
| 0.255479403 | 0.523768077 | 0.015016619 | 0.240462784 | Kenya |
| 0.269098439 | 0.40971416 | 0.022960166 | 0.246138274 | Gambia |
| 0.253443043 | 0.664575304 | 0.004472114 | 0.248970929 | Mexico |
| 0.273395822 | 0.408533695 | 0.023526134 | 0.249869688 | Togo |
| 0.262039456 | 0.623004075 | 0.006531096 | 0.25550836 | Syrian Arab Republic |
| 0.271191619 | 0.53390084 | 0.015036897 | 0.256154722 | Myanmar |
| 0.259957098 | 0.746748764 | 0.001774713 | 0.258182384 | Barbados |
| 0.291959787 | 0.400246943 | 0.027643809 | 0.264315979 | Madagascar |
| 0.287468404 | 0.408054193 | 0.022878579 | 0.264589825 | Senegal |
| 0.482546599 | 0.268579941 | 0.214954134 | 0.267592465 | Mali |
| 0.279894174 | 0.72622205 | 0.002439138 | 0.277455036 | Niue |
| 0.294452624 | 0.539972424 | 0.015035862 | 0.279416763 | Guatemala |
| 0.320811851 | 0.353109621 | 0.037034402 | 0.283777448 | Guinea-Bissau |
| 0.292771866 | 0.662054037 | 0.004557277 | 0.288214589 | Peru |
| 0.531385369 | 0.240436019 | 0.230910301 | 0.300475068 | Chad |
| 0.315120151 | 0.651219329 | 0.005772549 | 0.309347603 | Philippines |
| 0.36000622 | 0.352442452 | 0.036537342 | 0.323468877 | Liberia |
| 0.351368463 | 0.487958371 | 0.01606135 | 0.335307113 | Djibouti |
| 0.356413311 | 0.56493039 | 0.011285089 | 0.345128222 | Ghana |
| 0.359687355 | 0.633665739 | 0.006486297 | 0.353201058 | Suriname |
| 0.487471604 | 0.336401293 | 0.130246776 | 0.357224829 | Guinea |
| 0.377235104 | 0.505948954 | 0.015102057 | 0.362133047 | Zambia |
| 0.397075549 | 0.403863943 | 0.027691148 | 0.369384401 | Eritrea |
| 0.396057911 | 0.425941883 | 0.01944961 | 0.376608302 | Côte d'Ivoire |
| 0.388609485 | 0.653043887 | 0.005483519 | 0.383125965 | Brazil |
| 0.392164925 | 0.599010799 | 0.007011406 | 0.385153519 | Bolivia (Plurinational State of) |
| 0.601572583 | 0.285118402 | 0.205110872 | 0.396461711 | Burkina Faso |
| 0.433586915 | 0.433174635 | 0.018916928 | 0.414669987 | Nepal |
| 0.562132352 | 0.326462614 | 0.134792339 | 0.427340013 | Mozambique |
| 0.45580749 | 0.435588706 | 0.018504308 | 0.437303182 | Rwanda |
| 0.455684397 | 0.541511187 | 0.015050964 | 0.440633433 | Tajikistan |
| 0.468306792 | 0.492420885 | 0.015343044 | 0.452963748 | Bangladesh |
| 0.478877929 | 0.575401649 | 0.008835333 | 0.470042596 | India |
| 0.492215441 | 0.448278285 | 0.018086261 | 0.474129181 | Haiti |
| 0.501147584 | 0.479691223 | 0.016698796 | 0.484448788 | Cameroon |
| 0.500056148 | 0.503390833 | 0.015160194 | 0.484895954 | Nigeria |
| 0.545113663 | 0.358665881 | 0.034329725 | 0.510783939 | Sierra Leone |
| 0.539790982 | 0.475978688 | 0.016857165 | 0.522933817 | Comoros |
| 0.569609555 | 0.373486574 | 0.030589587 | 0.539019968 | Benin |
| 0.607754522 | 0.358823295 | 0.031924884 | 0.575829638 | Ethiopia |
| 0.622764976 | 0.446568273 | 0.018010161 | 0.604754815 | United Republic of Tanzania |
| 0.631828429 | 0.473062378 | 0.016924429 | 0.614904 | Bhutan |
| 0.704045856 | 0.682160776 | 0.003551954 | 0.700493902 | Turkmenistan |
| 1.15400213 | 0.278371125 | 0.211525969 | 0.942476161 | South Sudan |
| 1.226304659 | 0.504028689 | 0.015125033 | 1.211179625 | Pakistan |
| 0.467853128 | 0.423261181 | 0.019788731 | 0.448064397064225 | Uganda |

**Supplementary Table 9**. Frontier analysis of the relationship between SDI and the DALYs rate of UTIs in children in 204 countries and territories in 2021.

| **value** | **SDI** | **frontier** | **eff_diff** | **location_name** |
| --- | --- | --- | --- | --- |
| 40.28328164 | 0.077688109 | 40.28328164 | 0 | Somalia |
| 33.5958971 | 0.168072774 | 33.5958971 | 0 | Niger |
| 0.505461875 | 0.779109955 | 0.391045027 | 0.114416848 | Cook Islands |
| 1.418373562 | 0.627933721 | 0.885813139 | 0.532560423 | Viet Nam |
| 1.420076832 | 0.72162976 | 0.517283706 | 0.902793126 | China |
| 1.367929106 | 0.888005474 | 0.400129315 | 0.967799791 | San Marino |
| 1.507504834 | 0.908262831 | 0.406510227 | 1.100994607 | Monaco |
| 1.547139289 | 0.884428955 | 0.410579298 | 1.136559991 | Luxembourg |
| 2.002236319 | 0.87636168 | 0.411804943 | 1.590431376 | Iceland |
| 2.079966145 | 0.91613281 | 0.407548827 | 1.672417318 | Norway |
| 2.975140214 | 0.569854634 | 1.282705389 | 1.692434825 | Democratic People's Republic of Korea |
| 2.158017254 | 0.933059111 | 0.394787289 | 1.763229965 | Switzerland |
| 2.191009049 | 0.791854408 | 0.411574872 | 1.779434176 | Greece |
| 2.323405956 | 0.869444113 | 0.406752895 | 1.916653061 | Andorra |
| 2.431188872 | 0.805773534 | 0.409566522 | 2.02162235 | Italy |
| 2.454826518 | 0.853837004 | 0.402553038 | 2.05227348 | Austria |
| 2.523800101 | 0.87375385 | 0.405968993 | 2.117831107 | Ireland |
| 2.527684713 | 0.902957091 | 0.408589125 | 2.119095588 | Germany |
| 2.516650448 | 0.886880299 | 0.39573753 | 2.120912918 | Sweden |
| 3.066991312 | 0.606787094 | 0.922285533 | 2.144705779 | Egypt |
| 2.577822953 | 0.838364875 | 0.408864573 | 2.16895838 | France |
| 2.670134436 | 0.846860584 | 0.409037805 | 2.26109663 | Qatar |
| 2.741167864 | 0.750629703 | 0.442192309 | 2.298975555 | North Macedonia |
| 2.702999119 | 0.792416294 | 0.403020505 | 2.299978614 | Serbia |
| 2.73160327 | 0.835630545 | 0.413988083 | 2.317615187 | Cyprus |
| 2.836324058 | 0.853654016 | 0.411109779 | 2.42521428 | Belgium |
| 3.400404634 | 0.617564872 | 0.91085756 | 2.489547074 | Namibia |
| 3.331059652 | 0.662626231 | 0.753925734 | 2.577133918 | Iraq |
| 3.016206029 | 0.896424204 | 0.4055138 | 2.610692229 | Denmark |
| 3.066142703 | 0.859831368 | 0.400920471 | 2.665222232 | Finland |
| 3.144839374 | 0.753043204 | 0.423445478 | 2.721393896 | Bahrain |
| 3.173534687 | 0.795800584 | 0.40465896 | 2.768875727 | Montenegro |
| 3.334853297 | 0.706849791 | 0.551779694 | 2.783073603 | Albania |
| 3.63884915 | 0.679626598 | 0.66277144 | 2.976077711 | South Africa |
| 3.447069934 | 0.87317068 | 0.401608448 | 3.045461486 | Canada |
| 4.010948964 | 0.631011665 | 0.90444598 | 3.106502984 | Palestine |
| 3.601458463 | 0.744151851 | 0.45726897 | 3.144189493 | Portugal |
| 3.625974751 | 0.769283698 | 0.408942646 | 3.217032105 | Spain |
| 3.927365819 | 0.859000182 | 0.410569609 | 3.51679621 | United Kingdom |
| 3.942363931 | 0.809011652 | 0.408491385 | 3.533872546 | Israel |
| 3.987038658 | 0.886675267 | 0.402132962 | 3.584905696 | Republic of Korea |
| 5.287418368 | 0.510393066 | 1.629075892 | 3.658342476 | Lesotho |
| 4.194066203 | 0.723077893 | 0.523970601 | 3.670095602 | Bosnia and Herzegovina |
| 4.090907992 | 0.801585034 | 0.40180277 | 3.689105222 | Malta |
| 4.31865634 | 0.732473604 | 0.483917872 | 3.834738468 | Georgia |
| 5.600789209 | 0.473819486 | 1.705134527 | 3.895654683 | Zimbabwe |
| 4.897789851 | 0.642721629 | 0.874899687 | 4.022890164 | Botswana |
| 5.755501365 | 0.473100706 | 1.70181936 | 4.053682005 | Vanuatu |
| 4.540640858 | 0.742523828 | 0.450992718 | 4.08964814 | Malaysia |
| 5.15940245 | 0.587534967 | 1.006702468 | 4.152699982 | Micronesia (Federated States of) |
| 4.609522208 | 0.849317734 | 0.398521317 | 4.211000891 | United Arab Emirates |
| 4.797161971 | 0.888464256 | 0.399891945 | 4.397270026 | Netherlands |
| 4.877779588 | 0.826210336 | 0.406631449 | 4.47114814 | Greenland |
| 5.573696357 | 0.585459713 | 1.051083284 | 4.522613073 | Eswatini |
| 5.771738138 | 0.574091128 | 1.225465485 | 4.546272653 | Marshall Islands |
| 5.041383827 | 0.862448354 | 0.398238515 | 4.643145312 | United States of America |
| 5.065802911 | 0.771535213 | 0.40016833 | 4.665634581 | Northern Mariana Islands |
| 5.682642771 | 0.593392769 | 0.98010548 | 4.702537291 | Samoa |
| 6.395088613 | 0.533534539 | 1.628587957 | 4.766500656 | Cabo Verde |
| 6.660146128 | 0.429360316 | 1.859726406 | 4.800419722 | Solomon Islands |
| 5.416169323 | 0.725307227 | 0.523699038 | 4.892470284 | Jordan |
| 5.322133145 | 0.844252814 | 0.402118812 | 4.920014333 | Australia |
| 5.911899549 | 0.619388201 | 0.906322067 | 5.005577482 | Dominican Republic |
| 6.241441585 | 0.576620529 | 1.177628232 | 5.063813354 | Tuvalu |
| 5.831784913 | 0.668729864 | 0.749488319 | 5.082296593 | Cuba |
| 5.785359731 | 0.675051631 | 0.658751625 | 5.126608106 | Fiji |
| 5.630228206 | 0.81061053 | 0.406833922 | 5.223394285 | Slovakia |
| 5.737763454 | 0.812042809 | 0.400652294 | 5.33711116 | Poland |
| 5.885409757 | 0.718260446 | 0.526258481 | 5.359151276 | Mauritius |
| 6.107791527 | 0.754046931 | 0.429137716 | 5.678653812 | Palau |
| 6.308562141 | 0.697207398 | 0.571520426 | 5.737041714 | Iran (Islamic Republic of) |
| 6.206916544 | 0.821365422 | 0.40774042 | 5.799176125 | Bermuda |
| 6.339752857 | 0.712692673 | 0.516402178 | 5.823350679 | Turkey |
| 6.349456523 | 0.871241813 | 0.397771772 | 5.95168475 | Japan |
| 6.401237588 | 0.790754768 | 0.405898707 | 5.995338881 | Hungary |
| 6.695268844 | 0.682432216 | 0.647416139 | 6.047852705 | Tunisia |
| 6.906553806 | 0.701534935 | 0.56898885 | 6.337564956 | Sri Lanka |
| 9.670242509 | 0.383179849 | 3.322741156 | 6.347501353 | Democratic Republic of the Congo |
| 7.18064204 | 0.656868336 | 0.798066423 | 6.382575617 | Indonesia |
| 7.443780651 | 0.626349936 | 0.888759754 | 6.555020897 | Tonga |
| 7.068826795 | 0.798341027 | 0.40510803 | 6.663718765 | Croatia |
| 7.09193199 | 0.849442499 | 0.405154738 | 6.686777252 | New Zealand |
| 7.20570716 | 0.815143493 | 0.408181542 | 6.797525619 | Saudi Arabia |
| 7.340142546 | 0.821830853 | 0.404344318 | 6.935798227 | United States Virgin Islands |
| 7.37436287 | 0.874747053 | 0.410897025 | 6.963465846 | Taiwan (Province of China) |
| 7.940131276 | 0.844917787 | 0.402099805 | 7.538031471 | Estonia |
| 7.999778816 | 0.830663516 | 0.413052161 | 7.586726654 | Latvia |
| 8.073195832 | 0.768453864 | 0.413007389 | 7.660188443 | Romania |
| 8.123398331 | 0.856484049 | 0.403702891 | 7.71969544 | Lithuania |
| 8.166440275 | 0.842430731 | 0.412515332 | 7.753924943 | Slovenia |
| 8.778941983 | 0.650886627 | 0.827377307 | 7.951564676 | Maldives |
| 8.821280229 | 0.784484711 | 0.400928215 | 8.420352014 | Belarus |
| 9.023899804 | 0.723727533 | 0.529628 | 8.494271804 | American Samoa |
| 9.399198253 | 0.625177834 | 0.902274469 | 8.496923784 | Nauru |
| 9.641200878 | 0.583075236 | 1.107355905 | 8.533844973 | Congo |
| 9.021373829 | 0.771514716 | 0.414237076 | 8.607136753 | Chile |
| 10.42475154 | 0.562698301 | 1.623170626 | 8.801580911 | Morocco |
| 9.382027697 | 0.719283445 | 0.515801277 | 8.86622642 | Uruguay |
| 10.01377158 | 0.617621565 | 0.898433599 | 9.115337985 | Mongolia |
| 9.909286954 | 0.659500924 | 0.766774141 | 9.142512813 | Algeria |
| 9.719004203 | 0.825525847 | 0.40193807 | 9.317066133 | Puerto Rico |
| 11.00600028 | 0.523958472 | 1.62911626 | 9.376884023 | Nicaragua |
| 9.89169699 | 0.744746351 | 0.454032198 | 9.437664792 | Lebanon |
| 9.928650173 | 0.803982203 | 0.401671542 | 9.526978632 | Guam |
| 10.11033499 | 0.846651055 | 0.404981246 | 9.70535374 | Kuwait |
| 10.44563021 | 0.828450433 | 0.403725734 | 10.04190447 | Czechia |
| 11.00833743 | 0.634691393 | 0.889190341 | 10.11914709 | Gabon |
| 10.57492659 | 0.773391602 | 0.414465325 | 10.16046127 | Oman |
| 10.70460448 | 0.723122973 | 0.513233496 | 10.19137098 | Argentina |
| 10.8441581 | 0.746967185 | 0.444279811 | 10.39987829 | Dominica |
| 23.54946781 | 0.30916769 | 13.11629369 | 10.43317412 | Central African Republic |
| 12.3187495 | 0.450376375 | 1.798340856 | 10.52040864 | Yemen |
| 11.20606224 | 0.768150939 | 0.413503253 | 10.79255899 | Bulgaria |
| 11.55796978 | 0.668993028 | 0.694371682 | 10.8635981 | Grenada |
| 11.30509022 | 0.810234367 | 0.411414421 | 10.8936758 | Brunei Darussalam |
| 29.69104058 | 0.289374365 | 18.51599741 | 11.17504317 | Burundi |
| 11.69293233 | 0.856097766 | 0.404433236 | 11.28849909 | Singapore |
| 12.11219208 | 0.657857456 | 0.778782648 | 11.33340944 | Equatorial Guinea |
| 12.12974459 | 0.732214875 | 0.505893138 | 11.62385145 | Republic of Moldova |
| 12.22679775 | 0.725771399 | 0.51526553 | 11.71153222 | Libya |
| 12.2544548 | 0.808536005 | 0.405491883 | 11.84896291 | Russian Federation |
| 13.70464727 | 0.453721949 | 1.780929254 | 11.92371802 | Angola |
| 12.5022769 | 0.730150775 | 0.508454319 | 11.99382259 | Seychelles |
| 24.04181367 | 0.337199998 | 11.98969739 | 12.05211628 | Afghanistan |
| 14.36686298 | 0.417797443 | 2.185239327 | 12.18162365 | Papua New Guinea |
| 12.62235282 | 0.760773913 | 0.413681286 | 12.20867153 | Ukraine |
| 14.46101963 | 0.527186583 | 1.628769228 | 12.8322504 | Kiribati |
| 13.77974273 | 0.635718099 | 0.89336069 | 12.88638204 | Paraguay |
| 14.7051431 | 0.473621491 | 1.701829671 | 13.00331342 | Cambodia |
| 13.68821063 | 0.683263064 | 0.633886726 | 13.0543239 | Jamaica |
| 13.81463605 | 0.672509735 | 0.699826863 | 13.11480918 | Saint Lucia |
| 13.89407619 | 0.700340477 | 0.558913138 | 13.33516305 | Costa Rica |
| 15.16812187 | 0.444667619 | 1.785301335 | 13.38282053 | Timor-Leste |
| 14.16537359 | 0.768763254 | 0.404492125 | 13.76088147 | Trinidad and Tobago |
| 14.81990764 | 0.603979328 | 0.946759646 | 13.873148 | Kyrgyzstan |
| 14.58030149 | 0.749886887 | 0.432967616 | 14.14733387 | Antigua and Barbuda |
| 16.53212859 | 0.541949735 | 1.628979714 | 14.90314888 | Sudan |
| 15.94712301 | 0.701833194 | 0.600887982 | 15.34623502 | Armenia |
| 16.54072834 | 0.682547933 | 0.631887957 | 15.90884039 | Thailand |
| 16.99249916 | 0.805020668 | 0.402008804 | 16.59049036 | Bahamas |
| 18.78027328 | 0.489136091 | 1.677614487 | 17.1026588 | Lao People's Democratic Republic |
| 18.2343224 | 0.661017053 | 0.791798805 | 17.4425236 | Ecuador |
| 18.43096573 | 0.610229002 | 0.897112174 | 17.53385356 | Belize |
| 18.35417906 | 0.662621694 | 0.740332804 | 17.61384626 | Uzbekistan |
| 18.63313795 | 0.596513059 | 0.966535454 | 17.6666025 | Venezuela (Bolivarian Republic of) |
| 20.26401082 | 0.4989451 | 1.645602776 | 18.61840804 | Mauritania |
| 19.29714916 | 0.686425621 | 0.62050884 | 18.67664032 | Tokelau |
| 22.16713455 | 0.384553634 | 3.334478595 | 18.83265595 | Malawi |
| 20.64693222 | 0.563775188 | 1.622241032 | 19.02469118 | El Salvador |
| 21.15612412 | 0.505413747 | 1.637167936 | 19.51895619 | Sao Tome and Principe |
| 20.66523782 | 0.725144495 | 0.503570286 | 20.16166753 | Kazakhstan |
| 21.31004447 | 0.650812335 | 0.825162845 | 20.48488162 | Guyana |
| 21.27241446 | 0.708864828 | 0.552164178 | 20.72025028 | Panama |
| 22.40184588 | 0.523768077 | 1.62562316 | 20.77622272 | Kenya |
| 21.66057332 | 0.694851274 | 0.629709674 | 21.03086365 | Azerbaijan |
| 22.90772162 | 0.513037248 | 1.629883289 | 21.27783833 | Honduras |
| 22.19151599 | 0.637195963 | 0.888040146 | 21.30347584 | Saint Vincent and the Grenadines |
| 24.10704155 | 0.40971416 | 2.682003285 | 21.42503826 | Gambia |
| 23.24446531 | 0.53390084 | 1.627191304 | 21.61727401 | Myanmar |
| 24.49882946 | 0.408533695 | 2.45185043 | 22.04697903 | Togo |
| 25.44882452 | 0.400246943 | 3.318682013 | 22.13014251 | Madagascar |
| 23.96001008 | 0.623004075 | 0.885558518 | 23.07445156 | Syrian Arab Republic |
| 23.56535895 | 0.754987055 | 0.430694172 | 23.13466477 | Saint Kitts and Nevis |
| 25.79695823 | 0.408054193 | 2.482032818 | 23.31492542 | Senegal |
| 42.88751519 | 0.268579941 | 19.39626304 | 23.49125215 | Mali |
| 24.56523902 | 0.655442913 | 0.790179856 | 23.77505916 | Colombia |
| 28.66437602 | 0.353109621 | 4.030670626 | 24.63370539 | Guinea-Bissau |
| 25.19459412 | 0.72622205 | 0.514845287 | 24.67974883 | Niue |
| 25.81992239 | 0.746748764 | 0.433667931 | 25.38625446 | Barbados |
| 47.13736748 | 0.240436019 | 21.38051398 | 25.7568535 | Chad |
| 26.65874042 | 0.651219329 | 0.835176359 | 25.82356406 | Philippines |
| 28.18411981 | 0.662054037 | 0.744725517 | 27.43939429 | Peru |
| 29.08167934 | 0.539972424 | 1.626400241 | 27.4552791 | Guatemala |
| 32.13548013 | 0.352442452 | 4.30134213 | 27.834138 | Liberia |
| 28.70387668 | 0.664575304 | 0.74638788 | 27.9574888 | Mexico |
| 30.41818395 | 0.487958371 | 1.683187389 | 28.73499656 | Djibouti |
| 31.8806694 | 0.56493039 | 1.411916141 | 30.46875326 | Ghana |
| 32.64703058 | 0.505948954 | 1.628541475 | 31.01848911 | Zambia |
| 34.46740276 | 0.403863943 | 3.316912529 | 31.15049023 | Eritrea |
| 43.33749775 | 0.336401293 | 11.89393052 | 31.44356723 | Guinea |
| 33.7395449 | 0.633665739 | 0.902469179 | 32.83707572 | Suriname |
| 35.34033224 | 0.425941883 | 2.003446627 | 33.33688561 | Côte d'Ivoire |
| 53.47351892 | 0.285118402 | 18.53202012 | 34.9414988 | Burkina Faso |
| 37.76984976 | 0.433174635 | 1.946523274 | 35.82332649 | Nepal |
| 36.79101182 | 0.599010799 | 0.932083514 | 35.85892831 | Bolivia (Plurinational State of) |
| 36.86864961 | 0.653043887 | 0.781342076 | 36.08730754 | Brazil |
| 48.66058189 | 0.326462614 | 12.24271663 | 36.41786526 | Mozambique |
| 39.47138176 | 0.435588706 | 1.825801983 | 37.64557978 | Rwanda |
| 40.16186775 | 0.492420885 | 1.663326276 | 38.49854148 | Bangladesh |
| 40.58253413 | 0.423261181 | 1.992171899 | 38.59036223 | Uganda |
| 40.72271305 | 0.541511187 | 1.628090098 | 39.09462295 | Tajikistan |
| 43.81648882 | 0.575401649 | 1.203204157 | 42.61328466 | India |
| 44.49008328 | 0.479691223 | 1.700992988 | 42.78909029 | Cameroon |
| 44.91903497 | 0.503390833 | 1.637814577 | 43.28122039 | Nigeria |
| 45.19862182 | 0.448278285 | 1.808590078 | 43.39003174 | Haiti |
| 48.56933619 | 0.358665881 | 3.886654774 | 44.68268141 | Sierra Leone |
| 46.54894863 | 0.475978688 | 1.712477446 | 44.83647118 | Comoros |
| 50.52511807 | 0.373486574 | 3.491351495 | 47.03376658 | Benin |
| 52.54609818 | 0.358823295 | 3.715227998 | 48.83087018 | Ethiopia |
| 53.96163813 | 0.446568273 | 1.790218122 | 52.17142001 | United Republic of Tanzania |
| 55.03270629 | 0.473062378 | 1.70639782 | 53.32630847 | Bhutan |
| 61.06278849 | 0.682160776 | 0.62788347 | 60.43490502 | Turkmenistan |
| 99.96339536 | 0.278371125 | 19.02284214 | 80.94055322 | South Sudan |
| 107.8644591 | 0.504028689 | 1.644629017 | 106.2198301 | Pakistan |
